# Hypertension screening, awareness, treatment, and control in India: A nationally representative cross-sectional study among individuals aged 15 to 49 years

**DOI:** 10.1371/journal.pmed.1002801

**Published:** 2019-05-03

**Authors:** Jonas Prenissl, Jennifer Manne-Goehler, Lindsay M. Jaacks, Dorairaj Prabhakaran, Ashish Awasthi, Anne Christine Bischops, Rifat Atun, Till Bärnighausen, Justine I. Davies, Sebastian Vollmer, Pascal Geldsetzer

**Affiliations:** 1 Heidelberg Institute of Global Health, Heidelberg University, Heidelberg, Germany; 2 Medical Faculty Mannheim, Heidelberg University, Mannheim, Germany; 3 Department of Global Health and Population, Harvard T.H. Chan School of Public Health, Boston, Massachusetts, United States of America; 4 Division of Infectious Diseases, Massachusetts General Hospital, Harvard Medical School, Boston, Massachusetts, United States of America; 5 Public Health Foundation of India, New Delhi, National Capital Region, India; 6 Harvard Medical School, Harvard University, Boston, Massachusetts, United States of America; 7 Africa Health Research Institute, Somkhele, KwaZulu-Natal, South Africa; 8 MRC/Wits Rural Public Health and Health Transitions Research Unit, School of Public Health, University of Witwatersrand, Johannesburg, South Africa; 9 Institute of Applied Health Research, University of Birmingham, Birmingham, United Kingdom; 10 Department of Economics, University of Göttingen, Göttingen, Germany; 11 Centre for Modern Indian Studies, University of Göttingen, Göttingen, Germany; Harvard University, UNITED STATES

## Abstract

**Background:**

Evidence on where in the hypertension care process individuals are lost to care, and how this varies among states and population groups in a country as large as India, is essential for the design of targeted interventions and to monitor progress. Yet, to our knowledge, there has not yet been a nationally representative analysis of the proportion of adults who reach each step of the hypertension care process in India. This study aimed to determine (i) the proportion of adults with hypertension who have been screened, are aware of their diagnosis, take antihypertensive treatment, and have achieved control and (ii) the variation of these care indicators among states and sociodemographic groups.

**Methods and findings:**

We used data from a nationally representative household survey carried out from 20 January 2015 to 4 December 2016 among individuals aged 15–49 years in all states and union territories (hereafter “states”) of the country. The stages of the care process—computed among those with hypertension at the time of the survey—were (i) having ever had one’s blood pressure (BP) measured before the survey (“screened”), (ii) having been diagnosed (“aware”), (iii) currently taking BP-lowering medication (“treated”), and (iv) reporting being treated and not having a raised BP (“controlled”). We disaggregated these stages by state, rural–urban residence, sex, age group, body mass index, tobacco consumption, household wealth quintile, education, and marital status. In total, 731,864 participants were included in the analysis. Hypertension prevalence was 18.1% (95% CI 17.8%–18.4%). Among those with hypertension, 76.1% (95% CI 75.3%–76.8%) had ever received a BP measurement, 44.7% (95% CI 43.6%–45.8%) were aware of their diagnosis, 13.3% (95% CI 12.9%–13.8%) were treated, and 7.9% (95% CI 7.6%–8.3%) had achieved control. Male sex, rural location, lower household wealth, and not being married were associated with greater losses at each step of the care process. Between states, control among individuals with hypertension varied from 2.4% (95% CI 1.7%–3.3%) in Nagaland to 21.0% (95% CI 9.8%–39.6%) in Daman and Diu. At 38.0% (95% CI 36.3%–39.0%), 28.8% (95% CI 28.5%–29.2%), 28.4% (95% CI 27.7%–29.0%), and 28.4% (95% CI 27.8%–29.0%), respectively, Puducherry, Tamil Nadu, Sikkim, and Haryana had the highest proportion of all adults (irrespective of hypertension status) in the sampled age range who had hypertension but did not achieve control. The main limitation of this study is that its results cannot be generalized to adults aged 50 years and older—the population group in which hypertension is most common.

**Conclusions:**

Hypertension prevalence in India is high, but the proportion of adults with hypertension who are aware of their diagnosis, are treated, and achieve control is low. Even after adjusting for states’ economic development, there is large variation among states in health system performance in the management of hypertension. Improvements in access to hypertension diagnosis and treatment are especially important among men, in rural areas, and in populations with lower household wealth.

## Abstract 2

Despite high levels of hypertension in India, screening awareness and treatment is poor, as revealed by large survey from Jonas Prenissl and colleagues.

## Author summary

**Why was this study done?:**

- Hypertension is a major risk factor for cardiovascular disease, which is the leading cause of death in India.
- The cascade of care for some chronic diseases—i.e., the proportion with a relevant condition who have ever been screened, are aware of their diagnosis, are on medication, and have achieved control—is a useful concept to inform intervention design and assess health system performance.
- To date, there has been little large-scale population-based evidence from India on the steps from screening for to successful control of hypertension at which people are lost from care.

**What did the researchers do and find?:**

- Using data from a nationally representative survey of 731,864 individuals aged 15 to 49 years sampled from all states and union territories in India, we constructed the hypertension care cascade by computing the percentage of participants with hypertension who reported ever having their blood pressure measured before the survey (“screened”), had previously been diagnosed with hypertension (“aware”), reported currently taking blood-pressure-lowering medication (“treated”), and were treated and had a normal blood pressure (“controlled”).
- Among those with hypertension, 76.1% had been screened, 44.7% were aware of their diagnosis, 13.3% were treated, and 7.9% had achieved control.
- In addition to a large degree of variation in the hypertension care cascade between states in India, we found that being male, living in a rural location, living in a less wealthy household, and not being married were associated with greater losses at each step of the care cascade.

**What do these findings mean?:**

- Whereas some states perform substantially better than others and thus may hold important policy lessons, across India most individuals aged 15 to 49 years with hypertension do not successfully transition through the steps of the care cascade.
- Interventions to improve hypertension care may want to target men, individuals in rural areas, and those with low household wealth, because these population groups were particularly likely to be lost at each step of the hypertension care cascade.
- An important limitation of this study is that the results cannot be generalized to adults aged 50 years and older, which is the population segment with the highest hypertension prevalence.

## Introduction

India is experiencing a rapid increase in noncommunicable diseases (NCDs) while still grappling with a high burden of infectious diseases and maternal and child health conditions [1]. Cardiovascular disease (CVD), which is the leading cause of disease burden as measured by disability-adjusted life years in the country, caused an estimated 25% of India’s NCD burden in 2016. Hypertension is a major risk factor for CVD, particularly ischemic heart disease and stroke [2,3]. While the prevalence of hypertension declined in many high-income countries from 1975 to 2015, it rose substantially in most low-income and middle-income countries, and especially in South Asia [4]. In a recent nationally representative study among 1.3 million adults in India, we found that 25% of adults had raised blood pressure (BP), with even young adults aged 18–25 years having a substantial prevalence, at 12% [5].

Antihypertensive medications are both inexpensive and efficacious [6,7]. Yet, only a minority of adults with hypertension in India are diagnosed and receive recommended treatment [8]. This lack of care for people with hypertension, combined with the rapid rise of CVD in India [9], requires concerted attention if the Sustainable Development Goal 3 target of reducing premature mortality from NCDs by 30% by 2030 is to be achieved [10]. An important first step in improving care for people with hypertension is to obtain a detailed understanding of current health system performance in managing hypertension—both as a benchmark to assess progress over time and to inform the design of appropriate health system interventions. Yet, to date, to our knowledge, studies examining management of hypertension in India have been carried out only in selected states or cities [8,11–13].

This is to our knowledge the first large-scale population-based study that examines health system performance in management of hypertension in India, and in each of its 29 states and 7 union territories. We use a “cascade of care” approach, which depicts where along the care process patients are lost to care, and which can powerfully illustrate what types of interventions (e.g., detection and diagnosis, promotion of medication adherence, or retention-in-care activities) are needed. Using data from a nationally representative sample of adults aged 15–49 years in India, this study, therefore, aims to (i) determine the cascade of care for hypertension in India and (ii) examine how it varies among states and union territories and population groups.

## Methods

### Data source

We used data from the 2015–2016 National Family Health Survey (NFHS-4), which is a household survey that covered each district in all 29 states and 7 union territories of India. The NFHS-4 was conducted under the stewardship of India’s Ministry of Health and Family Welfare and was managed by the International Institute for Population Sciences (IIPS), Mumbai [14]. ICF International provided technical assistance. The survey was supported financially by the US Agency for International Development and India’s Ministry of Health and Family Welfare. Data collection began on 20 January 2015 and ended on 4 December 2016. The NFHS-4 is representative both at the national level and at the level of the states and union territories.

The NFHS-4 sample was self-weighting at the level of the district. This was achieved in a 2-stage cluster random sampling approach by sampling the primary sampling units (villages in rural areas and census enumeration blocks in urban areas) with probability proportional to population size (using population estimates from the 2011 India census), and then sampling the same absolute number of households in each primary sampling unit [15]. Households were selected through systematic random sampling (i.e., sampling every *n*th household) after a complete mapping and household listing. The data collection team revisited households up to 3 times if no one was present in the household or an eligible household member was not available at the time of the household visit.

The NFHS-4 sampled more women than men because the survey had a focus on maternal and child health. Specifically, all non-pregnant women aged 15–49 years and—in a random sub-sample of 15% of households—men aged 15–54 years were eligible for the survey questionnaire and BP measurements. Men aged 50–54 years were excluded from this analysis to ensure an equal age range among women and men. The response rate (for both the questionnaire and the BP measurements) was 96.7% among women and 91.9% among men. More detail on the methodology of the NFHS-4 can be found in Methods A in S1 Supplemental Materials and in the official report of the NFHS-4 [16].

### Data collection

Prior to the main data collection phase of the survey, a pilot of the NFHS-4 was conducted, which consisted of 147 household interviews, 183 women’s interviews, 121 men’s interviews, and biomarker measurements (including BP) among 181 adults. In addition, three 1- to 2-week “training of trainers” courses were carried out by IIPS and ICF International in Puri (Odisha), Mumbai (Maharashtra), and Chandigarh (Chandigarh). The coordinators who participated as trainees in these courses were then responsible for the training of all fieldworkers in each of India’s states and union territories. In addition, all fieldworkers underwent a special physical measurement and biomarker training, which included taking accurate BP measurements. Specifically, the training consisted of role playing with other fieldworkers, practice at healthcare facilities under the supervision of healthcare workers, and initial supervision of measurements by more experienced fieldworkers during the main data collection phase. A detailed description of the biomarker measurement training and procedures can be found in the NFHS-4 biomarker questionnaire [17] and the biomarker manual distributed to each trainee [18].

The NFHS-4 team implemented several measures aimed at ensuring high data quality, which included (i) multiple levels of monitoring and supervision, including supervision by field agency district coordinators, IIPS project officers, staff and consultants from ICF International, and representatives from the Ministry of Health and Family Welfare; (ii) revisits by field supervisors of a random subset of participants to verify their questionnaire answers; and (iii) the use of computer-assisted personal interviewing, which allowed the supervising institutions to continuously monitor data collection progress and quality. Collected data were sent daily via an internet file streaming system to IIPS. Further details regarding the data collection process can be found in the official report of the NFHS-4 [16], the supervisor manual [19], the biomarker manual [18], and the interviewer manual [20].

### Ascertaining hypertension

Systolic and diastolic BP were measured 3 times (using a portable Omron BP monitor, model HEM-8712) in each individual on the same arm, with at least 5 minutes between each BP measurement and 5 minutes of sitting before the first measurement. We used the mean of the 3 BP measurements to calculate BP. If 1 measurement was missing for an individual in the dataset (2.3% of those with ≥1 measurement), we used the mean of the remaining 2 measurements. If 2 measurements were missing (1.5% of those with ≥1 measurement), we used the remaining measurement. Reasons for missing values were not given. Raised BP was defined as having a mean systolic BP ≥ 140 mm Hg or a mean diastolic BP ≥ 90 mm Hg [21]. We did not use the new American College of Cardiology/American Heart Association Task Force on Clinical Practice Guidelines threshold for stage 1 hypertension (systolic BP ≥ 130 mm Hg or diastolic BP ≥ 80 mm Hg) because this guideline was not used in clinical practice in India at the time of data collection for the NFHS-4 [22].

Hypertension was defined as having raised BP or responding with “yes” to at least 1 of the 2 following questions: (i) “Were you told on 2 or more different occasions by a doctor or other health professional that you had hypertension or high blood pressure?” (in line with most clinical guidelines that recommend confirming a high BP at a later time through a second BP measurement [22]) and (ii) “To lower your blood pressure, are you now taking a prescribed medicine?” [17]. These questions were asked of all participants regardless of their BP. Our hypertension definition differs from the one used in the official NFHS-4 report in that the NFHS-4 report did not include self-reported previous diagnosis of hypertension in its definition [16].

### Constructing the hypertension care cascade

The hypertension cascade was constructed only among those with hypertension (as per the definition above), whereby the denominator was the same for each step [23]. Specifically, participants with hypertension were considered to have been “screened” if they responded with “yes” to the question, “Before this survey, has your blood pressure ever been checked?” Participants were considered as being “aware” if they responded with “yes” to the question, “Were you told on two or more different occasions by a doctor or other health professional that you had hypertension or high blood pressure?” Participants were considered as being “treated” if they responded with “yes” to the question, “To lower your blood pressure, are you now taking a prescribed medicine?” We assumed that all those who were “treated” were also “aware.” Lastly, “controlled” hypertension was defined as being “treated” and having mean systolic BP < 140 mm Hg and diastolic BP < 90 mm Hg per the survey BP measurement. Those who were “aware” but, paradoxically, responded with “no” to the question “Before this survey, has your blood pressure ever been checked?” were excluded from the analysis. This was the case for 2.1% of those with hypertension. The unmet needs for the care outcomes “unscreened,” “unaware,” “untreated,” and “uncontrolled” were defined as the reciprocal values of “screened,” “aware,” “treated,” and “controlled,” respectively. The calculation of the percentage and total number of adults aged 15 to 49 years in each state/union territory who had unmet needs for each care indicator is described in Methods B in S1 Supplemental Materials.

### Predictors of reaching each care cascade step

We examined how the probability of reaching each step of the care cascade varied by the following variables: age, sex, body mass index (BMI), tobacco consumption (smoking tobacco, consuming smokeless tobacco), rural versus urban location, education, household wealth quintile, marital status (currently married or not), and state or union territory. Because the World Health Organization (WHO) considers the BMI cutoffs of ≥23.0 kg/m^2^ and ≥27.5 kg/m^2^ to be of public health significance in South Asian populations, in addition to the thresholds of ≥25.0 kg/m^2^ and ≥30.0 kg/m^2^ for overweight and obesity, we grouped BMI into the following categories: <18.5 kg/m^2^, 18.5–22.9 kg/m^2^, 23.0–24.9 kg/m^2^, 25.0–27.4 kg/m^2^, 27.5–29.9 kg/m^2^, and ≥30.0 kg/m^2^ [24–26]. Education was categorized as “primary school unfinished,” “primary school finished,” “secondary school unfinished,” and “secondary school finished or above.” Household wealth quintile was computed based on a household wealth index, which was created—using the methodology by Filmer and Pritchett [27]—separately for rural and urban areas. The household wealth index used data on 7 key household characteristics and household ownership of 25 durable goods. The creation of the household wealth index is described in more detail in Methods C in S1 Supplemental Materials.

### Statistical analysis

Sampling weights were computed to account for the survey design. We assigned a higher weight to male than female participants to adjust for the lower probability of sampling men (whereby we used the sex distribution of the Indian population by 1-year age group per the 2011 Indian census). The probability of reaching each cascade step was computed using sampling weights and disaggregated by the following variables: age group, sex, rural versus urban location, household wealth quintile, and state or union territory (hereafter “state”). Because financing hypertension care may be more feasible in richer than in poorer states, we plotted the state-level probability of reaching each cascade step against the state’s gross domestic product (GDP) per capita (in 2015 international dollars) to identify states that were performing well or poorly relative to their level of wealth.

To determine individual-level predictors of reaching each cascade step, we used a separate Poisson regression (with a robust error structure [28]) with a binary outcome (indicating whether or not the person reached the given cascade step) for each cascade step, whereby the sample for each regression was all individuals aged 15 to 49 years with hypertension. We preferred Poisson over logistic regression because odds ratios are frequently misinterpreted as risk ratios (RRs) [29], which matters when the outcome is common (as is the case in this analysis) because the RR then differs substantially from the odds ratio. In our primary regression approach, we categorized age, BMI, and the household wealth index to allow for an easier interpretation of the RRs. However, to avoid the loss of information from categorizing a continuous variable, we also show our regression results when using continuous age, BMI, and household wealth index in S1 Supplemental Materials, and plot the predicted probabilities from this regression in the main paper. For this analysis, we used restricted cubic splines with 5 knots for each of the 3 continuous variables. The knots were placed at the fifth, 27.5th, 50th, 72.5th, and 95th percentiles of each variable. All regression models in this paper included fixed effects for all 640 districts in India to filter out district-level effects on the outcome variables. We adjusted the standard errors in the regression models for clustering at the primary sampling unit level because primary sampling units were the largest sampling unit in the survey [30]. This was a complete case analysis. R software (version 3.3.2; R Foundation) was used for all statistical analyses.

None of the analyses presented in this paper were prespecified. The decision to display state-level care cascade indicators by state GDP per capita was made during data analysis. All other analyses were planned.

### Ethics

This analysis received a determination of “not human subjects research” by the institutional review board of the Harvard T.H. Chan School of Public Health on 9 May 2018 because the authors had access to de-identified data only. None of the authors were involved in the data collection of the NFHS-4. IIPS—the implementer of the NFHS-4—has made the micro-data of this survey publicly accessible through the Demographic and Health Surveys (DHS) Program (see Data Availability statement). As per standard DHS data access procedures, we registered our study with the DHS Program, which involved a brief description of our research project. The DHS Program then made the de-identified micro-data available to us for download. In the original survey, written informed consent was obtained from all participants prior to administering the questionnaires.

## Results

### Sample characteristics

The NFHS-4 household survey consisted of 749,119 participants (647,451 women and 101,668 men) when including only individuals aged 15–49 years and excluding pregnant women. Of these participants, 2.3% (17,255/749,119) had a missing BP measurement or response to the outcome-defining survey questions. Those with a missing outcome variable were more likely to be male, live in an urban area, have a higher educational attainment, and live in a household with more wealth than those with non-missing values (Table A, S1 Supplemental Materials). Participants with a missing outcome variable were excluded, resulting in a sample of 731,864 participants (633,608 women and 98,256 men) for the analysis. In total, 17.8% (unweighted) of participants had hypertension (Table 1), 49.4% were younger than 30 years, 32.4% did not finish primary school, 68.7% were married, and 29.6% lived in urban areas. None of the 731,864 included participants had missing values for any of the characteristics shown in Table 1, except for BMI (1,037 [0.1%] missing values).

**Table 1:** Sample characteristics.

Characteristic	Total	Female	Male
*n*	731,864	633,608	98,256
Hypertension, *n* (%)	131,391 (17.8)	109,051 (17.2)	19,210 (19.6)
Age group, *n* (%)			
15–19 years	132,088 (18.0)	114,050 (18.0)	18,038 (18.4)
20–24 years	116,457 (15.9)	100,864 (15.9)	15,593 (15.9)
25–29 years	113,704 (15.5)	98,500 (15.5)	15,204 (15.5)
30–34 years	102,979 (14.1)	89,095 (14.1)	13,884 (14.1)
35–39 years	99,510 (13.6)	86,222 (13.6)	13,288 (13.5)
40–44 years	85,713 (11.7)	74,220 (11.7)	11,493 (11.7)
45–49 years	81,413 (11.1)	70,657 (11.2)	10,756 (10.9)
Educational attainment, *n* (%)			
Primary school unfinished	237,147 (32.4)	219,028 (34.6)	18,119 (18.4)
Primary school finished	48,798 (6.7)	42,688 (6.7)	6,110 (6.2)
Secondary school unfinished	294,749 (40.3)	247,716 (39.1)	47,033 (47.9)
Secondary school finished or above	151,170 (20.7)	124,176 (19.6)	26,994 (27.5)
Household wealth quintile, *n* (%)			
Q1 (poorest)	135,076 (18.5)	117,958 (18.6)	17,118 (17.4)
Q2	145,393 (19.9)	126,221 (19.9)	19,172 (19.5)
Q3	150,958 (20.6)	130,756 (20.6)	20,202 (20.6)
Q4	148,534 (20.3)	127,965 (20.2)	20,569 (20.9)
Q5 (richest)	151,903 (20.8)	130,708 (20.6)	21,195 (21.6)
BMI, *n* (%)			
<18.5 kg/m^2^	159,909 (21.8)	140,762 (22.2)	19,147 (19.5)
18.5–22.9 kg/m^2^	341,033 (46.6)	294,001 (46.4)	47,032 (47.9)
23.0–24.9 kg/m^2^	95,585 (13.1)	80,437 (12.7)	15,148 (15.4)
25.0–27.4 kg/m^2^	68,964 (9.4)	58,975 (9.3)	9,989 (10.2)
27.5–29.9 kg/m^2^	35,225 (4.8)	31,034 (4.9)	4,191 (4.3)
≥30.0 kg/m^2^	30,111 (4.1)	27,562 (4.4)	2,549 (2.6)
*Missing*	1,037 (0.1)	837 (0.1)	200 (0.2)
Tobacco consumption, *n* (%)			
Current smoker	39,530 (5.4)	13,216 (2.1)	26,314 (26.8)
Uses smokeless tobacco	89,933 (12.3)	57,887 (9.1)	32,046 (32.6)
Currently married, *n* (%)	502,673 (68.7)	443,407 (70.0)	59,266 (60.3)
Urban residence, *n* (%)	216,382 (29.6)	185,538 (29.3)	30,844 (31.4)

Sample characteristics are not weighted using sampling weights.

### The hypertension care cascade at the national level

**Fig 1:**
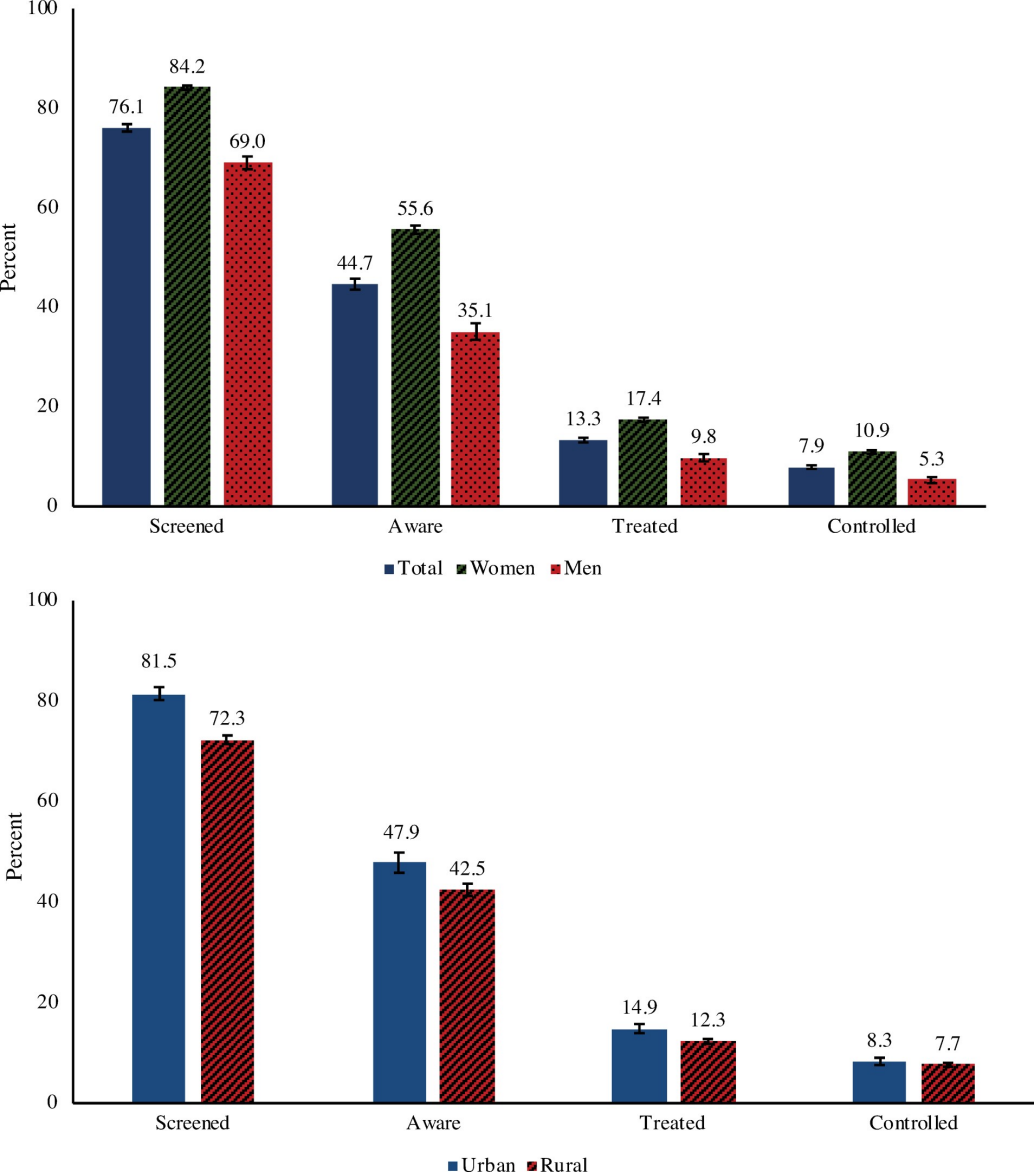
Percent of those with hypertension who reached each step of the care cascade by sex and rural–urban residence. Cascade indicators by 5-year age group are shown in Table C in S1 Supplemental Materials. A flowchart depiction of the hypertension care cascade is shown in Fig A in S1 Supplemental Materials.

The national prevalence of hypertension in the sampled age range was 18.1% (95% CI 17.8%–18.4%). Men had a somewhat higher prevalence than women (19.0%, 95% CI 18.5%–19.5%, compared to 17.2%, 95% CI 16.9%–17.4%). Hypertension prevalence estimates by age group and sex are shown in Table B in S1 Supplemental Materials. Among hypertensive individuals, 76.1% (95% CI 75.3%–76.8%) had ever received a BP measurement (“screened”), 44.7% (95% CI 43.6%–45.8%) had been diagnosed prior to the survey (“aware”), 13.3% (95% CI 12.9%–13.8%) reported currently taking a prescribed antihypertensive drug (“treated”), and 7.9% (95% CI 7.6%–8.3%) were on treatment and had a normal BP (“controlled”). Women and participants living in urban areas were more likely to reach each step of the care cascade (Fig 1).

### The hypertension care cascade by state

**Fig 2:**
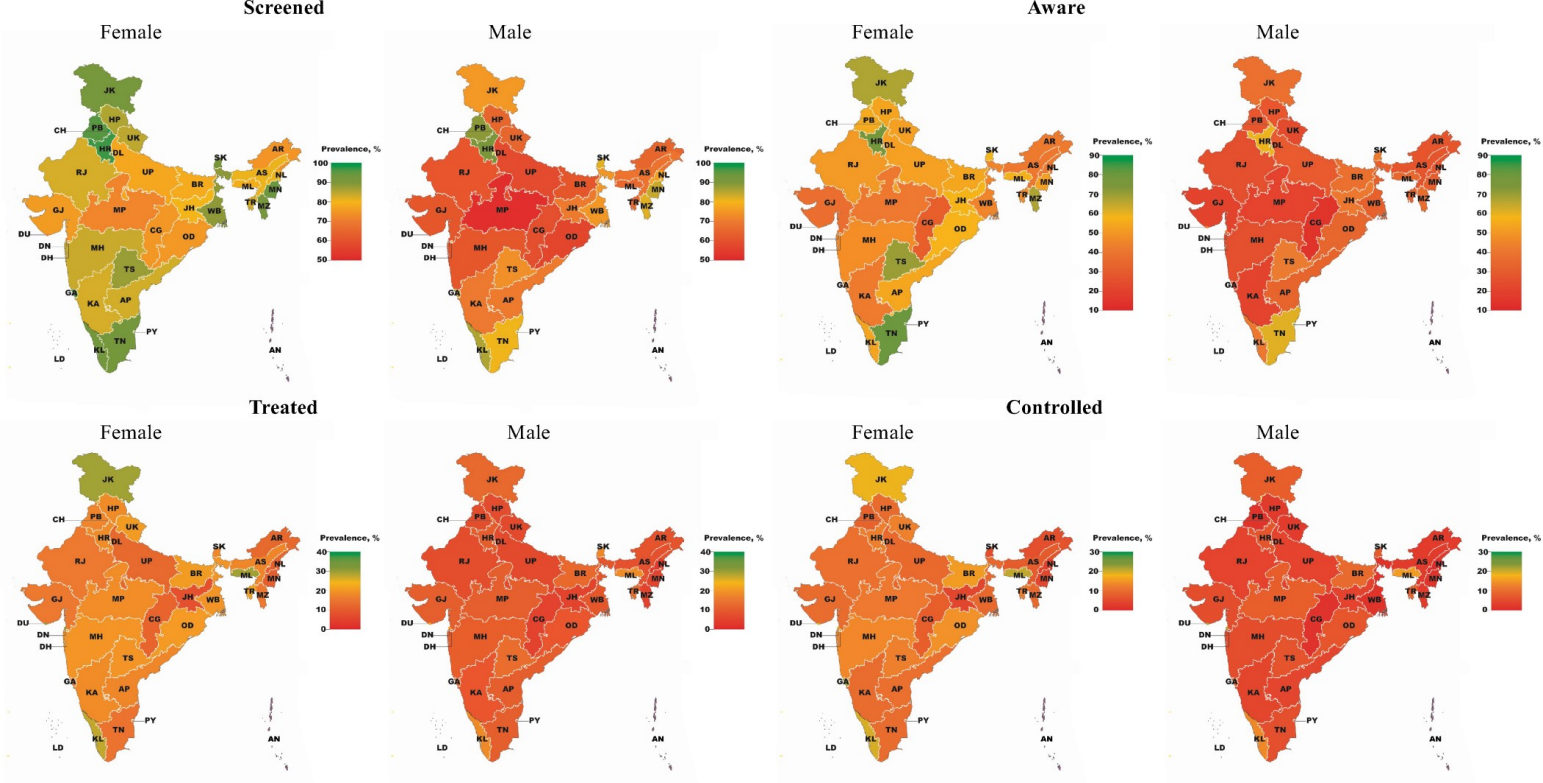
The hypertension care cascade by state. Union territories are included in the map but some are not visible due to their small size. Daman (DN) and Diu (DU) form one union territory together. Point estimates and 95% CIs for each state and union territory can be found in Tables D–H in S1 Supplemental Materials and, by sex, in Tables I–L in S1 Supplemental Materials. AN, Andaman and Nicobar Islands; AP, Andhra Pradesh; AR, Arunachal Pradesh; AS, Assam; BR, Bihar; CG, Chhattisgarh; CH, Chandigarh; DH, Dadra and Nagar Haveli; DL, Delhi; DN, Daman; DU, Diu; GA, Goa; GJ, Gujarat; HP, Himachal Pradesh; HR, Haryana; JH, Jharkhand; JK, Jammu and Kashmir; KA, Karnataka; KL, Kerala; LD, Lakshadweep; MH, Maharashtra; ML, Meghalaya; MN, Manipur; MP, Madhya Pradesh; MZ, Mizoram; NL, Nagaland; OD, Odisha (Orissa); PB, Punjab; PY, Puducherry; RJ, Rajasthan; SK, Sikkim; TN, Tamil Nadu; TR, Tripura; TS, Telangana State; UK, Uttarakhand (Uttaranchal); UP, Uttar Pradesh; WB, West Bengal.

Fig 2 depicts the variation among states in the proportion of those with hypertension who reached each step of the care cascade. Screening coverage among hypertensive individuals varied from 61.3% (95% CI 59.1%–63.3%) in Madhya Pradesh to 93.5% (95% CI 91.9%–94.9%) in Haryana; awareness of diagnosis, from 22.1% (95% CI 19.6%–24.9%) in Chhattisgarh to 80.5% (95% CI 69.8%–88.1%) in Puducherry; treated hypertension, from 7.1% (95% CI 5.9%–8.5%) in Jharkhand to 24.7% (95% CI 20.8%–29.9%) in Meghalaya; and controlled hypertension, from 2.4% (95% CI 1.7%–3.3%) in Nagaland to 21.0% (95% CI 9.8%–39.6%) in Daman and Diu (Tables D–G, S1 Supplemental Materials). Hypertension prevalence ranged from 12.8% (95% CI 12.0%–13.7%) in Bihar to 40.0% (95% CI 32.4%–48.1%) in Puducherry (Fig B and Table H, S1 Supplemental Materials).

**Fig 3:**
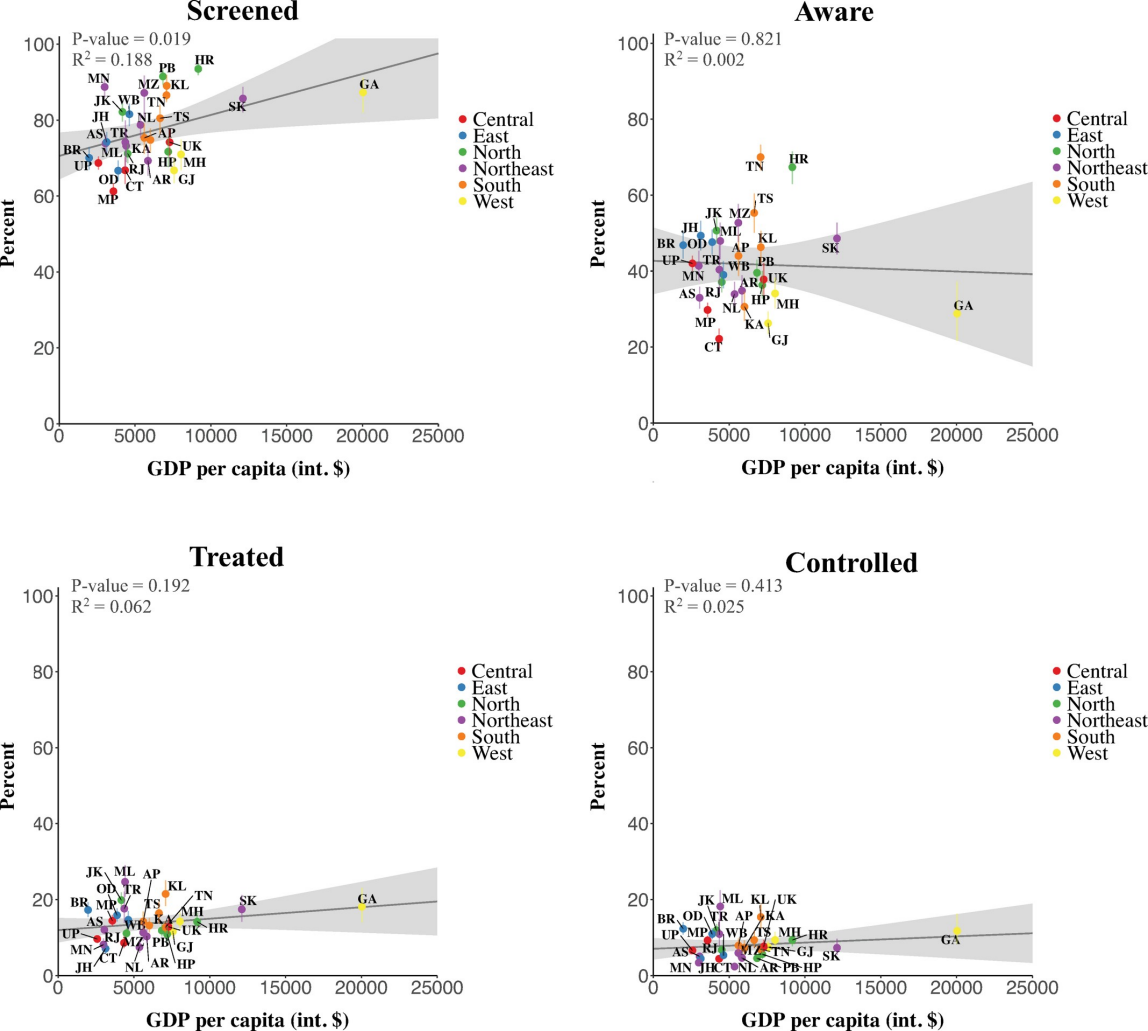
The association between state gross domestic product (GDP) per capita and each cascade step. State population estimates were obtained from the 2011 census of India, and GDP data (for 2013/2014) were obtained from the Central Statistics Office, Ministry of Statistics and Programme Implementation, Government of India [15,31]. Indian rupee values were converted to 2015 international dollars (int. $) using the purchasing power parity conversion factors published by the World Bank [32]. The grey ribbon depicts the pointwise 95% prediction interval. The vertical bars depict 95% confidence intervals. *P* values refer to the regression coefficient of the ordinary least squares regression line (with each state having the same weight) shown in the figure. Similarly, *R*^2^ values are for the ordinary least squares regression of states’ cascade achievement onto their GDP per capita. States were divided into regions as per their allocation to Zonal Councils by the Government of India [33]. Union territories were excluded from this figure because of the unavailability of GDP information for 3 of the 7 union territories. AP, Andhra Pradesh; AR, Arunachal Pradesh; AS, Assam; BR, Bihar; CT, Chhattisgarh; GA, Goa; GJ, Gujarat; HP, Himachal Pradesh; HR, Haryana; JH, Jharkhand; JK, Jammu and Kashmir; KA, Karnataka; KL, Kerala; MH, Maharashtra; ML, Meghalaya; MN, Manipur; MP, Madhya Pradesh; MZ, Mizoram; NL, Nagaland; OD, Odisha (Orissa); PB, Punjab; RJ, Rajasthan; SK, Sikkim; TN, Tamil Nadu; TR, Tripura; TS, Telangana State; UK, Uttarakhand (Uttaranchal); UP, Uttar Pradesh; WB, West Bengal.

States with higher GDP per capita tended to perform better on the screening cascade step (Fig 3). The only states that performed better in at least 3 out of 4 cascade steps than would be expected based on their GDP per capita were Jammu and Kashmir, and Kerala. Chhattisgarh and Nagaland were the only states that performed worse in at least 3 out of 4 cascade steps than predicted based on GDP per capita.

In Puducherry, 38.0% (95% CI 36.3%–38.9%) of the total population aged 15 to 49 years had uncontrolled hypertension (i.e., had hypertension but did not reach the “controlled” step of the hypertension care cascade), followed by Tamil Nadu (28.8%, 95% CI 28.5%–29.2%), Sikkim (28.4%, 95% CI 27.7%–29.0%), and Haryana (28.4%, 95% CI 27.8%–29.0%) (Tables M–Q, S1 Supplemental Materials). The highest absolute number of adults in this age range who had uncontrolled hypertension lived in Uttar Pradesh (14,267,516, 95% CI 14,162,771–14,363,058), Tamil Nadu (12,820,905, 95% CI 12,656,563–12,962,861), and Maharashtra (10,896,960, 95% CI 10,641,607–11,109,137) (Tables R–V, S1 Supplemental Materials).

### The hypertension care cascade in relation to individuals’ characteristics

Stratification of the hypertension cascade steps by age group, rural–urban residence, and household wealth quintile (Fig C, S1 Supplemental Materials) and covariate-adjusted Poisson regressions (Table 2 and Table W in S1 Supplemental Materials) show that (i) women had a higher probability than men of completing each step of the cascade; (ii) there was a positive association of “screened” (in urban areas) and “treated” with age group; (iii) urban areas performed better than rural areas for all cascade steps; (iv) being in a richer household wealth quintile was positively associated with completing each cascade step; (v) education was positively associated with being screened and being aware of one’s diagnosis but had no significant association with treatment and control; (vi) being obese (BMI ≥ 30.0 kg/m^2^) was associated with a higher likelihood of reaching the “treated” step and, in urban areas, also the “controlled” step; and (vii) tobacco consumption was generally not associated with substantial differences in the likelihood of progressing through the care cascade. In addition, those who were married had a higher likelihood of completing each care cascade step, with the association generally not differing between men and women (Table X, S1 Supplemental Materials). The regression results were similar when using sampling weights (Table Y, S1 Supplemental Materials), when running the analysis separately for men and women (Tables Z–AA, S1 Supplemental Materials), and when fitting covariate-unadjusted rather than covariate-adjusted regressions (Tables AB–AE, S1 Supplemental Materials).

**Table 2:** Predictors of having treated and controlled hypertension among adults with hypertension.

Characteristic	Treated hypertension				Controlled hypertension			
	Rural		Urban		Rural		Urban	
	RR (95% CI)	*P* value	RR (95% CI)	*P* value	RR (95% CI)	*P* value	RR (95% CI)	*P* value
Age group								
15–19 years	1.00 (reference)		1.00 (reference)		1.00 (reference)		1.00 (reference)	
20–24 years	0.90 (0.81–0.99)	0.032	0.98 (0.82–1.16)	0.784	0.88 (0.79–0.98)	0.017	0.98 (0.81–1.18)	0.809
25–29 years	0.94 (0.85–1.03)	0.193	0.88 (0.74–1.04)	0.133	0.89 (0.80–0.99)	0.036	0.82 (0.67–0.99)	0.035
30–34 years	0.96 (0.87–1.07)	0.473	1.01 (0.85–1.18)	0.952	0.84 (0.75–0.94)	0.002	0.81 (0.67–0.98)	0.028
35–40 years	1.06 (0.96–1.17)	0.267	1.16 (0.99–1.36)	0.067	0.78 (0.70–0.88)	<0.001	0.78 (0.64–0.94)	0.008
40–44 years	1.19 (1.08–1.31)	<0.001	1.53 (1.31–1.79)	<0.001	0.77 (0.69–0.86)	<0.001	0.94 (0.78–1.13)	0.518
45–49 years	1.38 (1.25–1.52)	<0.001	1.94 (1.65–2.26)	<0.001	0.79 (0.71–0.89)	<0.001	1.15 (0.96–1.38)	0.141
Education								
Primary school unfinished	1.00 (reference)		1.00 (reference)		1.00 (reference)		1.00 (reference)	
Primary school finished	1.01 (0.95–1.08)	0.652	1.05 (0.97–1.15)	0.243	1.02 (0.93–1.11)	0.726	1.07 (0.95–1.21)	0.264
Secondary school unfinished	1.03 (0.99–1.08)	0.151	0.98 (0.92–1.04)	0.503	1.06 (1.00–1.12)	0.064	0.98 (0.90–1.07)	0.643
Secondary school or above	1.00 (0.94–1.06)	0.974	0.96 (0.90–1.03)	0.293	1.06 (0.98–1.14)	0.158	0.97 (0.88–1.07)	0.583
Household wealth quintile								
Q1 (poorest)	1.00 (reference)		1.00 (reference)		1.00 (reference)		1.00 (reference)	
Q2	1.26 (1.17–1.36)	<0.001	1.11 (1.03–1.20)	0.009	1.21 (1.11–1.32)	<0.001	1.15 (1.03–1.28)	0.011
Q3	1.34 (1.24–1.44)	<0.001	1.17 (1.08–1.27)	<0.001	1.30 (1.19–1.42)	<0.001	1.15 (1.03–1.29)	0.012
Q4	1.44 (1.34–1.55)	<0.001	1.27 (1.17–1.38)	<0.001	1.38 (1.25–1.51)	<0.001	1.37 (1.22–1.54)	<0.001
Q5 (richest)	1.53 (1.42–1.66)	<0.001	1.34 (1.22–1.47)	<0.001	1.44 (1.30–1.59)	<0.001	1.40 (1.24–1.59)	<0.001
BMI								
<18.5 kg/m2	1.10 (1.04–1.16)	<0.001	1.09 (0.98–1.21)	0.128	1.19 (1.12–1.26)	<0.001	1.15 (1.01–1.30)	0.030
18.5–22.9 kg/m2	1.00 (reference)		1.00 (reference)		1.00 (reference)		1.00 (reference)	
23.0–24.9 kg/m2	1.02 (0.97–1.07)	0.448	1.01 (0.98–1.21)	0.767	0.86 (0.81–0.92)	<0.001	0.91 (0.83–1.00)	0.049
25.0–27.4 kg/m2	1.07 (1.01–1.12)	0.012	1.11 (1.04–1.19)	0.002	0.82 (0.77–0.88)	<0.001	0.90 (0.83–0.99)	0.030
27.5–29.9 kg/m2	1.17 (1.10–1.24)	<0.001	1.28 (1.19–1.37)	<0.001	0.86 (0.79–0.94)	0.001	0.98 (0.89–1.08)	0.658
≥30.0 kg/m2	1.46 (1.38–1.54)	<0.001	1.61 (1.51–1.71)	<0.001	1.04 (0.95–1.13)	0.405	1.23 (1.13–1.35)	<0.001
Tobacco consumption								
Current smoker	0.93 (0.86–1.01)	0.105	1.02 (0.91–1.14)	0.738	1.01 (0.90–1.13)	0.878	0.98 (0.82–1.15)	0.771
Uses smokeless tobacco	0.98 (0.92–1.04)	0.461	0.99 (0.92–1.07)	0.753	0.93 (0.86–1.01)	0.069	1.01 (0.90–1.13)	0.910
Currently married	1.11 (1.05–1.17)	<0.001	1.09 (1.02–1.16)	0.010	1.12 (1.04–1.20)	0.001	1.13 (1.03–1.24)	0.008
Female	1.78 (1.66–1.91)	<0.001	1.64 (1.51–1.79)	<0.001	2.01 (1.83–2.21)	<0.001	1.95 (1.72–2.20)	<0.001

These Poisson regressions contained all variables listed in the table (age group, household wealth quintile, education, BMI, tobacco consumption, marital status, and sex) and a binary indicator for each district (district-level fixed effects) as predictor variables. Standard errors were adjusted for clustering at the level of the primary sampling unit. Regression results including an interaction term between marital status and sex, using sampling weights (to account for the higher number of sampled women), and running analyses separately for women and men can be found in Tables X–AA in S1 Supplemental Materials. The sample for each regression was all individuals with hypertension (i.e., the sample was not restricted to those who had reached the preceding cascade step).

BMI, body mass index; RR, risk ratio.

**Fig 4:**
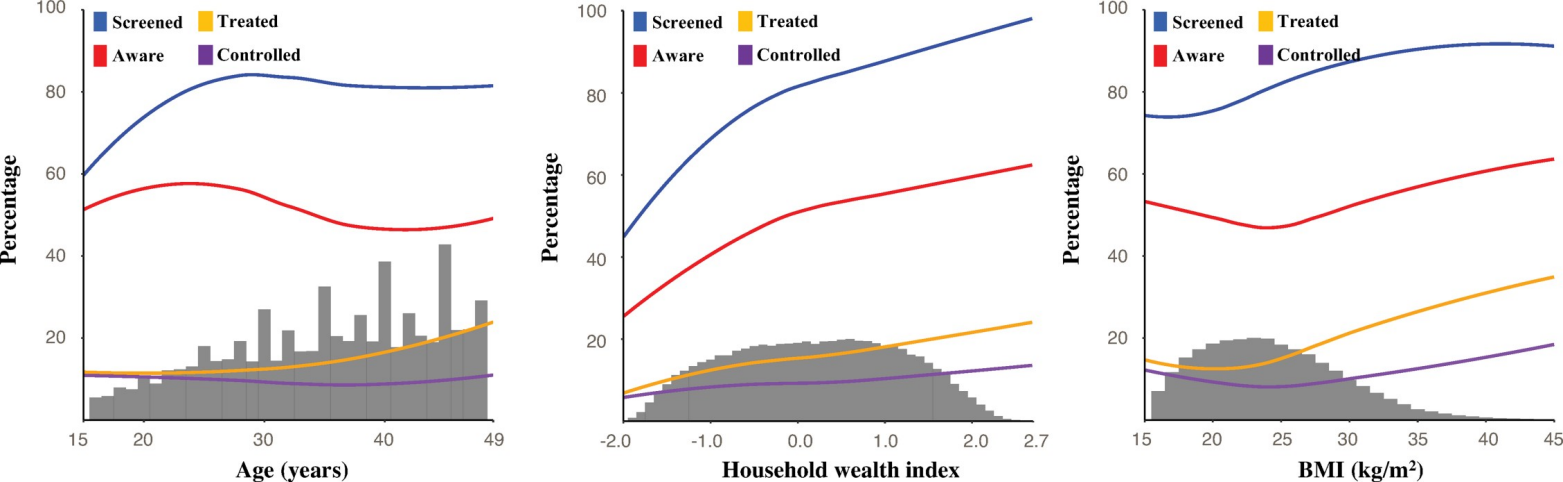
The predicted probability of reaching each cascade step by age, household wealth index, and BMI as continuous variables. Predicted probabilities were obtained from covariate-adjusted logistic regressions of hypertension care indicators on individuals’ sociodemographic characteristics (age, household wealth index, education, marital status, sex, rural versus urban location, BMI, tobacco consumption [smoking], and tobacco consumption [smokeless]) and district-level fixed effects. We used restricted cubic splines with 5 knots for each continuous variable (age, household wealth index, and BMI). Higher household wealth index values indicate more household wealth. A detailed description of all the household characteristics and assets included in this index can be found in Methods C in S1 Supplemental Materials. The grey histogram in the background represents the distribution of the variable on the *x*-axis, whereby the height of the histogram bars does not correspond to the percentages on the *y*-axis. The sample for each regression was all individuals with hypertension (i.e., the sample was not restricted to those who had reached the preceding cascade step).

Fig 4 shows that the probability of ever having had one’s BP measured increased with age until approximately age 25 years and that there appears to be an exponential positive association between age and the probability of being treated. The associations between age and the “aware” and “controlled” cascade steps are flat. Household wealth index has an approximately linear positive association with each cascade step. The association between BMI and each cascade step is flat or negative in the normal BMI range, and approximately linear and positive in the overweight and obese range. Table AF in S1 Supplemental Materials shows the RRs accompanying the predicted probabilities shown in Fig 4.

## Discussion

In this nationally representative sample of 731,864 adults aged 15–49 years in India, we found that while the proportion of those with hypertension who had ever had their BP measured was high (76%), less than half (45%) of individuals with the condition were aware of their diagnosis, less than 1 in 7 (13%) reported currently taking BP-lowering medication, and less than 1 in 10 (8%) had achieved control. Thus, the highest absolute losses to care occurred at the awareness (31.4 percentage points) and treatment (31.4 percentage points) stages, and the highest relative loss at the treatment stage (70.3%). While improvements are needed along the entire hypertension care cascade, this highlights a particular need for interventions that focus on the awareness and treatment steps of the cascade.

The country-level analysis of the hypertension care cascade, however, disguises the large variation in the care cascades among states and population groups. Specifically, we found worse values for each of the care cascade steps for men, those in lower household wealth quintiles, and those living in rural areas. These populations thus form important target groups for appropriate interventions, particularly because households with lower household wealth in rural areas are less likely to be able to access high-quality care for a CVD event, such as myocardial infarction, and more likely to experience catastrophic healthcare expenditures from such an event than their wealthier counterparts in urban areas.

Among states, we found that the proportion of adults with hypertension who were treated and whose hypertension was controlled varied by a factor of 3.5 and 8.8, respectively. Chhattisgarh and Nagaland performed worse than expected based on GDP per capita on at least 3 out of 4 cascade steps. Identifying and adopting some of the policies and programs that have allowed equally wealthy (or even poorer) states to perform better may well prove effective in increasing health system performance for managing hypertension in poorly performing states such as Chhattisgarh and Nagaland. “Role model” states could include Jammu and Kashmir and Kerala, which performed better than predicted based on GDP per capita in at least 3 out of 4 care cascade steps. Other states where improvements in managing adults with hypertension are particularly urgently needed are those in which a large proportion of the general population had hypertension but did not reach the control step of the care cascade (“uncontrolled hypertension”), such as Puducherry (38.0%), Tamil Nadu (28.8%), Sikkim (28.4%), and Haryana (28.4%). However, India’s states vary enormously in size, and thus large states with relatively low hypertension prevalence may still host a high absolute number of adults with uncontrolled hypertension. In fact, Uttar Pradesh, which had one of the lowest prevalence levels for hypertension in the observed age group (14.4%, 95% CI 13.9%–14.9%), had the highest estimated absolute number of adults (14,267,516) with uncontrolled hypertension. Lastly, a priority state for improving hypertension care should be Tamil Nadu, which had both the second highest proportion of adults aged 15 to 49 years with uncontrolled hypertension (28.8%) and the second highest absolute number of adults in that age range with uncontrolled hypertension (12,820,905).

While this analysis of data collected by the NFHS-4 team is to our knowledge the first large-scale population-based study of hypertension care in India, a systematic review and meta-analysis published in 2014 summarized 29 local studies in India that analyzed hypertension awareness, treatment, and control [8]. It estimated a lower probability of hypertension awareness (rural areas: 25.1%, 95% CI 21.0%–29.1%; urban areas: 41.9%, 95% CI 35.1%–48.9%) than this study (rural: 42.5%, 95% CI 41.2%–43.7%; urban: 47.9%, 95% CI 45.9%–49.9%). However, the estimates for both percentage treated (rural: 24.9%, 95% CI 16.7–33.0; urban: 37.6%, 95% CI 23.9%–51.2%) and percentage with controlled hypertension (rural: 10.7%, 95% CI 6.4%–15.0%; urban: 20.2%, 95% CI 11.6%–28.8%) were substantially higher than in this study (treated/rural: 12.3%, 95% CI 11.8%–12.8%; treated/urban: 14.9%, 95% CI 14.0%–15.8%; controlled/rural: 7.7%, 95% CI 7.3%–8.0%; controlled/urban: 8.3%, 95% CI 7.6%–9.0%). Several other local studies published since that review have also reported on these hypertension care indicators, with results that are similar to ours [13,34–37]. Our hypertension prevalence estimates are consistent with the most recent prevalence estimates for India among individuals aged 15 to 49 years, from an analysis of the Annual Health Survey and fourth District Level Household Survey [5]. In particular, this study also found a comparatively high hypertension prevalence, even among the youngest age group sampled (e.g., 9.6% [95% CI 9.3%–9.9%] among those aged 15 to 29 years). While these young individuals are unlikely to experience a CVD event in the near future, this finding suggests a worrying state of metabolic health among younger adults in India, which in turn may foreshadow a high CVD incidence in the coming decades as this cohort of younger adults reaches older ages.

The only cascade step reported on in the official NFHS-4 report is the proportion of those with diagnosed hypertension (in our analysis described as “aware”) who reported currently taking antihypertensive medications (treated). This conditional proportion is 29% in our analysis (see flowchart of Fig A in S1 Supplemental Materials) compared to “about one-third” in the NFHS-4 report [16]. The discrepancy might be due to the use of different sampling weights or minor changes in the dataset between the official NFHS-4 analysis and the dissemination of the data through the DHS Program. For full transparency, we have made all cleaning and analysis code of this study publicly accessible (see Data Availability statement). When using the hypertension definition employed by the NFHS-4 report (raised BP or reporting being treated), our hypertension prevalence estimates are very similar to those in the NFHS-4 report (11% and 12% for women and 15% and 16% for men in the NFHS-4 report and our analysis, respectively).

This study has several limitations. First, only adults aged 15–49 years were included in this analysis. Thus, our findings are not representative of the entire adult population in India. Nonetheless, given that India has a relatively young population, the age groups represented in this study accounted for 75.2% of all people in India aged ≥15 years in 2015 [38]. The lower age of participants in this sample is also largely responsible for the lower hypertension prevalence observed in this study compared to the nationally representative study among an older sample that our team published recently [5]. Second, because of the NFHS-4’s focus on maternal and child health, the survey sampled substantially fewer men than women. However, with 98,256 men included in the analysis, the absolute number of men sampled was still sufficient to obtain reasonably precise estimates of hypertension care cascade indicators among men. In addition, we adjusted our sampling weights to the sex distribution of India’s population in each 1-year age group to ensure that our estimates were representative for India’s adult population between the ages of 15 and 49 years despite the oversampling of women. Third, the definition of hypertension in this study was based on 3 BP measurements taken during 1 occasion, while a clinical diagnosis of hypertension requires raised BP measurements on at least 2 different occasions [22,39]. Falsely categorizing some adults as hypertensive who are normotensive would result in underestimates for “aware,” “treated,” and “controlled.” Fourth, 2.3% of participants had a missing value for at least 1 of the variables needed to define hypertension and each of the hypertension care cascade steps. While the percent missing is relatively small, those with a missing outcome variable are likely to have had a different probability of having hypertension and of reaching each step in the care cascade than those included in the analysis, as suggested by the fact that their sociodemographic characteristics were different (Table A, S1 Supplemental Materials). Fifth, the questions asked in the NFHS-4 questionnaire did not allow us to ascertain who among those diagnosed with hypertension had received relevant lifestyle advice. Given that our construction of the hypertension care cascade imposed the requirement that a participant must have reached all previous cascade steps to reach the next cascade step, only those who were treated could achieve control. Thus, participants who were diagnosed with hypertension and subsequently achieved hypertension control through lifestyle changes rather than medication were not considered to have controlled hypertension in this study. Our analysis, therefore, likely underestimates the percent of those with hypertension who achieved control. Lastly, the question “Before this survey, has your blood pressure ever been checked?” that defined the outcome “screened” does not quantify the number, or determine the regularity, of BP measurements received prior to the survey. Our estimate of “screened” should therefore be interpreted as the percentage who have ever had their BP measured (whether for hypertension screening or in other clinical interactions) rather than as the percentage who have been screened recently or on a regular basis since reaching a certain age.

In conclusion, the proportion of adults with hypertension in India who are aware of their diagnosis, are on treatment, and have the condition controlled is low. However, this study not only sets a benchmark for India as a whole to measure future progress; rather, by providing a detailed analysis of how the hypertension care cascade varies among population groups and states, this study can inform the selection of target groups and the design of appropriate interventions to improve hypertension care. In particular, India needs to urgently improve hypertension control among households with lower levels of wealth and those living in rural areas, which will likely need to include access to low-cost or free antihypertensive medications. A further important target group is men. Since India forms 18% of the world’s population and is expected to be the world’s most populous country by 2025, India’s ability to improve hypertension care will have a decisive impact on the world’s ability to achieve international NCD goals, including the Sustainable Development Goals and the WHO’s Global Action Plan for the Prevention and Control of NCDs [10,38,40].

## Supporting information

S1 Checklist STROBE checklist. (DOC)

S1 Supplemental Materials All supplemental text, tables, and figures. (DOCX)

## Abbreviations

BMI: body mass index
BP: blood pressure
CVD: cardiovascular disease
DHS: Demographic and Health Surveys
GDP: gross domestic product
IIPS: International Institute for Population Sciences
NCD: noncommunicable disease
NFHS-4: 2015–2016 National Family Health Survey
RR: risk ratio
