# Supplementary material for: Hypertension screening, awareness, treatment, and control in India: A nationally representative cross-sectional study among individuals aged 15 to 49 years
Source: PLoS Med. 2019 May 3;16(5):e1002801. doi: 10.1371/journal.pmed.1002801 (PMC6499417; doi:10.1371/journal.pmed.1002801)
Supplement: S1 Checklist — (DOC) [file pmed.1002801.s001.doc]

**S1 Checklist**

STROBE Statement—Checklist of items that should be included in reports of ***cross-sectional studies***

|  | Item No | Recommendation |
| --- | --- | --- |
| **Title and abstract** | 1 | (*a*) Indicate the study’s design with a commonly used term in the title or the abstract  **[Title and Abstract, para 2]**  Hypertension screening, awareness, treatment and control in India: a nationally representative cross-sectional study among individuals aged 15 to 49 years  **Methods and Findings:** We used data from a nationally representative household survey carried out from January 20^th^ 2015 to December 4^th^ 2016 among individuals aged 15-49 years in all states and Union Territories of the country. The stages of the care process – computed among those with hypertension – were: i) having ever had your blood pressure (BP) measured (‘screened’), ii) having been diagnosed (‘aware’), iii) currently taking BP-lowering medication (‘treated’), and iv) reporting to be treated and not having a raised BP (‘controlled’). We disaggregated these stages by state, rural-urban residence, sex, age group, Body Mass Index (BMI), tobacco consumption, household wealth quintile, education, and marital status. 731,864 participants were included in the analysis. Hypertension prevalence was 18.1% (95% CI, 17.8% - 18.4%). Among those with hypertension, 76.1% (95% CI, 75.3% - 76.8%) had ever received a BP measurement, 44.7% (95% CI, 43.6% - 45.8%) were aware of their diagnosis, 13.3% (95% CI, 12.9% - 13.8%) were treated, and 7.9% (95% CI, 7.6% - 8.3%) had achieved control. Male sex, rural location, less household wealth, and not being married were associated with greater losses at each step of the care process. Between states, control among individuals with hypertension varied from 2.4% (95% CI, 1.7% - 3.3%) in Nagaland to 21.0% (95% CI, 9.8% - 39.6%) in Daman and Diu. At 38.0% (95% CI, 36.3% - 39.0%), 28.8% (95% CI, 28.5% - 29.2%), 28.4% (95% CI, 27.7% - 29.0%), and 28.4% (95% CI, 27.8% - 29.0%), respectively, Puducherry, Tamil Nadu, Sikkim, and Haryana had the highest proportion of all adults (whether hypertensive or not) in the sampled age range who had hypertension but did not achieve control. The main limitation of this study is that its results cannot be generalized to adults aged 50 years and older – the population group in which hypertension is most common. |
|  |  | (*b*) Provide in the abstract an informative and balanced summary of what was done and what was found  **[Abstract, para 2]**  **Methods and Findings:** We used data from a nationally representative household survey carried out from January 20^th^ 2015 to December 4^th^ 2016 among individuals aged 15-49 years in all states and Union Territories of the country. The stages of the care process – computed among those with hypertension – were: i) having ever had your blood pressure (BP) measured (‘screened’), ii) having been diagnosed (‘aware’), iii) currently taking BP-lowering medication (‘treated’), and iv) reporting to be treated and not having a raised BP (‘controlled’). We disaggregated these stages by state, rural-urban residence, sex, age group, Body Mass Index (BMI), tobacco consumption, household wealth quintile, education, and marital status. 731,864 participants were included in the analysis. Hypertension prevalence was 18.1% (95% CI, 17.8% - 18.4%). Among those with hypertension, 76.1% (95% CI, 75.3% - 76.8%) had ever received a BP measurement, 44.7% (95% CI, 43.6% - 45.8%) were aware of their diagnosis, 13.3% (95% CI, 12.9% - 13.8%) were treated, and 7.9% (95% CI, 7.6% - 8.3%) had achieved control. Male sex, rural location, less household wealth, and not being married were associated with greater losses at each step of the care process. Between states, control among individuals with hypertension varied from 2.4% (95% CI, 1.7% - 3.3%) in Nagaland to 21.0% (95% CI, 9.8% - 39.6%) in Daman and Diu. At 38.0% (95% CI, 36.3% - 39.0%), 28.8% (95% CI, 28.5% - 29.2%), 28.4% (95% CI, 27.7% - 29.0%), and 28.4% (95% CI, 27.8% - 29.0%), respectively, Puducherry, Tamil Nadu, Sikkim, and Haryana had the highest proportion of all adults (whether hypertensive or not) in the sampled age range who had hypertension but did not achieve control. The main limitation of this study is that its results cannot be generalized to adults aged 50 years and older – the population group in which hypertension is most common. |
| Introduction | | |
| Background/rationale | 2 | Explain the scientific background and rationale for the investigation being reported  **[Introduction: paragraph 1 and 2]**  India is experiencing a rapid increase in non-communicable diseases (NCDs) while still grappling with a high burden of infectious diseases and maternal and child health conditions.[1] Cardiovascular and cerebrovascular disease (CVD), which is the leading cause of disease burden as measured by disability-adjusted life years in the country, caused an estimated 25% of India’s NCD burden in 2016. Hypertension is a major risk factor for CVD, particularly ischemic heart disease and strokes.[2,3] While the prevalence of hypertension has declined in many high-income countries from 1975 to 2015, it has risen substantially in most low-income and middle-income countries (LMICs), and especially in South Asia.[4] In a recent nationally representative study among 1.3 million adults in India, we found that 25% of adults had a raised BP, with even young adults aged 18 - 25 years having a substantial prevalence of 12%.[5]  Antihypertensive medications are both inexpensive and efficacious.[6,7] Yet, only a minority of adults with hypertension in India is diagnosed and receives recommended treatment.[8] This lack of care for people with hypertension, combined with the rapid rise of CVD in India,[9] requires a concerted effort if the Sustainable Development Goal (SDG) 3 target of reducing premature mortality from NCDs by 30% by 2030 is to be achieved.[10] An important first step in improving care for people with hypertension is to obtain a detailed understanding of current health system performance in managing hypertension – both as a benchmark to assess progress over time and to inform the design of appropriate health system interventions. Yet, to date, studies examining management of hypertension in India have only been carried out in selected states or cities.[8,11-13] |
| Objectives | 3 | State specific objectives, including any prespecified hypotheses **[Introduction: paragraph 3]**  This is the first large-scale population-based study that examines health system performance in management of hypertension in India, and each of its 29 states and seven Union Territories. We use a ‘cascade of care’ approach, which depicts where along the care process patients are lost to care and can powerfully illustrate what type of interventions (e.g., detection and diagnosis, promotion of medication adherence, or retention-in-care activities) are needed. Using data from a nationally representative sample of adults aged 15-49 years in India, this study, therefore, aims to i) determine the cascade of care for hypertension in India, and ii) examine how it varies among states and population subgroups. |
| Methods | | |
| Study design | 4 | Present key elements of study design early in the paper  **[Methods: “Data sources” subsection, paragraph 1, 2 and 3]**  **Data source:**  We used data from the 2015 - 16 National Family Health Survey (NFHS-4), which is a household survey that covered each district in all 29 states and seven Union Territories of India. The NFHS-4 was conducted under the stewardship of India’s Ministry of Health and Family Welfare and managed by the International Institute for Population Sciences, Mumbai (IIPS).[14] ICF International, USA provided technical assistance. The survey was supported financially by the United States Agency for International Development and India’s Ministry of Health and Family Welfare. Data collection began on January 20^th^ 2015 and ended on December 4^th^ 2016. The NFHS-4 is representative both at the national level and at the level of the states and Union Territories.  The NFHS-4 sample was self-weighting at the level of the district. This was achieved in a two-stage cluster random sampling approach by sampling the primary sampling units (villages in rural areas or census enumeration blocks in urban areas) with probability proportional to population size (using population estimates from the 2011 India census), and then sampling the same absolute number of households in each primary sampling unit (PSU).[15] Households were selected through systematic random sampling (i.e., sampling every n^th^ household) after a complete mapping and household listing. The data collection team revisited households up to three times if no one was present in the household or an eligible household member was not available at the time of the household visit.  The NFHS-4 sampled more women than men because the survey had a focus on maternal and child health. Specifically, all non-pregnant women aged 15 - 49 years and – in a random sub-sample of 15% of households – men aged 15 - 54 years were eligible for the survey questionnaire and blood pressure (BP) measurements. Men aged 50 - 54 years were excluded from this analysis to ensure an equal age range among women and men. The response rate (for both the questionnaire and the BP measurements) was 96.7% among women and 91.9% among men. More detail on the methodology of the NFHS-4 can be found in Methods C (S1) and in the official report of the NFHS-4.[16] |
| Setting | 5 | Describe the setting, locations, and relevant dates, including periods of recruitment, exposure, follow-up, and data collection  **[Methods: “Data source” subsection, paragraph 1, 2 and 3; Methods: "Data collection" subsection, paragraph 1 and 2]**  **Data source:**  We used data from the 2015 - 16 National Family Health Survey (NFHS-4), which is a household survey that covered each district in all 29 states and seven Union Territories of India. The NFHS-4 was conducted under the stewardship of India’s Ministry of Health and Family Welfare and managed by the International Institute for Population Sciences, Mumbai (IIPS).[14] ICF International, USA provided technical assistance. The survey was supported financially by the United States Agency for International Development and India’s Ministry of Health and Family Welfare. Data collection began on January 20^th^ 2015 and ended on December 4^th^ 2016. The NFHS-4 is representative both at the national level and at the level of the states and Union Territories.  The NFHS-4 sample was self-weighting at the level of the district. This was achieved in a two-stage cluster random sampling approach by sampling the primary sampling units (villages in rural areas or census enumeration blocks in urban areas) with probability proportional to population size (using population estimates from the 2011 India census), and then sampling the same absolute number of households in each primary sampling unit (PSU).[15] Households were selected through systematic random sampling (i.e., sampling every n^th^ household) after a complete mapping and household listing. The data collection team revisited households up to three times if no one was present in the household or an eligible household member was not available at the time of the household visit.  The NFHS-4 sampled more women than men because the survey had a focus on maternal and child health. Specifically, all non-pregnant women aged 15 - 49 years and – in a random sub-sample of 15% of households – men aged 15 - 54 years were eligible for the survey questionnaire and blood pressure (BP) measurements. Men aged 50 - 54 years were excluded from this analysis to ensure an equal age range among women and men. The response rate (for both the questionnaire and the BP measurements) was 96.7% among women and 91.9% among men. More detail on the methodology of the NFHS-4 can be found in Methods C (S1) and in the official report of the NFHS-4.[16]  **Data collection:**  Prior to the main data collection phase of the survey, a pilot of the NFHS-4 was conducted, which consisted of 147 household interviews, 183 women’s interviews, 121 men’s interviews, and biomarker measurements (including BP) among 181 adults. In addition, three one- to two-week “training of trainers” courses were carried out by IIPS and ICF International in Puri (Odisha), Mumbai (Maharashtra), and Chandigarh (Chandigarh). The coordinators who participated as trainees in these courses were then responsible for the training of all fieldworkers in each of India’s states and Union Territories. In addition, all fieldworkers underwent a special physical measurement and biomarker training, which included taking accurate BP measurements. Specifically, the training consisted of role playing with other fieldworkers, practice at healthcare facilities under the supervision of healthcare workers, and initial supervision of measurements by more experienced field workers during the main data collection phase. A detailed description of the biomarker measurement training and procedures can be found in the NFHS-4 biomarker manual distributed to each trainee and the biomarker questionnaire.[17,18]  The NFHS-4 team implemented several measures aimed at ensuring high data quality, which included i) multiple levels of monitoring and supervision, including supervision by field agency district coordinators, IIPS project officers, staff and consultants from ICF International, and representatives from the Ministry of Health and Family Welfare; ii) revisits by field supervisors of a random subset of participants to verify their questionnaire answers; and iii) the use of computer-assisted personal interviewing (CAPI), which allowed the supervising institutions to continuously monitor data collection progress and quality. Collected data was sent daily via the Internet File Streaming System to IIPS. Further details regarding the data collection process can be found in the official report of the NFHS-4, the supervisor manual, the biomarker manual, and the interviewer manual.[16,18-20] |
| Participants | 6 | (*a*) Give the eligibility criteria, and the sources and methods of selection of participants  **[Methods: “Data sources” subsection, paragraph 1, 2 and 3 and Methods A, S1]**  **Data source:**  We used data from the 2015 - 16 National Family Health Survey (NFHS-4), which is a household survey that covered each district in all 29 states and seven Union Territories of India. The NFHS-4 was conducted under the stewardship of India’s Ministry of Health and Family Welfare and managed by the International Institute for Population Sciences, Mumbai (IIPS).[14] ICF International, USA provided technical assistance. The survey was supported financially by the United States Agency for International Development and India’s Ministry of Health and Family Welfare. Data collection began on January 20^th^ 2015 and ended on December 4^th^ 2016. The NFHS-4 is representative both at the national level and at the level of the states and Union Territories.  The NFHS-4 sample was self-weighting at the level of the district. This was achieved in a two-stage cluster random sampling approach by sampling the primary sampling units (villages in rural areas or census enumeration blocks in urban areas) with probability proportional to population size (using population estimates from the 2011 India census), and then sampling the same absolute number of households in each primary sampling unit (PSU).[15] Households were selected through systematic random sampling (i.e., sampling every n^th^ household) after a complete mapping and household listing. The data collection team revisited households up to three times if no one was present in the household or an eligible household member was not available at the time of the household visit.  The NFHS-4 sampled more women than men because the survey had a focus on maternal and child health. Specifically, all non-pregnant women aged 15 - 49 years and – in a random sub-sample of 15% of households – men aged 15 - 54 years were eligible for the survey questionnaire and blood pressure (BP) measurements. Men aged 50 - 54 years were excluded from this analysis to ensure an equal age range among women and men. The response rate (for both the questionnaire and the BP measurements) was 96.7% among women and 91.9% among men. More detail on the methodology of the NFHS-4 can be found in Methods C (S1) and in the official report of the NFHS-4.[16] Methods A. Methodology of the NFHS-4 The NFHS-4 was designed to yield precise health indicators at multiple geographic levels (district, state/union territory, national) and for different areas (urban/rural, slum/non-slum areas in large cities). A stratified two-stage random sampling design was employed. Primary sampling units (PSUs) were defined as villages in rural areas and Census Enumeration Blocks (CEBs) in urban areas according to the 2011 Census for India. Small PSUs (fewer than 40 households) were added to the nearest PSU. Strata were rural and urban areas within each of India’s 640 districts. Villages in each rural stratum and CEBs in each urban stratum were selected with probability proportional to population size (PPS). In rural areas, six substrata within each stratum were created by crossing three substrata (defined based on the estimated number of households in each village) with two substrata (defined based on the percentage of the population belonging to scheduled castes and tribes). PSUs with more than 300 estimated households were divided into segments of 100-150 households, and two of these segments were randomly selected (again, using PPS) for the survey. In each of these clusters (PSUs or PSU segments), 22 households were randomly selected using systematic random sampling.  Four Computer Assisted Personal Interviewing (CAPI) questionnaires (Household Questionnaire, Woman’s Questionnaire, Man’s Questionnaire, and Biomarker Questionnaire) were administered in the local language by trained interviewers. The training procedure has been described in the main text.  In the Household Questionnaire, all usual members of the household and visitors who stayed in the household the night before the interview were listed and written consent for the survey was obtained from each individual. Basic demographic information including age, sex, marital status, schooling, relationship to the head of the household was collected for each listed person.  Personal interviews using the Woman's and Man's Questionnaire as well as physical measurements using the Biomarker Questionnaire were only conducted among listed persons of age 15-49 years (Women) or 15-54 years (Men). The Woman's Questionnaire, Man's Questionnaire and the Biomarker Questionnaire contained the key measures used in this study. More detailed information on the sampling procedure, the questionnaire items and the execution of the survey can be found in the formal report of the NFHS-4, the published questionnaires and the multiple manuals that were distributed to the staff, which are all available online.[1-8] |
| Variables | 7 | Clearly define all outcomes, exposures, predictors, potential confounders, and effect modifiers. Give diagnostic criteria, if applicable  **[Methods: “Data sources” subsection, paragraph 1, 2 and 3; Methods: “Ascertaining hypertension” subsection, paragraph 1 and 2; Methods: “Constructing the hypertension care cascade” subsection, paragraph 1]**  **Data source:**  We used data from the 2015 - 16 National Family Health Survey (NFHS-4), which is a household survey that covered each district in all 29 states and seven Union Territories of India. The NFHS-4 was conducted under the stewardship of India’s Ministry of Health and Family Welfare and managed by the International Institute for Population Sciences, Mumbai (IIPS).[14] ICF International, USA provided technical assistance. The survey was supported financially by the United States Agency for International Development and India’s Ministry of Health and Family Welfare. Data collection began on January 20^th^ 2015 and ended on December 4^th^ 2016. The NFHS-4 is representative both at the national level and at the level of the states and Union Territories.  The NFHS-4 sample was self-weighting at the level of the district. This was achieved in a two-stage cluster random sampling approach by sampling the primary sampling units (villages in rural areas or census enumeration blocks in urban areas) with probability proportional to population size (using population estimates from the 2011 India census), and then sampling the same absolute number of households in each primary sampling unit (PSU).[15] Households were selected through systematic random sampling (i.e., sampling every n^th^ household) after a complete mapping and household listing. The data collection team revisited households up to three times if no one was present in the household or an eligible household member was not available at the time of the household visit.  The NFHS-4 sampled more women than men because the survey had a focus on maternal and child health. Specifically, all non-pregnant women aged 15 - 49 years and – in a random sub-sample of 15% of households – men aged 15 - 54 years were eligible for the survey questionnaire and blood pressure (BP) measurements. Men aged 50 - 54 years were excluded from this analysis to ensure an equal age range among women and men. The response rate (for both the questionnaire and the BP measurements) was 96.7% among women and 91.9% among men. More detail on the methodology of the NFHS-4 can be found in Methods C (S1) and in the official report of the NFHS-4.[16].  **Ascertaining hypertension:**  Systolic and diastolic BP was measured three times (using the portable Omron BP monitor, model HEM - 8712) in each individual on the same arm with at least five minutes between each BP measurement and five minutes of sitting before the first measurement. We used the mean of the three BP measurements to calculate BP. If one measurement was missing in the dataset (2.3% of those for whom not all three measurements were missing), we used the mean of the remaining two measurements. If two measurements were missing (1.5% of those for whom not all three measurements were missing), we used the remaining measurement. Reasons for missing values were not given. Raised BP was defined as having a mean systolic BP ≥140mmHg or a mean diastolic BP ≥90mmHg.[21] We did not use the new American College of Cardiology/American Heart Association (ACC/AHA) Task Force on Clinical Practice Guidelines threshold for stage 1 hypertension (systolic BP ≥130 mmHg or diastolic BP ≥80 mmHg) because this guideline was not used in clinical practice in India at the time of data collection for the NFHS-4.[22]  Hypertension was defined as having raised BP or having responded with ‘yes’ to at least one of the two following questions: i) “Were you told on two or more different occasions by a doctor or other health professional that you had hypertension or high blood pressure?” (in line with most clinical guidelines that recommend confirming a high BP at a later time through a second BP measurement [22]), and ii) “To lower your blood pressure, are you now taking a prescribed medicine?”.[17] These questions were asked of all participants regardless of their BP. Our hypertension definition differed from the one used in the official NFHS-4 report in that the NFHS-4 report did not include a self-reported previous diagnosis of hypertension in its definition.[16]  **Constructing the hypertension care cascade:**  The hypertension cascade was constructed only among those with hypertension (as per the definition above) whereby the denominator was the same for each step.[23] Specifically, participants with hypertension were considered to have been ‘screened’ if they responded with ‘yes’ to the question, “Before this survey, has your blood pressure ever been checked?”. Participants were considered as being ‘aware’ if they responded in the affirmative to the question, “Were you told on two or more different occasions by a doctor or other health professional that you had hypertension or high blood pressure?”. Participants were considered as having been ‘treated’ if they responded with ‘yes’ to the question, “To lower your blood pressure, are you now taking a prescribed medicine?”. We assumed that all those who were ‘treated’ were also ‘aware’. Lastly, ‘controlled’ hypertension was defined as being ‘treated’ and having a systolic BP <140mmHg and diastolic BP <90mmHg. Those who were ‘aware’ but, paradoxically, responded with ‘no’ to the question “Before this survey, has your blood pressure ever been checked?” were excluded from the analysis. This was the case for 2.1% of those with hypertension. The unmet need for care outcomes ‘unscreened’, ‘unaware’, ‘untreated’, and ‘uncontrolled’ were defined as the reciprocal values of ‘screened’, ‘aware’, ‘treated’, and ‘controlled’, respectively. The calculation of the percentage and total number of adults aged 15 to 49 years in a state who had each unmet need for care indicator is described in Methods B (S1). |
| Data sources/ measurement | 8 | For each variable of interest, give sources of data and details of methods of assessment (measurement). Describe comparability of assessment methods if there is more than one group  **[Methods: “Ascertaining hypertension”, paragraph 1; Methods: “Predictors of reaching each cascade step” subsection, paragraph 1]**  **Ascertaining hypertension:**  Systolic and diastolic BP was measured three times (using the portable Omron BP monitor, model HEM - 8712) in each individual on the same arm with at least five minutes between each BP measurement and five minutes of sitting before the first measurement. We used the mean of the three BP measurements to calculate BP. If one measurement was missing in the dataset (2.3% of those for whom not all three measurements were missing), we used the mean of the remaining two measurements. If two measurements were missing (1.5% of those for whom not all three measurements were missing), we used the remaining measurement. Reasons for missing values were not given. Raised BP was defined as having a mean systolic BP ≥140mmHg or a mean diastolic BP ≥90mmHg.[21] We did not use the new American College of Cardiology/American Heart Association (ACC/AHA) Task Force on Clinical Practice Guidelines threshold for stage 1 hypertension (systolic BP ≥130 mmHg or diastolic BP ≥80 mmHg) because this guideline was not used in clinical practice in India at the time of data collection for the NFHS-4.[22]  Hypertension was defined as having raised BP or having responded with ‘yes’ to at least one of the two following questions: i) “Were you told on two or more different occasions by a doctor or other health professional that you had hypertension or high blood pressure?” (in line with most clinical guidelines that recommend confirming a high BP at a later time through a second BP measurement [22]), and ii) “To lower your blood pressure, are you now taking a prescribed medicine?”.[17] These questions were asked of all participants regardless of their BP. Our hypertension definition differed from the one used in the official NFHS-4 report in that the NFHS-4 report did not include a self-reported previous diagnosis of hypertension in its definition.[16]  **Constructing the hypertension care cascade:**  The hypertension cascade was constructed only among those with hypertension (as per the definition above) whereby the denominator was the same for each step.[23] Specifically, participants with hypertension were considered to have been ‘screened’ if they responded with ‘yes’ to the question, “Before this survey, has your blood pressure ever been checked?”. Participants were considered as being ‘aware’ if they responded in the affirmative to the question, “Were you told on two or more different occasions by a doctor or other health professional that you had hypertension or high blood pressure?”. Participants were considered as having been ‘treated’ if they responded with ‘yes’ to the question, “To lower your blood pressure, are you now taking a prescribed medicine?”. We assumed that all those who were ‘treated’ were also ‘aware’. Lastly, ‘controlled’ hypertension was defined as being ‘treated’ and having a systolic BP <140mmHg and diastolic BP <90mmHg. Those who were ‘aware’ but, paradoxically, responded with ‘no’ to the question “Before this survey, has your blood pressure ever been checked?” were excluded from the analysis. This was the case for 2.1% of those with hypertension. The unmet need for care outcomes ‘unscreened’, ‘unaware’, ‘untreated’, and ‘uncontrolled’ were defined as the reciprocal values of ‘screened’, ‘aware’, ‘treated’, and ‘controlled’, respectively. The calculation of the percentage and total number of adults aged 15 to 49 years in a state who had each unmet need for care indicator is described in Methods B (S1).    **Predictors of reaching each care cascade step:**  We examined how the probability of reaching each step of the care cascade varied by the following variables: age, sex, Body Mass Index (BMI), tobacco consumption (smokes tobacco, consumes smokeless tobacco), rural vs. urban location, education, household wealth quintile, marital status (currently married or not), and state (or Union Territory). Because the World Health Organization (WHO) considers the BMI cut-offs of ≥23.0 kg/m^2^ and ≥27.5kg/m^2^ to be of public health significance in South Asian populations in addition to the thresholds of ≥25.0 kg/m^2^ and ≥30.0 kg/m^2^ for overweight and obesity, we grouped BMI into the following categories: <18.5kg/m^2^, 18.5 - 22.9 kg/m^2^, 23.0 - 24.9 kg/m^2^, 25.0 - 27.4 kg/m^2^, 27.5 - 29.9 kg/m^2^, and ≥30.0 kg/m^2^.[24-26] Education was categorized as “Primary school unfinished”, “Primary school finished”, “Secondary school unfinished”, and “Secondary school or above”. Household wealth quintile was computed based on a household wealth index, which was created – using the methodology by Filmer and Pritchett – separately for rural and urban areas.[27] The household wealth index used data of seven key household characteristics and household ownership of 25 durable goods. The creation of the household wealth index is described in more detail in Methods C (S1). |
| Bias | 9 | Describe any efforts to address potential sources of bias **[Methods: “Statistical analysis” subsection, paragraph 1, 2 and 3]**  **Statistical analysis:**  Sampling weights were computed to account for the survey design. We assigned a higher weight to male than female participants to adjust for the lower probability of sampling men (whereby we used the sex distribution of the Indian population by one-year age group as per the 2011 Indian census). The probability of reaching each cascade step was computed using sampling weights and disaggregated by the following variables: age group, sex, rural vs. urban location, household wealth quintile, and state (or Union Territory). Because financing hypertension care may be more feasible in richer than poorer states, we plotted the state-level probability of reaching each cascade step against the state’s Gross Domestic Product (GDP) per capita (in 2015 international dollars) to identify states that were performing well or poorly respective to their level of wealth.  To determine individual-level predictors of reaching each cascade step, we used a separate Poisson regression (with a robust error structure [28]) with a binary outcome (indicating whether or not the person reached the given cascade step) for each cascade step whereby the sample for each regression was all individuals aged 15 to 49 years with hypertension. We preferred Poisson over logistic regression because Odds Ratios (ORs) are frequently misinterpreted as Risk Ratios (RRs),[29] which matters when the outcome is common (as is the case in this analysis) because the RR then differs substantially from the OR. In our primary regression approach, we categorized age, BMI, and the household wealth index to allow for an easier interpretation of the RRs. However, to avoid the loss of information from categorizing a continuous variable, we also show our regression results when using continuous age, BMI, and household wealth index in the appendix, and plotted the predicted probabilities from this regression in the main manuscript. For this analysis, we used restricted cubic splines with five knots for each of the three continuous variables. The knots were placed at the fifth, 27.5^th^, 50^th^, 72.5^th^, and 95^th^ percentiles of each variable. All regression models in this manuscript included fixed effects for all 640 districts in India to filter out district-level effects on the outcome variables. We adjusted the standard errors in the regression models for clustering at the PSU level because they were the largest sampling unit in the survey.[30] This was a complete case analysis. R software (version 3.3.2; R Foundation) was used for all statistical analyses.  None of the analyses presented in this manuscript were pre-specified. The decision to display state-level care cascade indicators by a state’s GDP per capita was made during data analysis. All other analyses were planned. |
| Study size | 10 | Explain how the study size was arrived at  **[Methods: “Data sources”, paragraph 1 - 3; Methods S1, Results: "Sample characteristics", paragraph 1]**  **Data source:**  We used data from the 2015 - 16 National Family Health Survey (NFHS-4), which is a household survey that covered each district in all 29 states and seven Union Territories of India. The NFHS-4 was conducted under the stewardship of India’s Ministry of Health and Family Welfare and managed by the International Institute for Population Sciences, Mumbai (IIPS).[14] ICF International, USA provided technical assistance. The survey was supported financially by the United States Agency for International Development and India’s Ministry of Health and Family Welfare. Data collection began on January 20^th^ 2015 and ended on December 4^th^ 2016. The NFHS-4 is representative both at the national level and at the level of the states and Union Territories.  The NFHS-4 sample was self-weighting at the level of the district. This was achieved in a two-stage cluster random sampling approach by sampling the primary sampling units (villages in rural areas or census enumeration blocks in urban areas) with probability proportional to population size (using population estimates from the 2011 India census), and then sampling the same absolute number of households in each primary sampling unit (PSU).[15] Households were selected through systematic random sampling (i.e., sampling every n^th^ household) after a complete mapping and household listing. The data collection team revisited households up to three times if no one was present in the household or an eligible household member was not available at the time of the household visit.  The NFHS-4 sampled more women than men because the survey had a focus on maternal and child health. Specifically, all non-pregnant women aged 15 - 49 years and – in a random sub-sample of 15% of households – men aged 15 - 54 years were eligible for the survey questionnaire and blood pressure (BP) measurements. Men aged 50 - 54 years were excluded from this analysis to ensure an equal age range among women and men. The response rate (for both the questionnaire and the BP measurements) was 96.7% among women and 91.9% among men. More detail on the methodology of the NFHS-4 can be found in Methods C (S1) and in the official report of the NFHS-4.[16] Methods A. Methodology of the NFHS-4 The NFHS-4 was designed to yield precise health indicators at multiple geographic levels (district, state/union territory, national) and for different areas (urban/rural, slum/non-slum areas in large cities). A stratified two-stage random sampling design was employed. Primary sampling units (PSUs) were defined as villages in rural areas and Census Enumeration Blocks (CEBs) in urban areas according to the 2011 Census for India. Small PSUs (fewer than 40 households) were added to the nearest PSU. Strata were rural and urban areas within each of India’s 640 districts. Villages in each rural stratum and CEBs in each urban stratum were selected with probability proportional to population size (PPS). In rural areas, six substrata within each stratum were created by crossing three substrata (defined based on the estimated number of households in each village) with two substrata (defined based on the percentage of the population belonging to scheduled castes and tribes). PSUs with more than 300 estimated households were divided into segments of 100-150 households, and two of these segments were randomly selected (again, using PPS) for the survey. In each of these clusters (PSUs or PSU segments), 22 households were randomly selected using systematic random sampling.  Four Computer Assisted Personal Interviewing (CAPI) questionnaires (Household Questionnaire, Woman’s Questionnaire, Man’s Questionnaire, and Biomarker Questionnaire) were administered in the local language by trained interviewers. The training procedure has been described in the main text.  In the Household Questionnaire, all usual members of the household and visitors who stayed in the household the night before the interview were listed and written consent for the survey was obtained from each individual. Basic demographic information including age, sex, marital status, schooling, relationship to the head of the household was collected for each listed person.  Personal interviews using the Woman's and Man's Questionnaire as well as physical measurements using the Biomarker Questionnaire were only conducted among listed persons of age 15-49 years (Women) or 15-54 years (Men). The Woman's Questionnaire, Man's Questionnaire and the Biomarker Questionnaire contained the key measures used in this study. More detailed information on the sampling procedure, the questionnaire items and the execution of the survey can be found in the formal report of the NFHS-4, the published questionnaires and the multiple manuals that were distributed to the staff, which are all available online.[1-8]  **Sample characteristics:**  The NFHS - 4 household survey consisted of 749,119 participants (647,451 women and 101,668 men) when only including individuals aged 15-49 years and excluding pregnant women. 2.3% (17,255/749,119) of these participants had a missing BP measurement or response to the outcome-defining survey questions. Those with a missing outcome variable were more likely to be male, live in an urban area, have a higher educational attainment, and live in a household with more wealth than those with non-missing values (Table A, S1). Participants with a missing outcome variable were excluded, resulting in a sample of 731,864 participants (633,608 women and 98,256 men) for the analysis. 17.8% (unweighted) of participants had hypertension (Table 1). 49.4% were younger than 30 years, 32.4% did not finish primary school, 68.7% were married, and 29.6% lived in urban areas. None of the 731,864 included participants had missing values for any of the characteristics shown in Table 1, except for BMI (1,037 [0.1%] missing values). |
| Quantitative variables | 11 | Explain how quantitative variables were handled in the analyses. If applicable, describe which groupings were chosen and why  **[Methods: “Predictors of reaching each cascade step” subsection, paragraph 1; Methods: “Statistical analysis” subsection, paragraph 1 - 3]**  **Predictors of reaching each care cascade step:**  We examined how the probability of reaching each step of the care cascade varied by the following variables: age, sex, Body Mass Index (BMI), tobacco consumption (smokes tobacco, consumes smokeless tobacco), rural vs. urban location, education, household wealth quintile, marital status (currently married or not), and state (or Union Territory). Because the World Health Organization (WHO) considers the BMI cut-offs of ≥23.0 kg/m^2^ and ≥27.5kg/m^2^ to be of public health significance in South Asian populations in addition to the thresholds of ≥25.0 kg/m^2^ and ≥30.0 kg/m^2^ for overweight and obesity, we grouped BMI into the following categories: <18.5kg/m^2^, 18.5 - 22.9 kg/m^2^, 23.0 - 24.9 kg/m^2^, 25.0 - 27.4 kg/m^2^, 27.5 - 29.9 kg/m^2^, and ≥30.0 kg/m^2^.[24-26] Education was categorized as “Primary school unfinished”, “Primary school finished”, “Secondary school unfinished”, and “Secondary school or above”. Household wealth quintile was computed based on a household wealth index, which was created – using the methodology by Filmer and Pritchett – separately for rural and urban areas.[27] The household wealth index used data of seven key household characteristics and household ownership of 25 durable goods. The creation of the household wealth index is described in more detail in Methods C (S1).  **Statistical analysis:**  Sampling weights were computed to account for the survey design. We assigned a higher weight to male than female participants to adjust for the lower probability of sampling men (whereby we used the sex distribution of the Indian population by one-year age group as per the 2011 Indian census). The probability of reaching each cascade step was computed using sampling weights and disaggregated by the following variables: age group, sex, rural vs. urban location, household wealth quintile, and state (or Union Territory). Because financing hypertension care may be more feasible in richer than poorer states, we plotted the state-level probability of reaching each cascade step against the state’s Gross Domestic Product (GDP) per capita (in 2015 international dollars) to identify states that were performing well or poorly respective to their level of wealth.  To determine individual-level predictors of reaching each cascade step, we used a separate Poisson regression (with a robust error structure [28]) with a binary outcome (indicating whether or not the person reached the given cascade step) for each cascade step whereby the sample for each regression was all individuals aged 15 to 49 years with hypertension. We preferred Poisson over logistic regression because Odds Ratios (ORs) are frequently misinterpreted as Risk Ratios (RRs),[29] which matters when the outcome is common (as is the case in this analysis) because the RR then differs substantially from the OR. In our primary regression approach, we categorized age, BMI, and the household wealth index to allow for an easier interpretation of the RRs. However, to avoid the loss of information from categorizing a continuous variable, we also show our regression results when using continuous age, BMI, and household wealth index in the appendix, and plotted the predicted probabilities from this regression in the main manuscript. For this analysis, we used restricted cubic splines with five knots for each of the three continuous variables. The knots were placed at the fifth, 27.5^th^, 50^th^, 72.5^th^, and 95^th^ percentiles of each variable. All regression models in this manuscript included fixed effects for all 640 districts in India to filter out district-level effects on the outcome variables. We adjusted the standard errors in the regression models for clustering at the PSU level because they were the largest sampling unit in the survey.[30] This was a complete case analysis. R software (version 3.3.2; R Foundation) was used for all statistical analyses.  None of the analyses presented in this manuscript were pre-specified. The decision to display state-level care cascade indicators by a state’s GDP per capita was made during data analysis. All other analyses were planned. |
| Statistical methods | 12 | (*a*) Describe all statistical methods, including those used to control for confounding **[Methods: “Statistical analysis” subsection, paragraph 1 and 2]** |
|  |  | (*b*) Describe any methods used to examine subgroups and interactions **[Methods: “Statistical analysis” subsection, paragraph 1 and 2]** |
|  |  | (*c*) Explain how missing data were addressed **[Methods: “Statistical analysis” subsection, paragraph 3]** |
|  |  | (*d*) If applicable, describe analytical methods taking account of sampling strategy **[Methods: “Statistical analysis” subsection, paragraph 1]** |
|  |  | (*e*) Describe any sensitivity analyses **[Not applicable]**  **ALL: Methods: “Statistical analysis” subsection, paragraph 1 - 3**  **Statistical analysis:**  Sampling weights were computed to account for the survey design. We assigned a higher weight to male than female participants to adjust for the lower probability of sampling men (whereby we used the sex distribution of the Indian population by one-year age group as per the 2011 Indian census). The probability of reaching each cascade step was computed using sampling weights and disaggregated by the following variables: age group, sex, rural vs. urban location, household wealth quintile, and state (or Union Territory). Because financing hypertension care may be more feasible in richer than poorer states, we plotted the state-level probability of reaching each cascade step against the state’s Gross Domestic Product (GDP) per capita (in 2015 international dollars) to identify states that were performing well or poorly respective to their level of wealth.  To determine individual-level predictors of reaching each cascade step, we used a separate Poisson regression (with a robust error structure [28]) with a binary outcome (indicating whether or not the person reached the given cascade step) for each cascade step whereby the sample for each regression was all individuals aged 15 to 49 years with hypertension. We preferred Poisson over logistic regression because Odds Ratios (ORs) are frequently misinterpreted as Risk Ratios (RRs),[29] which matters when the outcome is common (as is the case in this analysis) because the RR then differs substantially from the OR. In our primary regression approach, we categorized age, BMI, and the household wealth index to allow for an easier interpretation of the RRs. However, to avoid the loss of information from categorizing a continuous variable, we also show our regression results when using continuous age, BMI, and household wealth index in the appendix, and plotted the predicted probabilities from this regression in the main manuscript. For this analysis, we used restricted cubic splines with five knots for each of the three continuous variables. The knots were placed at the fifth, 27.5^th^, 50^th^, 72.5^th^, and 95^th^ percentiles of each variable. All regression models in this manuscript included fixed effects for all 640 districts in India to filter out district-level effects on the outcome variables. We adjusted the standard errors in the regression models for clustering at the PSU level because they were the largest sampling unit in the survey.[30] This was a complete case analysis. R software (version 3.3.2; R Foundation) was used for all statistical analyses.  None of the analyses presented in this manuscript were pre-specified. The decision to display state-level care cascade indicators by a state’s GDP per capita was made during data analysis. All other analyses were planned. |
| Results | | |
| Participants | 13* | (a) Report numbers of individuals at each stage of study—eg numbers potentially eligible, examined for eligibility, confirmed eligible, included in the study, completing follow-up, and analysed  **[Results: “Sample characteristics” subsection, paragraph 1; Table 1]**  **Sample characteristics:**  The NFHS - 4 household survey consisted of 749,119 participants (647,451 women and 101,668 men) when only including individuals aged 15-49 years and excluding pregnant women. 2.3% (17,255/749,119) of these participants had a missing BP measurement or response to the outcome-defining survey questions. Those with a missing outcome variable were more likely to be male, live in an urban area, have a higher educational attainment, and live in a household with more wealth than those with non-missing values (Table A, S1). Participants with a missing outcome variable were excluded, resulting in a sample of 731,864 participants (633,608 women and 98,256 men) for the analysis. 17.8% (unweighted) of participants had hypertension (Table 1). 49.4% were younger than 30 years, 32.4% did not finish primary school, 68.7% were married, and 29.6% lived in urban areas. None of the 731,864 included participants had missing values for any of the characteristics shown in Table 1, except for BMI (1,037 [0.1%] missing values).  *Table 1. Sample characteristics*^a^   | **Characteristic** | **Total** | **Female** | **Male** | | --- | --- | --- | --- | | n | 731,864 | 633,608 | 98,256 | | Hypertension, n (%) | 131,391 (17.8) | 109,051 (17.2) | 19,210 (19.6) | | Age Group, n (%), y |  |  |  | | 15 - 19 | 132,088 (18.0) | 114,050 (18.0) | 18,038 (18.4) | | 20 - 24 | 116,457 (15.9) | 100,864 (15.9) | 15,593 (15.9) | | 25 - 29 | 113,704 (15.5) | 98,500 (15.5) | 15,204 (15.5) | | 30 - 34 | 102,979 (14.1) | 89,095 (14.1) | 13,884 (14.1) | | 35 - 39 | 99,510 (13.6) | 86,222 (13.6) | 13,288 (13.5) | | 40 - 44 | 85,713 (11.7) | 74,220 (11.7) | 11,493 (11.7) | | 45 - 49 | 81,413 (11.1) | 70,657 (11.2) | 10,756 (10.9) | | Educational attainment, n (%) |  |  |  | | Primary school unfinished | 237,147 (32.4) | 219,028 (34.6) | 18,119 (18.4) | | Primary school finished | 48,798 (6.7) | 42,688 (6.7) | 6,110 ( 6.2) | | Secondary school unfinished | 294,749 (40.3) | 247,716 (39.1) | 47,033 (47.9) | | Secondary school finished or above | 151,170 (20.7) | 124,176 (19.6) | 26,994 (27.5) | | Household wealth quintile, n (%) |  |  |  | | Q1 (Poorest) | 135,076 (18.5) | 117,958 (18.6) | 17,118 (17.4) | | Q2 | 145,393 (19.9) | 126,221 (19.9) | 19,172 (19.5) | | Q3 | 150,958 (20.6) | 130,756 (20.6) | 20,202 (20.6) | | Q4 | 148,534 (20.3) | 127,965 (20.2) | 20,569 (20.9) | | Q5 (Richest) | 151,903 (20.8) | 130,708 (20.6) | 21,195 (21.6) | | BMI, n (%) |  |  |  | | <18.5kg/m^2^ | 159,909 (21.8) | 140,762 (22.2) | 19,147 (19.5) | | 18.5 - 22.9 kg/m^2^ | 341,033 (46.6) | 294,001 (46.4) | 47,032 (47.9) | | 23.0 - 24.9 kg/m^2^ | 95,585 (13.1) | 80,437 (12.7) | 15,148 (15.4) | | 25.0 - 27.4 kg/m^2^ | 68,964 (9.4) | 58,975 (9.3) | 9,989 (10.2) | | 27.5 - 29.9 kg/m^2^ | 35,225 (4.8) | 31,034 (4.9) | 4,191 (4.3) | | ≥ 30.0 kg/m^2^ | 30,111 (4.1) | 27,562 (4.4) | 2,549 (2.6) | | *Missing* | 1,037 (0.1) | 837 (0.1) | 200 (0.2) | | Tobacco consumption, n (%) |  |  |  | | Current smoker | 39,530 (5.4) | 13,216 (2.1) | 26,314 (26.8) | | Uses smokeless tobacco | 89,933 (12.3) | 57,887 (9.1) | 32,046 (32.6) | | Currently married, n (%) | 502,673 (68.7) | 443,407 (70.0) | 59,266 (60.3) | | Urban area, n (%) | 216,382 (29.6) | 185,538 (29.3) | 30,844 (31.4) |   Abbreviations: n=number; y=years; Q=quintile.  ^a^Sample characteristics were not weighted using sampling weights. |
|  |  | (b) Give reasons for non-participation at each stage **[Not applicable]** |
|  |  | (c) Consider use of a flow diagram **[Not applicable]** |
| Descriptive data | 14* | (a) Give characteristics of study participants (eg demographic, clinical, social) and information on exposures and potential confounders  **[Results: “Sample characteristics” subsection, paragraph 1; Table 1]**  **Sample characteristics:**  The NFHS - 4 household survey consisted of 749,119 participants (647,451 women and 101,668 men) when only including individuals aged 15-49 years and excluding pregnant women. 2.3% (17,255/749,119) of these participants had a missing BP measurement or response to the outcome-defining survey questions. Those with a missing outcome variable were more likely to be male, live in an urban area, have a higher educational attainment, and live in a household with more wealth than those with non-missing values (Table A, S1). Participants with a missing outcome variable were excluded, resulting in a sample of 731,864 participants (633,608 women and 98,256 men) for the analysis. 17.8% (unweighted) of participants had hypertension (Table 1). 49.4% were younger than 30 years, 32.4% did not finish primary school, 68.7% were married, and 29.6% lived in urban areas. None of the 731,864 included participants had missing values for any of the characteristics shown in Table 1, except for BMI (1,037 [0.1%] missing values).  *Table 1. Sample characteristics*^a^   | **Characteristic** | **Total** | **Female** | **Male** | | --- | --- | --- | --- | | n | 731,864 | 633,608 | 98,256 | | Hypertension, n (%) | 131,391 (17.8) | 109,051 (17.2) | 19,210 (19.6) | | Age Group, n (%), y |  |  |  | | 15 - 19 | 132,088 (18.0) | 114,050 (18.0) | 18,038 (18.4) | | 20 - 24 | 116,457 (15.9) | 100,864 (15.9) | 15,593 (15.9) | | 25 - 29 | 113,704 (15.5) | 98,500 (15.5) | 15,204 (15.5) | | 30 - 34 | 102,979 (14.1) | 89,095 (14.1) | 13,884 (14.1) | | 35 - 39 | 99,510 (13.6) | 86,222 (13.6) | 13,288 (13.5) | | 40 - 44 | 85,713 (11.7) | 74,220 (11.7) | 11,493 (11.7) | | 45 - 49 | 81,413 (11.1) | 70,657 (11.2) | 10,756 (10.9) | | Educational attainment, n (%) |  |  |  | | Primary school unfinished | 237,147 (32.4) | 219,028 (34.6) | 18,119 (18.4) | | Primary school finished | 48,798 (6.7) | 42,688 (6.7) | 6,110 ( 6.2) | | Secondary school unfinished | 294,749 (40.3) | 247,716 (39.1) | 47,033 (47.9) | | Secondary school finished or above | 151,170 (20.7) | 124,176 (19.6) | 26,994 (27.5) | | Household wealth quintile, n (%) |  |  |  | | Q1 (Poorest) | 135,076 (18.5) | 117,958 (18.6) | 17,118 (17.4) | | Q2 | 145,393 (19.9) | 126,221 (19.9) | 19,172 (19.5) | | Q3 | 150,958 (20.6) | 130,756 (20.6) | 20,202 (20.6) | | Q4 | 148,534 (20.3) | 127,965 (20.2) | 20,569 (20.9) | | Q5 (Richest) | 151,903 (20.8) | 130,708 (20.6) | 21,195 (21.6) | | BMI, n (%) |  |  |  | | <18.5kg/m^2^ | 159,909 (21.8) | 140,762 (22.2) | 19,147 (19.5) | | 18.5 - 22.9 kg/m^2^ | 341,033 (46.6) | 294,001 (46.4) | 47,032 (47.9) | | 23.0 - 24.9 kg/m^2^ | 95,585 (13.1) | 80,437 (12.7) | 15,148 (15.4) | | 25.0 - 27.4 kg/m^2^ | 68,964 (9.4) | 58,975 (9.3) | 9,989 (10.2) | | 27.5 - 29.9 kg/m^2^ | 35,225 (4.8) | 31,034 (4.9) | 4,191 (4.3) | | ≥ 30.0 kg/m^2^ | 30,111 (4.1) | 27,562 (4.4) | 2,549 (2.6) | | *Missing* | 1,037 (0.1) | 837 (0.1) | 200 (0.2) | | Tobacco consumption, n (%) |  |  |  | | Current smoker | 39,530 (5.4) | 13,216 (2.1) | 26,314 (26.8) | | Uses smokeless tobacco | 89,933 (12.3) | 57,887 (9.1) | 32,046 (32.6) | | Currently married, n (%) | 502,673 (68.7) | 443,407 (70.0) | 59,266 (60.3) | | Urban area, n (%) | 216,382 (29.6) | 185,538 (29.3) | 30,844 (31.4) |   Abbreviations: n=number; y=years; Q=quintile.  ^a^Sample characteristics were not weighted using sampling weights. |
|  |  | (b) Indicate number of participants with missing data for each variable of interest **[Results: “Sample characteristics” subsection, paragraph 1]** |
| Outcome data | 15* | Report numbers of outcome events or summary measures  **[Results: “The hypertension care cascade at the national level” subsection, paragraph 1]**  **The hypertension care cascade at the national level:**  The national prevalence of hypertension in the sampled age range was 18.1% (95% CI, 17.8% - 18.4%). Men had a somewhat higher prevalence than women (19.0%, 95% CI, 18.5% - 19.5%, compared to 17.2%, 95% CI, 16.9% - 17.4%). Hypertension prevalence estimates by age group and sex are shown in Table B, S1. Among hypertensive individuals, 76.1% (95% CI, 75.3% - 76.8%) had ever received a BP measurement (‘screened’), 44.7% (95% CI, 43.6% - 45.8%) had been diagnosed prior to the survey (‘aware’), 13.3% (95% CI, 12.9% - 13.8%) reported to be taking a prescribed antihypertensive drug (‘treated’), and 7.9% (95% CI, 7.6% - 8.3%) were on treatment and had a normal BP (‘controlled’). Women and participants living in urban areas were more likely reach each step of the care cascade (Fig 1). |
| Main results | 16 | (*a*) Give unadjusted estimates and, if applicable, confounder-adjusted estimates and their precision (eg, 95% confidence interval). Make clear which confounders were adjusted for and why they were included  **[Results: “The hypertension care cascade in relation to individuals’ characteristics” subsection, paragraph 1; Table 2]**  **The hypertension care cascade in relation to individuals’ characteristics**  Stratification of the hypertension cascade steps by age group, rural-urban residence, and household wealth quintile (Fig C, S1) and covariate-adjusted Poisson regressions (Table 2 and Table W, S1) show that i) women had a higher probability than men of completing each step of the cascade; ii) there was a positive association of ‘screened’ (in urban areas) and ‘treated’ with age group; iii) urban areas performed better than rural areas for all cascade steps; iv) being in a richer household wealth quintile was positively associated with completing each cascade step; v) education was positively associated with ‘screened’ and ‘aware’ but had no significant association with treatment and control; vi) being obese (BMI≥30.0kg/m^2^) was associated with a higher risk of reaching the ‘treated’ step and, in urban areas, also the ‘controlled’ step; and vii) tobacco consumption was generally not associated with substantial differences in the risk of progressing through the care cascade. In addition, those who were married had a higher risk of completing each care cascade step, with the association generally not differing between men and women (Table X, S1). The regression results were similar when using sampling weights (Table Y, S1), when run separately for men and women (Table Z - AA, S1), and when fitting covariate-unadjusted rather than covariate-adjusted regressions (Table AB - AE, S1). |
|  |  | (*b*) Report category boundaries when continuous variables were categorized **[not applicable]** |
|  |  | (*c*) If relevant, consider translating estimates of relative risk into absolute risk for a meaningful time period **[Table M and R]** |
| Other analyses | 17 | Report other analyses done—eg analyses of subgroups and interactions, and sensitivity analyses  **Table X - AF (S1) and Fig C (S1)** |
| Discussion | | |
| Key results | 18 | Summarise key results with reference to study objectives  **[Discussion: paragraph 1 - 3]**  **Discussion**  In this nationally representative sample of 731,864 adults aged 15-49 years in India, we found that while the proportion of those with hypertension who had ever had their BP measured was high (76%), less than half (45%) of individuals with the condition were aware of their diagnosis, less than one in seven (13%) reported to be taking BP-lowering medication, and less than one in 10 (8%) had achieved control. Thus, the highest absolute losses to care occurred at the awareness (31.4 percentage points) and treatment (31.4 percentage points) stage, and the highest relative loss at the treatment stage (70.3%). While improvements are needed along the entire hypertension care cascade, this highlights a particular need for interventions that focus on the awareness and treatment steps of the cascade.  The country-level analysis of the hypertension care cascade, however, disguises the large variation in the care cascades among states and population groups. Specifically, we found worse values for each of the care cascade steps for men, those in lower household wealth quintiles, and those living in rural areas. These populations thus form important target groups for appropriate interventions, particularly because households with less household wealth in rural areas are less likely to be able to access high-quality care for a CVD event, such as myocardial infarction, and more likely to experience catastrophic healthcare expenditures from such an event than their wealthier counterparts in urban areas.  Among states, we found that the proportion of adults with hypertension who were treated and controlled varied by a factor of 3.5 and 8.8, respectively. Chhattisgarh and Nagaland performed worse than expected based on GDP per capita on at least three out of four cascade steps. Identifying and adopting some of the policies and programs that have allowed equally wealthy (or even poorer) states to perform better may well prove effective in increasing health system performance for managing hypertension in poorly performing states such as Chhattisgarh and Nagaland. Such ‘role model’ states could include Jammu and Kashmir and Kerala, which performed better than predicted based on GDP per capita in at least three out of four care cascade steps. Other states where improvements in managing adults with hypertension are particularly urgent are those in which a large proportion of the general population had hypertension but did not reach the control step of the care cascade (“uncontrolled hypertension”), such as Puducherry (38.0%), Tamil Nadu (28.8%), Sikkim (28.4%), and Haryana (28.4%). However, India’s states vary enormously in size, and thus large states with relatively low hypertension prevalence may still host a high absolute number of adults with uncontrolled hypertension. In fact, Uttar Pradesh, which had one of the lowest prevalence levels for hypertension in the observed age group (14.4%, 95% CI, 13.9% - 14.9%), had the highest estimated absolute number of adults (14,267,516) with uncontrolled hypertension. Lastly, a priority state for improving hypertension care should be Tamil Nadu, which had both the second highest proportion of adults aged 15 to 49 years with uncontrolled hypertension (28.8%) as well as the second highest absolute number of adults in that age range with uncontrolled hypertension (12,820,905). |
| Limitations | 19 | Discuss limitations of the study, taking into account sources of potential bias or imprecision. Discuss both direction and magnitude of any potential bias  **[Discussion: paragraph 6]**  This study has several limitations. First, only adults aged 15-49 years were included in this analysis. Thus, our findings are not representative of the entire adult population in India. Nonetheless, given that India is a relatively young population, the age groups represented in this study accounted for 75.2% of all people in India aged ≥15 years in 2015.[39] The lower age of participants in this sample is also largely responsible for the lower hypertension prevalence observed in this study compared to the nationally representative study among an older sample that our team published recently.[5] Second, because of the NFHS-4’s focus on maternal and child health, the survey sampled substantially fewer men than women. However, with 98,256 men included in the analysis, the absolute number of men sampled was still sufficient to obtain reasonably precise estimates of hypertension care cascade indicators among men. In addition, we adjusted our sampling weights to the sex distribution of India’s population in each one-year age group to ensure that our estimates were representative for India’s adult population between the ages of 15 and 49 years despite the oversampling of women. Third, the definition of hypertension in this study was based on three BP measurements taken during one occasion, while a clinical diagnosis of hypertension requires raised BP measurements on at least two different occasions.[22,40] Falsely categorizing some adults as hypertensive who are normotensive would result in underestimates for ‘aware’, ‘treated’, and ‘controlled’. Fourth, 2.3% of participants had a missing value for at least one of the variables needed to define hypertension and each of the hypertension care cascade steps. While the percent missing is relatively small, those with a missing outcome variable are likely to have had a different probability of having hypertension and reaching each step in the care cascade than those included in the analysis as suggested by the fact that their socio-demographic characteristics were different (Table A, S1). Fifth, the questions asked in the NFHS-4 questionnaire did not allow us to ascertain who among those diagnosed with hypertension had received relevant lifestyle advice. Given that our construction of the hypertension care cascade imposed that a participant must have reached all previous cascade steps to reach the next cascade step, only those who were treated could achieve control. Thus, participants who were diagnosed with hypertension and subsequently achieved hypertension control through lifestyle changes rather than medication were not considered to have controlled hypertension in this study. Our analysis, therefore, likely underestimates the percent of those with hypertension who achieved control. Lastly, the question “Before this survey, has your blood pressure ever been checked?” that defined the outcome ‘screened’ does not quantify the number or regularity of BP measurements received prior to the survey. Our estimate of ‘screened’ should therefore be interpreted as the percentage who has ever had their BP measured (whether for hypertension screening or in other clinical interactions) rather than the percentage who has been screened recently or on a regular basis since reaching a certain age. |
| Interpretation | 20 | Give a cautious overall interpretation of results considering objectives, limitations, multiplicity of analyses, results from similar studies, and other relevant evidence  **[Discussion: paragraph 7]**  In conclusion, the proportion of adults with hypertension in India who are aware of their diagnosis, on treatment, or controlled is low. However, this study does not only set a benchmark for India as a whole to measure future progress. Rather, by providing a detailed analysis of how the hypertension care cascade varies among population groups and states, this study can inform target groups and the design of appropriate interventions to improve hypertension care. In particular, India needs to urgently improve hypertension control among households with lower levels of wealth and those living in rural areas, which will likely need to include access to low-cost or free anti-hypertensive medications. A further important target group is men. Since the Indian population forms 18% of the world’s population and is expected to be the world’s most populous country by 2025, India’s ability to improve hypertension care will have a decisive impact on the world’s ability to achieve international NCD goals, including the WHO’s Global Action Plan for the Prevention and Control of NCDs and the SDGs.[10,39,41] |
| Generalisability | 21 | Discuss the generalisability (external validity) of the study results  **[Discussion, paragraph 6]**  This study has several limitations. First, only adults aged 15-49 years were included in this analysis. Thus, our findings are not representative of the entire adult population in India. Nonetheless, given that India is a relatively young population, the age groups represented in this study accounted for 75.2% of all people in India aged ≥15 years in 2015.[39] The lower age of participants in this sample is also largely responsible for the lower hypertension prevalence observed in this study compared to the nationally representative study among an older sample that our team published recently.[5] Second, because of the NFHS-4’s focus on maternal and child health, the survey sampled substantially fewer men than women. However, with 98,256 men included in the analysis, the absolute number of men sampled was still sufficient to obtain reasonably precise estimates of hypertension care cascade indicators among men. In addition, we adjusted our sampling weights to the sex distribution of India’s population in each one-year age group to ensure that our estimates were representative for India’s adult population between the ages of 15 and 49 years despite the oversampling of women. Third, the definition of hypertension in this study was based on three BP measurements taken during one occasion, while a clinical diagnosis of hypertension requires raised BP measurements on at least two different occasions.[22,40] Falsely categorizing some adults as hypertensive who are normotensive would result in underestimates for ‘aware’, ‘treated’, and ‘controlled’. Fourth, 2.3% of participants had a missing value for at least one of the variables needed to define hypertension and each of the hypertension care cascade steps. While the percent missing is relatively small, those with a missing outcome variable are likely to have had a different probability of having hypertension and reaching each step in the care cascade than those included in the analysis as suggested by the fact that their socio-demographic characteristics were different (Table A, S1). Fifth, the questions asked in the NFHS-4 questionnaire did not allow us to ascertain who among those diagnosed with hypertension had received relevant lifestyle advice. Given that our construction of the hypertension care cascade imposed that a participant must have reached all previous cascade steps to reach the next cascade step, only those who were treated could achieve control. Thus, participants who were diagnosed with hypertension and subsequently achieved hypertension control through lifestyle changes rather than medication were not considered to have controlled hypertension in this study. Our analysis, therefore, likely underestimates the percent of those with hypertension who achieved control. Lastly, the question “Before this survey, has your blood pressure ever been checked?” that defined the outcome ‘screened’ does not quantify the number or regularity of BP measurements received prior to the survey. Our estimate of ‘screened’ should therefore be interpreted as the percentage who has ever had their BP measured (whether for hypertension screening or in other clinical interactions) rather than the percentage who has been screened recently or on a regular basis since reaching a certain age. |
| Other information | | |
| Funding | 22 | Give the source of funding and the role of the funders for the present study and, if applicable, for the original study on which the present article is based **[Footnotes: Funding subsection]**  **Funding:** Harvard McLennan Fund; Department of Science and Technology, Government of India, New Delhi, through the INSPIRE Faculty program  **Role of funding source:**  The funder had no role in the design of the study, analyses, interpretation of the data, or decision to submit for publication. |

*Give information separately for exposed and unexposed groups.

**Note:** An Explanation and Elaboration article discusses each checklist item and gives methodological background and published examples of transparent reporting. The STROBE checklist is best used in conjunction with this article (freely available on the Web sites of PLoS Medicine at http://www.plosmedicine.org/, Annals of Internal Medicine at http://www.annals.org/, and Epidemiology at http://www.epidem.com/). Information on the STROBE Initiative is available at www.strobe-statement.org.
