# Supplementary material for: Hypertension screening, awareness, treatment, and control in India: A nationally representative cross-sectional study among individuals aged 15 to 49 years
Source: PLoS Med. 2019 May 3;16(5):e1002801. doi: 10.1371/journal.pmed.1002801 (PMC6499417; doi:10.1371/journal.pmed.1002801)
Supplement: S1 Supplemental Materials — (DOCX) [file pmed.1002801.s002.docx]

**S1. Supplemental Materials**

[Methods A. Methodology of the NFHS-4 2](#_Toc4420915)

[Methods B. Computation of unmet need for care variables 5](#_Toc4420916)

[Methods C. Computation of the household wealth index 6](#_Toc4420917)

[Table A. Sample characteristics stratified by whether an outcome variable was missing^a,b,c^ 9](#_Toc4420918)

[Table B. Crude hypertension prevalence by five-year age group and sex 10](#_Toc4420919)

[Table C. Care cascade indicators by five-year age group 11](#_Toc4420920)

[Table D. State-level ‘screened’ estimates 12](#_Toc4420921)

[Table E. State-level ‘aware’ estimates 13](#_Toc4420922)

[Table F. State-level ‘treated’ estimates 14](#_Toc4420923)

[Table G. State-level ‘controlled’ estimates 15](#_Toc4420924)

[Table H. Crude hypertension prevalence estimates by state 16](#_Toc4420925)

[Table I. State-level prevalence estimates for ‘screened’ by sex 17](#_Toc4420926)

[Table J. State-level prevalence estimates for ‘aware’ by sex 19](#_Toc4420927)

[Table K. State-level prevalence estimates for ‘treated’ by sex 21](#_Toc4420928)

[Table L. State-level prevalence estimates for ‘controlled’ by sex 23](#_Toc4420929)

[Table M. Percentage of total adult population in each state with unmet need for care^a,b^ 25](#_Toc4420930)

[Table N. Confidence intervals for Table M ‘Unscreened’ estimates 27](#_Toc4420931)

[Table O. Confidence intervals for Table M ‘Unaware’ estimates 28](#_Toc4420932)

[Table P. Confidence intervals for Table M ‘Untreated’ estimates 29](#_Toc4420933)

[Table Q. Confidence intervals for Table M ‘Uncontrolled’ estimates 30](#_Toc4420934)

[Table R. Absolute number of total adult population in each state with unmet need for care^a,b^ 31](#_Toc4420935)

[Table S. Confidence intervals for Table R ‘Unscreened’ estimates 33](#_Toc4420936)

[Table T. Confidence intervals for Table R ‘Unaware’ estimates 34](#_Toc4420937)

[Table U. Confidence intervals for Table R ‘Untreated’ estimates 35](#_Toc4420938)

[Table V. Confidence intervals for Table R ‘Uncontrolled’ estimates 36](#_Toc4420939)

[Table W. Predictors of ‘screened’ and ‘aware’ among participants with hypertension^a^ 37](#_Toc4420940)

[Table X. Predictors of reaching each cascade step using covariate-adjusted poisson regressions with an interaction term between marital status and sex^a^ 40](#_Toc4420941)

[Table Y. Predictors of each cascade step using covariate-adjusted poisson regressions with sampling weights^a^ 46](#_Toc4420942)

[Table Z. Predictors of reaching each cascade step using covariate-adjusted poisson regressions among men only^a^ 52](#_Toc4420943)

[Table AA. Predictors of reaching each cascade step using covariate-adjusted poisson regressions among women only^a^ 58](#_Toc4420944)

[Table AB. Predictors of reaching each cascade step using covariate-unadjusted poisson regressions^a^ 65](#_Toc4420945)

[Table AC. Predictors of reaching each cascade step using covariate-unadjusted poisson regressions with sampling weights^a^ 71](#_Toc4420946)

[Table AD. Predictors of reaching each cascade step using covariate-unadjusted poisson regressions among women only^a^ 78](#_Toc4420947)

[Table AE. Predictors of reaching each cascade step using covariate-unadjusted poisson regressions among men only^a^ 84](#_Toc4420948)

[Table AF. Predictors of reaching each cascade step using covariate-adjusted poisson regressions with restricted cubic splines of five knots for the continuous variables age, wealth index and BMI^a^ 90](#_Toc4420949)

[Fig A. Flowchart of patients lost in hypertension care (using sampling weights) 94](#_Toc4420950)

[Fig B. Map of crude hypertension prevalence by state and sex^a,b^ 95](#_Toc4420951)

[Fig C. Percent that reached each cascade step by rural-urban residence, sex, age group, and household wealth quintile 96](#_Toc4420952)

# Methods A. Methodology of the NFHS-4

The NFHS-4 was designed to yield precise health indicators at multiple geographic levels (district, state/union territory, national) and for different areas (urban/rural, slum/non-slum areas in large cities). A stratified two-stage random sampling design was employed. Primary sampling units (PSUs) were defined as villages in rural areas and Census Enumeration Blocks (CEBs) in urban areas according to the 2011 Census for India. Small PSUs (fewer than 40 households) were added to the nearest PSU. Strata were rural and urban areas within each of India’s 640 districts. Villages in each rural stratum and CEBs in each urban stratum were selected with probability proportional to population size (PPS). In rural areas, six substrata within each stratum were created by crossing three substrata (defined based on the estimated number of households in each village) with two substrata (defined based on the percentage of the population belonging to scheduled castes and tribes). PSUs with more than 300 estimated households were divided into segments of 100-150 households, and two of these segments were randomly selected (again, using PPS) for the survey. In each of these clusters (PSUs or PSU segments), 22 households were randomly selected using systematic random sampling.

Four Computer Assisted Personal Interviewing (CAPI) questionnaires (Household Questionnaire, Woman’s Questionnaire, Man’s Questionnaire, and Biomarker Questionnaire) were administered in the local language by trained interviewers. The training procedure has been described in the main text.

In the Household Questionnaire, all usual members of the household and visitors who stayed in the household the night before the interview were listed and written consent for the survey was obtained from each individual. Basic demographic information including age, sex, marital status, schooling, relationship to the head of the household was collected for each listed person.

Personal interviews using the Woman's and Man's Questionnaire as well as physical measurements using the Biomarker Questionnaire were only conducted among listed persons of age 15-49 years (Women) or 15-54 years (Men). The Woman's Questionnaire, Man's Questionnaire and the Biomarker Questionnaire contained the key measures used in this study. More detailed information on the sampling procedure, the questionnaire items and the execution of the survey can be found in the formal report of the NFHS-4, the published questionnaires and the multiple manuals that were distributed to the staff, which are all available online.[1-8]

# Methods B. Computation of unmet need for care variables

For Table M and Table R, the hypertension cascade of care indicators were converted into unmet need for care population percentages (Table M), as well as total number estimates of unmet need for care of each state and Union Territory (Table R). The following steps were performed to calculate these estimates: i) the reciprocal value of each care cascade indicator (Screened, Aware, Treated, and Controlled) was calculated to obtain unmet need for care estimates (Unscreened, Unaware, Untreated, Uncontrolled); ii) the new unmet need for care values were multiplied with the hypertension prevalence in each state/Union Territory to calculate the percentage of all adults in the relevant age range (15-49 years) in a state/Union Territory who had an unmet need for hypertension care (Table M); and iii) the estimate from step ii) was multiplied with the population in a State/Union Territory in the relevant age range to obtain the absolute number of adults who were unscreened, unaware, untreated and uncontrolled (Table R). Estimates of the population size of each state and Union Territory were obtained (for each one year age group) from the 2011 national census.[9]

# Methods C. Computation of the household wealth index

A detailed step-by-step description of the calculation of the household wealth index in the NFHS-4 survey has been published elsewhere.[6, 10] In brief, the household wealth index in the NFHS-4 was constructed based on the following measures of household characteristics:

- Main roof material
- Main wall material
- Main material of floor
- Type of cooking fuel
- Type of toilet facility
- Source of drinking water

In addition, the ownership of the following durable goods (‘assets’) was used:

| - Mattress |
| --- |
| - Pressure cooker |
| - Chair |
| - Cot or bed |
| - Table |
| - Electric fan |
| - Radio or transistor |
| - Black and white television |
| - Colour television |
| - Sewing machine |
| - Mobile telephone |
| - Telephone (non-mobile) |
| - Internet |
| - Computer |
| - Refrigerator |
| - Air conditioner/cooler |
| - Washing machine |
| - Watch or clock |
| - Bicycle |
| - Motorcycle or Scooter |
| - Animal-drawn cart |
| - Car |
| - Water pump |
| - Thresher |
| - Tractor |

Each household characteristic and asset was then encoded as a binary indicator, which were then summarized in a Principal Component Analysis (PCA). The first (unrotated) component was extracted separately for rural and urban areas. This resulted in a standardized score with a mean of zero, a standard deviation of one, and higher scores for wealthier households. This continuous variable was then divided into quintiles for easier interpretability.

# Table A. Sample characteristics stratified by whether an outcome variable was missing^1,2,3^

| Characteristic | Has a missing outcome variable | Does not have a missing outcome variable |
| --- | --- | --- |
| No. | 17,255 | 731,864 |
| Male, n (%) | 3,412 ( 19.8) | 98,256 (13.4) |
| Age Group, n (%), y |  |  |
| 15-19 | 3,881 (22.5) | 132,088 (18.0) |
| 20-24 | 2,874 (16.7) | 116,457 (15.9) |
| 25-29 | 2,627 (15.2) | 113,704 (15.5) |
| 30-34 | 2,224 (12.9) | 102,979 (14.1) |
| 35-39 | 2,059 (11.9) | 99,510 (13.6) |
| 40-44 | 1,806 (10.5) | 85,713 (11.7) |
| 45-49 | 1,784 (10.3) | 81,413 (11.1) |
| Education, n (%) |  |  |
| Primary school unfinished | 4,591 (26.6) | 237,147 (32.4) |
| Primary school finished | 901 ( 5.2) | 48,798 ( 6.7) |
| Secondary school unfinished | 6,949 (40.3) | 294,749 (40.3) |
| Secondary school finished or above | 4,814 (27.9) | 151,170 (20.7) |
| Household wealth quintile, n (%) |  |  |
| Q1 (Poorest) | 2,904 (16.8) | 135,076 (18.5) |
| Q2 | 3,125 (18.1) | 145,393 (19.9) |
| Q3 | 3,345 (19.4) | 150,958 (20.6) |
| Q4 | 3,513 (20.4) | 148,534 (20.3) |
| Q5 (Richest) | 4,368 (25.3) | 151,903 (20.8) |
| BMI, n (%) |  |  |
| <18.5kg/m2 | 1,013 ( 5.9) | 159,909 (21.8) |
| 18.5-22.9 kg/m2 | 1,997 (11.6) | 341,033 (46.6) |
| 23.0-24.9 kg/m2 | 491 ( 2.8) | 95,585 (13.1) |
| 25.0-27.4 kg/m2 | 320 ( 1.9) | 68,964 ( 9.4) |
| 27.5-29.9 kg/m2 | 156 ( 0.9) | 35,225 ( 4.8) |
| >= 30.0 kg/m2 | 146 ( 0.8) | 30,111 ( 4.1) |
| Missing^c^ | 13,132 (76.1) | 1,037 ( 0.1) |
| Tobacco consumption, n (%) |  |  |
| Tobacco consume, smoked | 994 ( 5.8) | 39,530 ( 5.4) |
| Tobacco consume, smokeless | 1,765 (10.2) | 89,933 (12.3) |
| Currently married, n (%) | 10,084 (58.4) | 502,673 (68.7) |
| Urban area, n (%) | 7,433 (43.1) | 216,382 (29.6) |

Abbreviations: No.=number; %=Percentage.

^1^ These numbers were not weighted using sampling weights.

^2^ The outcome variables were blood pressure and each of the questions used to ascertain whether a participant had reached each cascade step.

^3^ None of the other variables shown besides BMI had missing values.

# Table B. Crude hypertension prevalence by five-year age group and sex

| Age group (years) | Total, % (95% CI) | Women, % (95% CI) | Men, % (95% CI) |
| --- | --- | --- | --- |
| 15-19 | 4.8 (4.5-5.1) | 4.3 (4.2-4.5) | 5.2 (4.8-5.7) |
| 20-24 | 9.9 (9.5-10.3) | 9.6 (9.2-9.9) | 10.2 (9.5-11.0) |
| 25-29 | 14.9 (14.3-15.5) | 13.9 (13.5-14.4) | 15.8 (14.8-16.9) |
| 30-34 | 19.4 (18.8-20.0) | 17.6 (17.2-18.1) | 21.2 (20.1-22.3) |
| 35-39 | 24.4 (23.7-25.1) | 22.4 (21.9-22.8) | 26.4 (25.1-27.7) |
| 40-44 | 29.4 (28.7-30.2) | 27.8 (27.3-28.3) | 31.0 (29.6-32.4) |
| 45-49 | 34.0 (33.2-34.8) | 33.6 (33.0-34.2) | 34.4 (33.0-35.8) |

# Table C. Care cascade indicators by five-year age group

*Screened:*

| Age group | Screened, in % | Lower 95% CI | Upper 95% CI |
| --- | --- | --- | --- |
| 15-19 | 62.25 | 59.32 | 65.10 |
| 20-24 | 73.37 | 71.43 | 75.23 |
| 25-29 | 77.73 | 76.05 | 79.33 |
| 30-34 | 75.28 | 73.59 | 76.91 |
| 35-39 | 76.33 | 74.78 | 77.82 |
| 40-44 | 77.32 | 75.94 | 78.65 |
| 45-49 | 78.48 | 77.27 | 79.65 |

*Awareness:*

| Age group | Aware, in % | Lower 95% CI | Upper 95% CI |
| --- | --- | --- | --- |
| 15-19 | 50.15 | 47.13 | 53.17 |
| 20-24 | 52.29 | 49.99 | 54.57 |
| 25-29 | 48.45 | 46.28 | 50.63 |
| 30-34 | 43.54 | 41.74 | 45.36 |
| 35-39 | 41.21 | 39.52 | 42.93 |
| 40-44 | 41.52 | 39.93 | 43.12 |
| 45-49 | 44.64 | 43.11 | 46.17 |

*Treatment:*

| Age group | Treated, in % | Lower 95% CI | Upper 95% CI |
| --- | --- | --- | --- |
| 15-19 | 10.75 | 9.11 | 12.64 |
| 20-24 | 8.97 | 7.96 | 10.09 |
| 25-29 | 9.14 | 8.32 | 10.02 |
| 30-34 | 10.15 | 9.36 | 11.00 |
| 35-39 | 11.35 | 10.53 | 12.22 |
| 40-44 | 16.29 | 15.11 | 17.54 |
| 45-49 | 19.28 | 18.25 | 20.35 |

*Control:*

| Age group | Controlled, in % | Lower 95% CI | Upper 95% CI |
| --- | --- | --- | --- |
| 15-19 | 10.32 | 8.70 | 12.21 |
| 20-24 | 7.95 | 7.00 | 9.02 |
| 25-29 | 7.39 | 6.68 | 8.17 |
| 30-34 | 6.97 | 6.36 | 7.63 |
| 35-39 | 6.43 | 5.88 | 7.02 |
| 40-44 | 8.12 | 7.20 | 9.14 |
| 45-49 | 9.38 | 8.63 | 10.19 |

# Table D. State-level ‘screened’ estimates

| State | Estimate | Lower 95% CI | Upper 95% CI |
| --- | --- | --- | --- |
| Andaman and Nicobar Islands | 77.4 | 68.3 | 84.4 |
| Andhra Pradesh | 75.3 | 71.1 | 79.1 |
| Arunachal Pradesh | 69.3 | 65.4 | 72.9 |
| Assam | 73.9 | 71.5 | 76.1 |
| Bihar | 70.0 | 67.0 | 72.9 |
| Chandigarh | 93.1 | 82.1 | 97.5 |
| Chhattisgarh | 66.8 | 63.2 | 70.2 |
| Dadra and Nagar Haveli | 78.8 | 61.9 | 89.5 |
| Daman and Diu | 62.6 | 44.1 | 78.0 |
| Delhi | 87.3 | 79.6 | 92.4 |
| Goa | 87.3 | 82.0 | 91.2 |
| Gujarat | 66.8 | 63.4 | 70.0 |
| Haryana | 93.5 | 91.9 | 94.8 |
| Himachal Pradesh | 71.7 | 67.7 | 75.4 |
| Jammu and Kashmir | 82.1 | 79.7 | 84.4 |
| Jharkhand | 74.2 | 71.4 | 76.9 |
| Karnataka | 74.8 | 71.4 | 77.8 |
| Kerala | 89.0 | 85.3 | 91.9 |
| Lakshadweep | 83.7 | 71.0 | 91.5 |
| Madhya Pradesh | 61.3 | 59.1 | 63.3 |
| Maharashtra | 70.9 | 66.8 | 74.8 |
| Manipur | 88.7 | 85.9 | 91.1 |
| Meghalaya | 73.2 | 68.9 | 77.1 |
| Mizoram | 87.2 | 80.7 | 91.7 |
| Nagaland | 78.7 | 75.3 | 81.8 |
| Odisha | 66.7 | 63.9 | 69.3 |
| Puducherry | 91.9 | 86.2 | 95.3 |
| Punjab | 91.5 | 89.3 | 93.3 |
| Rajasthan | 71.2 | 68.6 | 73.6 |
| Sikkim | 85.7 | 82.0 | 88.7 |
| Tamil Nadu | 86.6 | 84.7 | 88.2 |
| Telangana | 80.5 | 76.4 | 84.0 |
| Tripura | 74.2 | 67.5 | 79.9 |
| Uttar Pradesh | 68.7 | 66.9 | 70.4 |
| Uttarakhand | 74.2 | 70.4 | 77.6 |
| West Bengal | 81.5 | 78.4 | 84.3 |

# Table E. State-level ‘aware’ estimates

| State | Estimate | Lower 95% CI | Upper 95% CI |
| --- | --- | --- | --- |
| Andaman and Nicobar Islands | 39.1 | 29.4 | 49.6 |
| Andhra Pradesh | 44.0 | 38.7 | 49.5 |
| Arunachal Pradesh | 34.8 | 31.0 | 38.9 |
| Assam | 33.0 | 30.1 | 35.9 |
| Bihar | 46.8 | 43.3 | 50.4 |
| Chandigarh | 55.2 | 39.6 | 69.9 |
| Chhattisgarh | 22.1 | 19.6 | 24.9 |
| Dadra and Nagar Haveli | 26.7 | 15.9 | 41.2 |
| Daman and Diu | 43.6 | 27.0 | 61.8 |
| Delhi | 61.6 | 50.3 | 71.8 |
| Goa | 28.8 | 21.6 | 37.2 |
| Gujarat | 26.3 | 23.4 | 29.4 |
| Haryana | 67.4 | 62.9 | 71.5 |
| Himachal Pradesh | 36.3 | 32.8 | 40.0 |
| Jammu and Kashmir | 50.7 | 47.2 | 54.1 |
| Jharkhand | 49.3 | 45.5 | 53.2 |
| Karnataka | 30.6 | 26.9 | 34.6 |
| Kerala | 46.3 | 42.1 | 50.6 |
| Lakshadweep | 28.5 | 19.8 | 39.1 |
| Madhya Pradesh | 29.8 | 28.0 | 31.6 |
| Maharashtra | 34.1 | 30.2 | 38.2 |
| Manipur | 41.4 | 37.5 | 45.4 |
| Meghalaya | 47.9 | 43.1 | 52.8 |
| Mizoram | 52.7 | 47.7 | 57.7 |
| Nagaland | 33.9 | 30.9 | 37.2 |
| Odisha | 47.6 | 44.3 | 51.0 |
| Puducherry | 80.5 | 69.8 | 88.1 |
| Punjab | 39.6 | 36.2 | 43.0 |
| Rajasthan | 37.1 | 34.4 | 39.9 |
| Sikkim | 48.6 | 44.4 | 52.8 |
| Tamil Nadu | 70.0 | 66.5 | 73.3 |
| Telangana | 55.3 | 50.1 | 60.4 |
| Tripura | 40.4 | 34.3 | 46.7 |
| Uttar Pradesh | 42.0 | 40.0 | 44.1 |
| Uttarakhand | 37.8 | 33.7 | 42.1 |
| West Bengal | 39.0 | 35.5 | 42.6 |

# Table F. State-level ‘treated’ estimates

| State | Estimate | Lower 95% CI | Upper 95% CI |
| --- | --- | --- | --- |
| Andaman and Nicobar Islands | 22.1 | 15.9 | 29.8 |
| Andhra Pradesh | 14.2 | 11.9 | 16.9 |
| Arunachal Pradesh | 10.3 | 8.6 | 12.2 |
| Assam | 12.0 | 10.9 | 13.3 |
| Bihar | 17.3 | 15.4 | 19.3 |
| Chandigarh | 18.0 | 9.2 | 32.3 |
| Chhattisgarh | 8.6 | 7.2 | 10.3 |
| Dadra and Nagar Haveli | 17.0 | 8.4 | 31.3 |
| Daman and Diu | 23.9 | 12.3 | 41.3 |
| Delhi | 17.0 | 11.5 | 24.5 |
| Goa | 18.1 | 14.0 | 23.1 |
| Gujarat | 11.6 | 9.8 | 13.8 |
| Haryana | 14.2 | 12.1 | 16.5 |
| Himachal Pradesh | 10.9 | 9.0 | 13.0 |
| Jammu and Kashmir | 19.9 | 18.0 | 21.9 |
| Jharkhand | 7.1 | 5.9 | 8.5 |
| Karnataka | 13.2 | 11.4 | 15.2 |
| Kerala | 21.5 | 18.3 | 25.1 |
| Lakshadweep | 14.3 | 9.0 | 21.8 |
| Madhya Pradesh | 14.4 | 13.1 | 15.9 |
| Maharashtra | 14.2 | 12.2 | 16.6 |
| Manipur | 8.2 | 6.8 | 9.7 |
| Meghalaya | 24.7 | 20.8 | 29.1 |
| Mizoram | 11.3 | 9.1 | 14.0 |
| Nagaland | 7.5 | 6.0 | 9.2 |
| Odisha | 15.9 | 14.2 | 17.7 |
| Puducherry | 11.0 | 6.5 | 18.3 |
| Punjab | 11.8 | 10.3 | 13.6 |
| Rajasthan | 11.1 | 9.9 | 12.5 |
| Sikkim | 17.4 | 14.2 | 21.2 |
| Tamil Nadu | 12.7 | 11.3 | 14.2 |
| Telangana | 16.4 | 13.8 | 19.4 |
| Tripura | 17.6 | 13.9 | 22.1 |
| Uttar Pradesh | 9.6 | 8.8 | 10.5 |
| Uttarakhand | 12.8 | 10.9 | 15.0 |
| West Bengal | 14.7 | 12.6 | 17.0 |

# Table G. State-level ‘controlled’ estimates

| State | Estimate | Lower 95% CI | Upper 95% CI |
| --- | --- | --- | --- |
| Andaman and Nicobar Islands | 6.5 | 4.2 | 10.0 |
| Andhra Pradesh | 7.9 | 6.3 | 9.8 |
| Arunachal Pradesh | 4.7 | 3.6 | 5.9 |
| Assam | 5.0 | 4.3 | 5.9 |
| Bihar | 12.3 | 10.8 | 14.1 |
| Chandigarh | 6.0 | 2.6 | 13.2 |
| Chhattisgarh | 4.4 | 3.6 | 5.4 |
| Dadra and Nagar Haveli | 7.2 | 3.0 | 16.2 |
| Daman and Diu | 21.0 | 9.8 | 39.6 |
| Delhi | 12.1 | 7.2 | 19.5 |
| Goa | 11.8 | 8.4 | 16.3 |
| Gujarat | 7.5 | 6.2 | 9.1 |
| Haryana | 9.3 | 7.7 | 11.3 |
| Himachal Pradesh | 5.6 | 4.5 | 7.0 |
| Jammu and Kashmir | 11.9 | 10.5 | 13.5 |
| Jharkhand | 4.5 | 3.7 | 5.4 |
| Karnataka | 7.3 | 5.8 | 9.3 |
| Kerala | 15.4 | 12.6 | 18.8 |
| Lakshadweep | 8.9 | 4.8 | 15.9 |
| Madhya Pradesh | 9.3 | 8.2 | 10.4 |
| Maharashtra | 9.4 | 7.6 | 11.5 |
| Manipur | 3.4 | 2.6 | 4.4 |
| Meghalaya | 18.2 | 14.6 | 22.5 |
| Mizoram | 5.9 | 4.5 | 7.8 |
| Nagaland | 2.4 | 1.7 | 3.3 |
| Odisha | 10.9 | 9.6 | 12.5 |
| Puducherry | 5.0 | 2.6 | 9.2 |
| Punjab | 4.6 | 3.8 | 5.4 |
| Rajasthan | 6.8 | 5.8 | 7.8 |
| Sikkim | 7.3 | 5.5 | 9.7 |
| Tamil Nadu | 7.0 | 6.0 | 8.2 |
| Telangana | 9.4 | 7.4 | 11.7 |
| Tripura | 10.9 | 7.9 | 14.9 |
| Uttar Pradesh | 6.6 | 6.0 | 7.3 |
| Uttarakhand | 7.7 | 6.3 | 9.4 |
| West Bengal | 5.4 | 4.3 | 6.6 |

# Table H. Crude hypertension prevalence estimates by state

| State/Union Territory | Estimate, in % | Lower 95% CI | Upper 95% CI |
| --- | --- | --- | --- |
| Andaman and Nicobar Islands | 22.4 | 19.2 | 26.0 |
| Andhra Pradesh | 20.5 | 18.6 | 22.6 |
| Arunachal Pradesh | 24.6 | 23.0 | 26.4 |
| Assam | 24.6 | 23.6 | 25.6 |
| Bihar | 12.8 | 12.0 | 13.7 |
| Chandigarh | 20.7 | 16.2 | 26.1 |
| Chhattisgarh | 14.0 | 13.1 | 15.0 |
| Dadra and Nagar Haveli | 14.4 | 10.6 | 19.3 |
| Daman and Diu | 13.4 | 10.1 | 17.5 |
| Delhi | 13.7 | 11.0 | 16.8 |
| Goa | 15.5 | 13.0 | 18.5 |
| Gujarat | 15.8 | 14.8 | 16.8 |
| Haryana | 31.3 | 28.8 | 34.0 |
| Himachal Pradesh | 24.3 | 22.7 | 25.9 |
| Jammu and Kashmir | 21.0 | 19.8 | 22.3 |
| Jharkhand | 17.9 | 16.8 | 19.1 |
| Karnataka | 17.3 | 16.0 | 18.6 |
| Kerala | 13.0 | 11.9 | 14.3 |
| Lakshadweep | 15.5 | 12.3 | 19.3 |
| Madhya Pradesh | 13.0 | 12.5 | 13.6 |
| Maharashtra | 17.9 | 16.8 | 19.1 |
| Manipur | 22.9 | 21.5 | 24.4 |
| Meghalaya | 17.6 | 16.0 | 19.4 |
| Mizoram | 22.9 | 20.6 | 25.4 |
| Nagaland | 23.7 | 22.2 | 25.4 |
| Odisha | 18.2 | 17.2 | 19.3 |
| Puducherry | 40.0 | 32.4 | 48.1 |
| Punjab | 24.0 | 22.7 | 25.3 |
| Rajasthan | 14.6 | 13.9 | 15.4 |
| Sikkim | 30.7 | 28.1 | 33.4 |
| Tamil Nadu | 31.0 | 29.0 | 33.1 |
| Telangana | 23.6 | 21.7 | 25.6 |
| Tripura | 20.2 | 18.2 | 22.4 |
| Uttar Pradesh | 14.4 | 13.9 | 14.9 |
| Uttarakhand | 18.6 | 17.3 | 19.9 |
| West Bengal | 15.9 | 14.8 | 17.1 |

# Table I. State-level prevalence estimates for ‘screened’ by sex

| Sex | State | Estimate | Lower 95% CI | Upper 95% CI |
| --- | --- | --- | --- | --- |
| Female | Andaman and Nicobar Islands | 80.9 | 74.6 | 85.9 |
| Female | Andhra Pradesh | 82.7 | 79.9 | 85.1 |
| Female | Arunachal Pradesh | 74.4 | 71.7 | 76.9 |
| Female | Assam | 80.6 | 79.0 | 82.0 |
| Female | Bihar | 77.9 | 76.2 | 79.6 |
| Female | Chandigarh | 99.1 | 93.9 | 99.9 |
| Female | Chhattisgarh | 74.7 | 72.1 | 77.2 |
| Female | Dadra and Nagar Haveli | 77.9 | 65.6 | 86.7 |
| Female | Daman and Diu | 69.0 | 58.7 | 77.8 |
| Female | Goa | 91.4 | 86.6 | 94.5 |
| Female | Gujarat | 96.3 | 92.5 | 98.2 |
| Female | Haryana | 76.4 | 74.0 | 78.7 |
| Female | Himachal Pradesh | 97.2 | 96.6 | 97.7 |
| Female | Jammu and Kashmir | 86.2 | 83.7 | 88.4 |
| Female | Jharkhand | 91.7 | 90.5 | 92.8 |
| Female | Karnataka | 79.9 | 78.0 | 81.7 |
| Female | Kerala | 82.7 | 80.8 | 84.5 |
| Female | Lakshadweep | 93.3 | 91.2 | 94.9 |
| Female | Madhya Pradesh | 86.5 | 79.4 | 91.5 |
| Female | Maharashtra | 71.7 | 70.2 | 73.2 |
| Female | Manipur | 83.9 | 81.8 | 85.9 |
| Female | Meghalaya | 94.1 | 92.9 | 95.1 |
| Female | Mizoram | 78.9 | 74.5 | 82.7 |
| Female | Nagaland | 92.4 | 90.0 | 94.3 |
| Female | Delhi | 80.7 | 78.2 | 83.1 |
| Female | Odisha | 74.8 | 72.8 | 76.8 |
| Female | Puducherry | 93.5 | 88.9 | 96.2 |
| Female | Punjab | 96.5 | 95.6 | 97.2 |
| Female | Rajasthan | 83.2 | 81.7 | 84.6 |
| Female | Sikkim | 91.5 | 89.1 | 93.4 |
| Female | Tamil Nadu | 93.3 | 92.3 | 94.2 |
| Female | Telangana | 87.4 | 85.0 | 89.4 |
| Female | Tripura | 83.2 | 79.6 | 86.3 |
| Female | Uttar Pradesh | 78.4 | 77.2 | 79.4 |
| Female | Uttarakhand | 84.9 | 83.0 | 86.5 |
| Female | West Bengal | 87.6 | 86.0 | 89.2 |
| Male | Andaman and Nicobar Islands | 75.5 | 61.9 | 85.3 |
| Male | Andhra Pradesh | 68.9 | 61.6 | 75.2 |
| Male | Arunachal Pradesh | 65.0 | 58.7 | 70.8 |
| Male | Assam | 67.6 | 63.4 | 71.5 |
| Male | Bihar | 61.5 | 55.7 | 67.1 |
| Male | Chandigarh | 89.5 | 70.9 | 96.7 |
| Male | Chhattisgarh | 59.9 | 54.0 | 65.5 |
| Male | Dadra and Nagar Haveli | 79.2 | 54.9 | 92.3 |
| Male | Daman and Diu | 60.8 | 37.6 | 80.0 |
| Male | Goa | 82.2 | 64.0 | 92.3 |
| Male | Gujarat | 84.9 | 78.3 | 89.7 |
| Male | Haryana | 61.8 | 56.9 | 66.4 |
| Male | Himachal Pradesh | 90.5 | 87.6 | 92.7 |
| Male | Jammu and Kashmir | 64.6 | 59.1 | 69.8 |
| Male | Jharkhand | 74.9 | 70.9 | 78.6 |
| Male | Karnataka | 68.8 | 63.9 | 73.2 |
| Male | Kerala | 68.6 | 62.8 | 73.8 |
| Male | Lakshadweep | 85.8 | 79.4 | 90.4 |
| Male | Madhya Pradesh | 79.6 | 52.7 | 93.2 |
| Male | Maharashtra | 51.8 | 48.1 | 55.3 |
| Male | Manipur | 61.6 | 54.9 | 67.8 |
| Male | Meghalaya | 84.0 | 79.0 | 88.1 |
| Male | Mizoram | 65.7 | 57.7 | 73.0 |
| Male | Nagaland | 81.9 | 69.5 | 90.0 |
| Male | Delhi | 76.9 | 71.1 | 81.8 |
| Male | Odisha | 56.5 | 51.5 | 61.5 |
| Male | Puducherry | 90.6 | 80.8 | 95.6 |
| Male | Punjab | 87.9 | 84.5 | 90.7 |
| Male | Rajasthan | 61.6 | 57.4 | 65.6 |
| Male | Sikkim | 81.5 | 75.2 | 86.5 |
| Male | Tamil Nadu | 80.7 | 77.5 | 83.5 |
| Male | Telangana | 74.2 | 67.0 | 80.3 |
| Male | Tripura | 65.8 | 55.1 | 75.1 |
| Male | Uttar Pradesh | 58.0 | 54.8 | 61.1 |
| Male | Uttarakhand | 64.6 | 57.9 | 70.7 |
| Male | West Bengal | 75.1 | 68.9 | 80.5 |

# Table J. State-level prevalence estimates for ‘aware’ by sex

| Sex | State | Estimate | Lower 95% CI | Upper 95% CI |
| --- | --- | --- | --- | --- |
| Female | Andaman and Nicobar Islands | 55.8 | 46.6 | 64.6 |
| Female | Andhra Pradesh | 55.4 | 51.3 | 59.4 |
| Female | Arunachal Pradesh | 42.5 | 39.1 | 46.0 |
| Female | Assam | 39.7 | 37.5 | 41.9 |
| Female | Bihar | 55.2 | 52.7 | 57.7 |
| Female | Chandigarh | 62.5 | 50.5 | 73.1 |
| Female | Chhattisgarh | 31.3 | 28.5 | 34.2 |
| Female | Dadra and Nagar Haveli | 40.4 | 29.8 | 52.0 |
| Female | Daman and Diu | 43.2 | 31.2 | 56.0 |
| Female | Goa | 62.2 | 54.6 | 69.1 |
| Female | Gujarat | 45.7 | 36.5 | 55.2 |
| Female | Haryana | 37.4 | 34.5 | 40.4 |
| Female | Himachal Pradesh | 78.3 | 75.5 | 80.8 |
| Female | Jammu and Kashmir | 54.3 | 50.6 | 58.0 |
| Female | Jharkhand | 67.4 | 64.9 | 69.9 |
| Female | Karnataka | 57.5 | 54.6 | 60.4 |
| Female | Kerala | 42.3 | 39.0 | 45.6 |
| Female | Lakshadweep | 53.8 | 50.4 | 57.2 |
| Female | Madhya Pradesh | 42.1 | 34.7 | 49.9 |
| Female | Maharashtra | 40.6 | 39.0 | 42.3 |
| Female | Manipur | 47.1 | 43.9 | 50.3 |
| Female | Meghalaya | 53.3 | 49.6 | 56.9 |
| Female | Mizoram | 57.5 | 53.2 | 61.7 |
| Female | Nagaland | 68.3 | 64.6 | 71.8 |
| Female | Delhi | 40.2 | 37.5 | 43.0 |
| Female | Odisha | 57.7 | 55.1 | 60.2 |
| Female | Puducherry | 84.3 | 76.5 | 89.8 |
| Female | Punjab | 55.4 | 52.8 | 58.0 |
| Female | Rajasthan | 51.0 | 48.9 | 53.1 |
| Female | Sikkim | 57.9 | 54.5 | 61.3 |
| Female | Tamil Nadu | 80.0 | 77.7 | 82.2 |
| Female | Telangana | 68.6 | 64.6 | 72.4 |
| Female | Tripura | 47.9 | 43.2 | 52.7 |
| Female | Uttar Pradesh | 53.0 | 51.4 | 54.6 |
| Female | Uttarakhand | 53.4 | 50.2 | 56.7 |
| Female | West Bengal | 44.5 | 41.6 | 47.5 |
| Male | Andaman and Nicobar Islands | 30.0 | 17.3 | 46.8 |
| Male | Andhra Pradesh | 34.0 | 26.0 | 43.0 |
| Male | Arunachal Pradesh | 28.5 | 23.0 | 34.6 |
| Male | Assam | 26.7 | 22.2 | 31.8 |
| Male | Bihar | 37.8 | 31.7 | 44.3 |
| Male | Chandigarh | 50.9 | 27.2 | 74.2 |
| Male | Chhattisgarh | 14.2 | 10.9 | 18.2 |
| Male | Dadra and Nagar Haveli | 21.0 | 8.8 | 42.5 |
| Male | Daman and Diu | 43.7 | 23.4 | 66.4 |
| Male | Goa | 61.0 | 39.3 | 79.0 |
| Male | Gujarat | 24.3 | 16.7 | 34.0 |
| Male | Haryana | 20.5 | 16.9 | 24.7 |
| Male | Himachal Pradesh | 58.5 | 51.6 | 65.1 |
| Male | Jammu and Kashmir | 27.6 | 23.4 | 32.2 |
| Male | Jharkhand | 38.0 | 33.5 | 42.7 |
| Male | Karnataka | 41.5 | 35.4 | 47.8 |
| Male | Kerala | 21.5 | 16.6 | 27.4 |
| Male | Lakshadweep | 40.6 | 33.8 | 47.7 |
| Male | Madhya Pradesh | 9.2 | 1.6 | 39.1 |
| Male | Maharashtra | 20.0 | 17.3 | 22.9 |
| Male | Manipur | 24.8 | 19.4 | 31.0 |
| Male | Meghalaya | 30.9 | 25.6 | 36.7 |
| Male | Mizoram | 35.3 | 27.1 | 44.5 |
| Male | Nagaland | 37.1 | 29.9 | 44.8 |
| Male | Delhi | 28.3 | 23.4 | 33.8 |
| Male | Odisha | 35.2 | 29.8 | 41.0 |
| Male | Puducherry | 77.4 | 61.2 | 88.2 |
| Male | Punjab | 28.0 | 23.8 | 32.6 |
| Male | Rajasthan | 26.1 | 22.1 | 30.5 |
| Male | Sikkim | 41.8 | 35.6 | 48.3 |
| Male | Tamil Nadu | 61.3 | 55.8 | 66.5 |
| Male | Telangana | 43.3 | 35.5 | 51.4 |
| Male | Tripura | 33.4 | 24.1 | 44.1 |
| Male | Uttar Pradesh | 29.8 | 26.6 | 33.3 |
| Male | Uttarakhand | 23.7 | 18.1 | 30.5 |
| Male | West Bengal | 33.2 | 27.2 | 39.7 |

# Table K. State-level prevalence estimates for ‘treated’ by sex

| Sex | State | Estimate | Lower 95% CI | Upper 95% CI |
| --- | --- | --- | --- | --- |
| Female | Andaman and Nicobar Islands | 31.2 | 24.9 | 38.3 |
| Female | Andhra Pradesh | 18.3 | 16.1 | 20.8 |
| Female | Arunachal Pradesh | 13.0 | 11.5 | 14.5 |
| Female | Assam | 16.6 | 15.4 | 17.9 |
| Female | Bihar | 21.4 | 19.7 | 23.2 |
| Female | Chandigarh | 16.8 | 11.6 | 23.8 |
| Female | Chhattisgarh | 11.6 | 10.1 | 13.2 |
| Female | Dadra and Nagar Haveli | 22.8 | 15.1 | 32.9 |
| Female | Daman and Diu | 31.3 | 21.7 | 42.9 |
| Female | Goa | 20.7 | 16.9 | 25.1 |
| Female | Gujarat | 34.2 | 26.9 | 42.3 |
| Female | Haryana | 15.7 | 14.0 | 17.5 |
| Female | Himachal Pradesh | 18.3 | 15.9 | 20.9 |
| Female | Jammu and Kashmir | 18.8 | 16.6 | 21.2 |
| Female | Jharkhand | 29.2 | 27.1 | 31.3 |
| Female | Karnataka | 7.9 | 6.9 | 8.9 |
| Female | Kerala | 17.9 | 15.9 | 20.0 |
| Female | Lakshadweep | 26.8 | 24.1 | 29.6 |
| Female | Madhya Pradesh | 20.5 | 14.5 | 28.2 |
| Female | Maharashtra | 18.2 | 17.0 | 19.4 |
| Female | Manipur | 20.0 | 18.0 | 22.1 |
| Female | Meghalaya | 10.6 | 9.2 | 12.1 |
| Female | Mizoram | 29.4 | 25.5 | 33.5 |
| Female | Nagaland | 16.2 | 13.3 | 19.7 |
| Female | Delhi | 10.8 | 9.3 | 12.6 |
| Female | Odisha | 21.1 | 19.3 | 23.0 |
| Female | Puducherry | 12.8 | 8.1 | 19.6 |
| Female | Punjab | 18.1 | 16.6 | 19.7 |
| Female | Rajasthan | 16.0 | 14.8 | 17.4 |
| Female | Sikkim | 17.6 | 15.4 | 20.1 |
| Female | Tamil Nadu | 14.7 | 13.4 | 16.0 |
| Female | Telangana | 19.7 | 17.1 | 22.5 |
| Female | Tripura | 21.7 | 18.7 | 25.1 |
| Female | Uttar Pradesh | 12.8 | 12.0 | 13.7 |
| Female | Uttarakhand | 20.6 | 18.5 | 22.9 |
| Female | West Bengal | 19.4 | 17.5 | 21.6 |
| Male | Andaman and Nicobar Islands | 17.1 | 9.1 | 29.9 |
| Male | Andhra Pradesh | 10.6 | 7.3 | 15.2 |
| Male | Arunachal Pradesh | 8.1 | 5.7 | 11.4 |
| Male | Assam | 7.7 | 6.0 | 9.8 |
| Male | Bihar | 12.8 | 9.8 | 16.6 |
| Male | Chandigarh | 18.8 | 5.9 | 45.8 |
| Male | Chhattisgarh | 6.0 | 4.0 | 8.9 |
| Male | Dadra and Nagar Haveli | 14.6 | 4.6 | 37.9 |
| Male | Daman and Diu | 21.9 | 8.7 | 45.0 |
| Male | Goa | 12.4 | 4.0 | 32.3 |
| Male | Gujarat | 13.9 | 9.1 | 20.5 |
| Male | Haryana | 9.5 | 7.0 | 12.9 |
| Male | Himachal Pradesh | 10.8 | 8.3 | 14.1 |
| Male | Jammu and Kashmir | 7.0 | 4.9 | 10.0 |
| Male | Jharkhand | 12.8 | 10.4 | 15.7 |
| Male | Karnataka | 6.4 | 4.4 | 9.1 |
| Male | Kerala | 9.5 | 6.9 | 13.0 |
| Male | Lakshadweep | 17.5 | 12.8 | 23.5 |
| Male | Madhya Pradesh | 5.4 | 0.5 | 38.9 |
| Male | Maharashtra | 11.0 | 8.9 | 13.5 |
| Male | Manipur | 10.1 | 7.1 | 14.2 |
| Male | Meghalaya | 6.0 | 4.1 | 8.9 |
| Male | Mizoram | 18.5 | 12.3 | 26.7 |
| Male | Nagaland | 6.4 | 3.1 | 12.7 |
| Male | Delhi | 4.5 | 2.5 | 7.7 |
| Male | Odisha | 9.4 | 6.9 | 12.6 |
| Male | Puducherry | 9.6 | 4.2 | 20.6 |
| Male | Punjab | 7.3 | 5.3 | 9.9 |
| Male | Rajasthan | 7.2 | 5.5 | 9.3 |
| Male | Sikkim | 17.3 | 12.4 | 23.6 |
| Male | Tamil Nadu | 10.9 | 8.9 | 13.3 |
| Male | Telangana | 13.5 | 9.6 | 18.7 |
| Male | Tripura | 13.8 | 8.2 | 22.3 |
| Male | Uttar Pradesh | 6.1 | 4.8 | 7.6 |
| Male | Uttarakhand | 5.9 | 3.7 | 9.2 |
| Male | West Bengal | 9.6 | 6.5 | 14.1 |

# Table L. State-level prevalence estimates for ‘controlled’ by sex

| Sex | State | Estimate | Lower 95% CI | Upper 95% CI |
| --- | --- | --- | --- | --- |
| Female | Andaman and Nicobar Islands | 12.8 | 8.7 | 18.5 |
| Female | Andhra Pradesh | 11.3 | 9.5 | 13.4 |
| Female | Arunachal Pradesh | 6.0 | 5.1 | 7.2 |
| Female | Assam | 6.8 | 6.0 | 7.7 |
| Female | Bihar | 15.8 | 14.3 | 17.4 |
| Female | Chandigarh | 8.9 | 5.1 | 15.1 |
| Female | Chhattisgarh | 7.2 | 6.1 | 8.5 |
| Female | Dadra and Nagar Haveli | 16.0 | 9.7 | 25.1 |
| Female | Daman and Diu | 19.4 | 11.4 | 31.1 |
| Female | Goa | 13.6 | 10.5 | 17.4 |
| Female | Gujarat | 22.4 | 16.3 | 30.0 |
| Female | Haryana | 10.2 | 8.8 | 11.8 |
| Female | Himachal Pradesh | 13.2 | 11.1 | 15.7 |
| Female | Jammu and Kashmir | 10.1 | 8.6 | 11.9 |
| Female | Jharkhand | 18.3 | 16.7 | 20.1 |
| Female | Karnataka | 5.4 | 4.6 | 6.2 |
| Female | Kerala | 9.8 | 8.6 | 11.3 |
| Female | Lakshadweep | 18.7 | 16.3 | 21.4 |
| Female | Madhya Pradesh | 11.3 | 7.7 | 16.4 |
| Female | Maharashtra | 11.9 | 11.0 | 12.9 |
| Female | Manipur | 13.8 | 12.2 | 15.5 |
| Female | Meghalaya | 5.1 | 4.2 | 6.2 |
| Female | Mizoram | 20.8 | 17.4 | 24.8 |
| Female | Nagaland | 9.3 | 7.3 | 11.6 |
| Female | Delhi | 3.9 | 3.0 | 5.1 |
| Female | Odisha | 14.5 | 13.1 | 16.2 |
| Female | Puducherry | 7.6 | 4.8 | 11.9 |
| Female | Punjab | 8.0 | 7.0 | 9.1 |
| Female | Rajasthan | 10.6 | 9.6 | 11.8 |
| Female | Sikkim | 6.1 | 4.9 | 7.6 |
| Female | Tamil Nadu | 9.4 | 8.4 | 10.4 |
| Female | Telangana | 12.8 | 10.8 | 15.2 |
| Female | Tripura | 12.6 | 10.2 | 15.4 |
| Female | Uttar Pradesh | 8.8 | 8.2 | 9.6 |
| Female | Uttarakhand | 12.8 | 11.2 | 14.7 |
| Female | West Bengal | 8.6 | 7.4 | 10.0 |
| Male | Andaman and Nicobar Islands | 3.1 | 1.0 | 9.2 |
| Male | Andhra Pradesh | 4.9 | 2.8 | 8.3 |
| Male | Arunachal Pradesh | 3.5 | 2.1 | 5.8 |
| Male | Assam | 3.4 | 2.3 | 5.0 |
| Male | Bihar | 8.6 | 6.1 | 11.8 |
| Male | Chandigarh | 4.2 | 0.5 | 27.5 |
| Male | Chhattisgarh | 2.0 | 1.1 | 3.8 |
| Male | Dadra and Nagar Haveli | 3.6 | 0.4 | 26.3 |
| Male | Daman and Diu | 21.5 | 8.5 | 44.8 |
| Male | Goa | 10.2 | 2.7 | 31.6 |
| Male | Gujarat | 8.9 | 5.1 | 15.1 |
| Male | Haryana | 6.1 | 4.3 | 8.7 |
| Male | Himachal Pradesh | 6.2 | 4.3 | 8.9 |
| Male | Jammu and Kashmir | 3.4 | 2.2 | 5.4 |
| Male | Jharkhand | 7.1 | 5.3 | 9.4 |
| Male | Karnataka | 3.6 | 2.4 | 5.4 |
| Male | Kerala | 5.4 | 3.3 | 8.7 |
| Male | Lakshadweep | 12.9 | 8.8 | 18.5 |
| Male | Madhya Pradesh | 5.4 | 0.5 | 38.9 |
| Male | Maharashtra | 6.9 | 5.3 | 9.0 |
| Male | Manipur | 6.2 | 3.7 | 10.2 |
| Male | Meghalaya | 1.9 | 0.9 | 3.8 |
| Male | Mizoram | 14.7 | 8.9 | 23.2 |
| Male | Nagaland | 2.6 | 1.1 | 6.1 |
| Male | Delhi | 0.9 | 0.3 | 3.0 |
| Male | Odisha | 6.5 | 4.5 | 9.2 |
| Male | Puducherry | 2.8 | 0.7 | 10.3 |
| Male | Punjab | 2.1 | 1.3 | 3.4 |
| Male | Rajasthan | 3.7 | 2.5 | 5.4 |
| Male | Sikkim | 8.1 | 5.4 | 12.2 |
| Male | Tamil Nadu | 4.9 | 3.5 | 6.9 |
| Male | Telangana | 6.2 | 3.5 | 10.8 |
| Male | Tripura | 9.4 | 4.9 | 17.4 |
| Male | Uttar Pradesh | 4.1 | 3.2 | 5.4 |
| Male | Uttarakhand | 3.0 | 1.5 | 6.2 |
| Male | West Bengal | 2.0 | 0.8 | 4.6 |

# Table M. Percentage of total adult population in each state with unmet need for care^4,5^

|  | Unscreened | Unaware | Untreated | Uncontrolled |
| --- | --- | --- | --- | --- |
| Andaman and Nicobar Islands | 5.08 | 13.68 | 17.49 | 20.98 |
| Andhra Pradesh | 5.06 | 11.48 | 17.59 | 18.89 |
| Arunachal Pradesh | 7.57 | 16.05 | 22.10 | 23.49 |
| Assam | 6.43 | 16.48 | 21.63 | 23.35 |
| Bihar | 3.84 | 6.82 | 10.61 | 11.24 |
| Chandigarh | 1.44 | 9.26 | 16.96 | 19.45 |
| Chhattisgarh | 4.66 | 10.92 | 12.81 | 13.40 |
| Dadra and Nagar Haveli | 3.05 | 10.57 | 11.96 | 13.37 |
| Daman and Diu | 5.01 | 7.55 | 10.19 | 10.57 |
| Delhi | 1.74 | 5.25 | 11.35 | 12.03 |
| Goa | 1.98 | 11.06 | 12.72 | 13.71 |
| Gujarat | 5.25 | 11.65 | 13.96 | 14.61 |
| Haryana | 2.04 | 10.23 | 26.89 | 28.40 |
| Himachal Pradesh | 6.87 | 15.45 | 21.63 | 22.90 |
| Jammu and Kashmir | 3.76 | 10.38 | 16.86 | 18.53 |
| Jharkhand | 4.61 | 9.06 | 16.63 | 17.10 |
| Karnataka | 4.36 | 11.98 | 14.99 | 16.00 |
| Kerala | 1.43 | 7.00 | 10.24 | 11.03 |
| Lakshadweep | 2.53 | 11.09 | 13.29 | 14.12 |
| Madhya Pradesh | 5.04 | 9.14 | 11.13 | 11.80 |
| Maharashtra | 5.21 | 11.82 | 15.37 | 16.25 |
| Manipur | 2.58 | 13.44 | 21.06 | 22.15 |
| Meghalaya | 4.72 | 9.17 | 13.27 | 14.41 |
| Mizoram | 2.94 | 10.81 | 20.29 | 21.52 |
| Nagaland | 5.05 | 15.68 | 21.96 | 23.17 |
| Odisha | 6.07 | 9.54 | 15.32 | 16.22 |
| Puducherry | 3.25 | 7.79 | 35.58 | 38.01 |
| Punjab | 2.04 | 14.50 | 21.14 | 22.89 |
| Rajasthan | 4.21 | 9.19 | 12.99 | 13.63 |
| Sikkim | 4.38 | 15.76 | 25.31 | 28.42 |
| Tamil Nadu | 4.17 | 9.29 | 27.07 | 28.84 |
| Telangana | 4.61 | 10.55 | 19.73 | 21.40 |
| Tripura | 5.22 | 12.06 | 16.65 | 18.01 |
| Uttar Pradesh | 4.51 | 8.36 | 13.03 | 13.46 |
| Uttarakhand | 4.80 | 11.55 | 16.19 | 17.15 |
| West Bengal | 2.94 | 9.71 | 13.58 | 15.06 |

^4^ 95% confidence intervals for the estimates can be found in Table N - Q.

^5^ The total adult population was only those aged 15-49 years.

# Table N. Confidence intervals for Table M ‘Unscreened’ estimates

| State | Lower 95% CI | Upper 95% CI |
| --- | --- | --- |
| Andaman and Nicobar Islands | 3.49 | 7.12 |
| Andhra Pradesh | 4.28 | 5.93 |
| Arunachal Pradesh | 6.67 | 8.53 |
| Assam | 5.87 | 7.02 |
| Bihar | 3.47 | 4.23 |
| Chandigarh | 0.51 | 3.71 |
| Chhattisgarh | 4.18 | 5.16 |
| Dadra and Nagar Haveli | 1.51 | 5.49 |
| Daman and Diu | 2.95 | 7.49 |
| Delhi | 1.04 | 2.79 |
| Goa | 1.37 | 2.80 |
| Gujarat | 4.75 | 5.78 |
| Haryana | 1.64 | 2.55 |
| Himachal Pradesh | 5.98 | 7.84 |
| Jammu and Kashmir | 3.28 | 4.28 |
| Jharkhand | 4.14 | 5.11 |
| Karnataka | 3.83 | 4.93 |
| Kerala | 1.05 | 1.92 |
| Lakshadweep | 1.32 | 4.49 |
| Madhya Pradesh | 4.77 | 5.32 |
| Maharashtra | 4.52 | 5.96 |
| Manipur | 2.05 | 3.23 |
| Meghalaya | 4.04 | 5.47 |
| Mizoram | 1.90 | 4.42 |
| Nagaland | 4.33 | 5.85 |
| Odisha | 5.58 | 6.58 |
| Puducherry | 1.87 | 5.50 |
| Punjab | 1.62 | 2.56 |
| Rajasthan | 3.86 | 4.58 |
| Sikkim | 3.45 | 5.52 |
| Tamil Nadu | 3.66 | 4.73 |
| Telangana | 3.77 | 5.58 |
| Tripura | 4.07 | 6.57 |
| Uttar Pradesh | 4.26 | 4.76 |
| Uttarakhand | 4.15 | 5.50 |
| West Bengal | 2.50 | 3.43 |

# Table O. Confidence intervals for Table M ‘Unaware’ estimates

| State | **Lower 95% CI** | **Upper 95% CI** |
| --- | --- | --- |
| Andaman and Nicobar Islands | 11.30 | 15.85 |
| Andhra Pradesh | 10.36 | 12.58 |
| Arunachal Pradesh | 15.05 | 17.00 |
| Assam | 15.76 | 17.18 |
| Bihar | 6.36 | 7.27 |
| Chandigarh | 6.23 | 12.49 |
| Chhattisgarh | 10.53 | 11.27 |
| Dadra and Nagar Haveli | 8.48 | 12.12 |
| Daman and Diu | 5.11 | 9.78 |
| Delhi | 3.86 | 6.79 |
| Goa | 9.75 | 12.18 |
| Gujarat | 11.16 | 12.11 |
| Haryana | 8.93 | 11.62 |
| Himachal Pradesh | 14.56 | 16.31 |
| Jammu and Kashmir | 9.66 | 11.10 |
| Jharkhand | 8.37 | 9.76 |
| Karnataka | 11.30 | 12.62 |
| Kerala | 6.44 | 7.56 |
| Lakshadweep | 9.43 | 12.43 |
| Madhya Pradesh | 8.90 | 9.37 |
| Maharashtra | 11.08 | 12.51 |
| Manipur | 12.53 | 14.33 |
| Meghalaya | 8.32 | 10.02 |
| Mizoram | 9.68 | 11.96 |
| Nagaland | 14.91 | 16.41 |
| Odisha | 8.93 | 10.14 |
| Puducherry | 4.75 | 12.10 |
| Punjab | 13.67 | 15.31 |
| Rajasthan | 8.79 | 9.59 |
| Sikkim | 14.47 | 17.04 |
| Tamil Nadu | 8.28 | 10.38 |
| Telangana | 9.35 | 11.78 |
| Tripura | 10.76 | 13.28 |
| Uttar Pradesh | 8.06 | 8.65 |
| Uttarakhand | 10.75 | 12.32 |
| West Bengal | 9.13 | 10.27 |

# Table P. Confidence intervals for Table M ‘Untreated’ estimates

| State | **Lower 95% CI** | **Upper 95% CI** |
| --- | --- | --- |
| Andaman and Nicobar Islands | 15.75 | 18.88 |
| Andhra Pradesh | 17.05 | 18.06 |
| Arunachal Pradesh | 21.62 | 22.50 |
| Assam | 21.33 | 21.91 |
| Bihar | 10.35 | 10.84 |
| Chandigarh | 14.00 | 18.78 |
| Chhattisgarh | 12.58 | 13.01 |
| Dadra and Nagar Haveli | 9.90 | 13.20 |
| Daman and Diu | 7.86 | 11.74 |
| Delhi | 10.32 | 12.10 |
| Goa | 11.95 | 13.36 |
| Gujarat | 13.62 | 14.26 |
| Haryana | 26.16 | 27.54 |
| Himachal Pradesh | 21.10 | 22.07 |
| Jammu and Kashmir | 16.44 | 17.26 |
| Jharkhand | 16.37 | 16.84 |
| Karnataka | 14.64 | 15.30 |
| Kerala | 9.77 | 10.65 |
| Lakshadweep | 12.12 | 14.10 |
| Madhya Pradesh | 10.94 | 11.31 |
| Maharashtra | 14.95 | 15.75 |
| Manipur | 20.70 | 21.37 |
| Meghalaya | 12.50 | 13.96 |
| Mizoram | 19.68 | 20.79 |
| Nagaland | 21.54 | 22.30 |
| Odisha | 14.99 | 15.63 |
| Puducherry | 32.70 | 37.42 |
| Punjab | 20.73 | 21.52 |
| Rajasthan | 12.80 | 13.17 |
| Sikkim | 24.15 | 26.30 |
| Tamil Nadu | 26.60 | 27.50 |
| Telangana | 19.03 | 20.35 |
| Tripura | 15.74 | 17.41 |
| Uttar Pradesh | 12.91 | 13.14 |
| Uttarakhand | 15.79 | 16.55 |
| West Bengal | 13.21 | 13.91 |

# Table Q. Confidence intervals for Table M ‘Uncontrolled’ estimates

| State | **Lower 95% CI** | **Upper 95% CI** |
| --- | --- | --- |
| Andaman and Nicobar Islands | 20.20 | 21.50 |
| Andhra Pradesh | 18.49 | 19.21 |
| Arunachal Pradesh | 23.18 | 23.73 |
| Assam | 23.13 | 23.54 |
| Bihar | 11.02 | 11.44 |
| Chandigarh | 17.96 | 20.15 |
| Chhattisgarh | 13.26 | 13.51 |
| Dadra and Nagar Haveli | 12.07 | 13.98 |
| Daman and Diu | 8.09 | 12.07 |
| Delhi | 11.01 | 12.69 |
| Goa | 13.01 | 14.24 |
| Gujarat | 14.36 | 14.83 |
| Haryana | 27.79 | 28.92 |
| Himachal Pradesh | 22.57 | 23.16 |
| Jammu and Kashmir | 18.20 | 18.84 |
| Jharkhand | 16.93 | 17.23 |
| Karnataka | 15.66 | 16.27 |
| Kerala | 10.60 | 11.40 |
| Lakshadweep | 13.03 | 14.76 |
| Madhya Pradesh | 11.65 | 11.94 |
| Maharashtra | 15.87 | 16.57 |
| Manipur | 21.93 | 22.32 |
| Meghalaya | 13.66 | 15.05 |
| Mizoram | 21.09 | 21.84 |
| Nagaland | 22.94 | 23.33 |
| Odisha | 15.94 | 16.47 |
| Puducherry | 36.33 | 38.94 |
| Punjab | 22.68 | 23.07 |
| Rajasthan | 13.48 | 13.77 |
| Sikkim | 27.69 | 28.98 |
| Tamil Nadu | 28.47 | 29.16 |
| Telangana | 20.84 | 21.86 |
| Tripura | 17.20 | 18.62 |
| Uttar Pradesh | 13.36 | 13.55 |
| Uttarakhand | 16.83 | 17.41 |
| West Bengal | 14.87 | 15.22 |

# Table R. Absolute number of total adult population in each state with unmet need for care^6,7^

|  | **Unscreened** | **Unaware** | **Untreated** | **Uncontrolled** |
| --- | --- | --- | --- | --- |
| Andaman and Nicobar Islands | 12622 | 33970 | 43437 | 52100 |
| Andhra Pradesh | 1500917 | 3404656 | 5216250 | 5599727 |
| Arunachal Pradesh | 60172 | 127643 | 175729 | 186802 |
| Assam | 1157302 | 2967251 | 3894353 | 4203689 |
| Bihar | 1977518 | 3509437 | 5460062 | 5786839 |
| Chandigarh | 9855 | 63572 | 116413 | 133574 |
| Chhattisgarh | 677350 | 1587764 | 1863831 | 1949249 |
| Dadra and Nagar Haveli | 6550 | 22688 | 25683 | 28713 |
| Daman and Diu | 8593 | 12947 | 17469 | 18121 |
| Delhi | 183283 | 553519 | 1196921 | 1268683 |
| Goa | 17970 | 100577 | 115676 | 124676 |
| Gujarat | 1886226 | 4185145 | 5014935 | 5248026 |
| Haryana | 303424 | 1518431 | 3992435 | 4216503 |
| Himachal Pradesh | 282218 | 634840 | 888635 | 940909 |
| Jammu and Kashmir | 263783 | 728988 | 1184117 | 1301593 |
| Jharkhand | 813249 | 1598757 | 2932570 | 3015403 |
| Karnataka | 1615892 | 4443353 | 5559871 | 5932768 |
| Kerala | 280436 | 1372861 | 2006945 | 2162736 |
| Lakshadweep | 1005 | 4404 | 5278 | 5610 |
| Madhya Pradesh | 2039818 | 3697456 | 4504781 | 4775978 |
| Maharashtra | 3492566 | 7923077 | 10309721 | 10896960 |
| Manipur | 43805 | 228068 | 357351 | 375856 |
| Meghalaya | 74764 | 145340 | 210232 | 228338 |
| Mizoram | 18810 | 69297 | 130009 | 137876 |
| Nagaland | 58146 | 180561 | 252898 | 266892 |
| Odisha | 1475617 | 2319076 | 3724916 | 3943530 |
| Puducherry | 25301 | 60569 | 276787 | 295699 |
| Punjab | 342169 | 2434961 | 3551139 | 3844035 |
| Rajasthan | 1582239 | 3451790 | 4878301 | 5118082 |
| Sikkim | 16889 | 60699 | 97491 | 109453 |
| Tamil Nadu | 1853352 | 4132273 | 12037194 | 12820905 |
| Telangana | 976358 | 2235120 | 4180307 | 4533917 |
| Tripura | 117391 | 270989 | 374365 | 404736 |
| Uttar Pradesh | 4780649 | 8856115 | 13807095 | 14267516 |
| Uttarakhand | 274414 | 661085 | 926462 | 981294 |
| West Bengal | 1627522 | 5379391 | 7525438 | 8346724 |

^6^ 95% confidence intervals for the estimates can be found in Table S - V.

^7^ The total adult population was only those aged 15-49 years.

# Table S. Confidence intervals for Table R ‘Unscreened’ estimates

| State | **Lower 95% CI** | **Upper 95% CI** |
| --- | --- | --- |
| Andaman and Nicobar Islands | 8678 | 17684 |
| Andhra Pradesh | 1270364 | 1758020 |
| Arunachal Pradesh | 53034 | 67815 |
| Assam | 1057015 | 1263537 |
| Bihar | 1786626 | 2179570 |
| Chandigarh | 3526 | 25455 |
| Chhattisgarh | 607879 | 750597 |
| Dadra and Nagar Haveli | 3246 | 11786 |
| Daman and Diu | 5050 | 12839 |
| Delhi | 110214 | 294101 |
| Goa | 12428 | 25495 |
| Gujarat | 1704817 | 2076840 |
| Haryana | 242824 | 377850 |
| Himachal Pradesh | 245545 | 322021 |
| Jammu and Kashmir | 230589 | 300604 |
| Jharkhand | 730676 | 901685 |
| Karnataka | 1420426 | 1828385 |
| Kerala | 206372 | 376818 |
| Lakshadweep | 525 | 1785 |
| Madhya Pradesh | 1929955 | 2151898 |
| Maharashtra | 3028356 | 3996317 |
| Manipur | 34805 | 54772 |
| Meghalaya | 63939 | 86683 |
| Mizoram | 12173 | 28306 |
| Nagaland | 49868 | 67384 |
| Odisha | 1357318 | 1598857 |
| Puducherry | 14561 | 42821 |
| Punjab | 271288 | 429451 |
| Rajasthan | 1450374 | 1720984 |
| Sikkim | 13304 | 21246 |
| Tamil Nadu | 1628335 | 2104081 |
| Telangana | 799156 | 1181805 |
| Tripura | 91388 | 147783 |
| Uttar Pradesh | 4519678 | 5049615 |
| Uttarakhand | 237571 | 314793 |
| West Bengal | 1384421 | 1902389 |

# Table T. Confidence intervals for Table R ‘Unaware’ estimates

| State | **Lower 95% CI** | **Upper 95% CI** |
| --- | --- | --- |
| Andaman and Nicobar Islands | 28066 | 39350 |
| Andhra Pradesh | 3071410 | 3729254 |
| Arunachal Pradesh | 119684 | 135192 |
| Assam | 2836350 | 3092445 |
| Bihar | 3273095 | 3743640 |
| Chandigarh | 42740 | 85785 |
| Chhattisgarh | 1531763 | 1639200 |
| Dadra and Nagar Haveli | 18196 | 26025 |
| Daman and Diu | 8767 | 16764 |
| Delhi | 406837 | 716501 |
| Goa | 88683 | 110708 |
| Gujarat | 4009084 | 4348754 |
| Haryana | 1325685 | 1724666 |
| Himachal Pradesh | 598109 | 670037 |
| Jammu and Kashmir | 678387 | 779681 |
| Jharkhand | 1476059 | 1721207 |
| Karnataka | 4190678 | 4679041 |
| Kerala | 1263173 | 1481166 |
| Lakshadweep | 3748 | 4940 |
| Madhya Pradesh | 3600821 | 3790776 |
| Maharashtra | 7429016 | 8389978 |
| Manipur | 212525 | 243182 |
| Meghalaya | 131812 | 158759 |
| Mizoram | 62033 | 76641 |
| Nagaland | 171760 | 188974 |
| Odisha | 2171938 | 2465290 |
| Puducherry | 36950 | 94104 |
| Punjab | 2295234 | 2570577 |
| Rajasthan | 3299645 | 3599418 |
| Sikkim | 55743 | 65630 |
| Tamil Nadu | 3679587 | 4615215 |
| Telangana | 1980131 | 2495830 |
| Tripura | 241985 | 298582 |
| Uttar Pradesh | 8545990 | 9162177 |
| Uttarakhand | 615175 | 705026 |
| West Bengal | 5058501 | 5689839 |

# Table U. Confidence intervals for Table R ‘Untreated’ estimates

| State | **Lower 95% CI** | **Upper 95% CI** |
| --- | --- | --- |
| Andaman and Nicobar Islands | 39109 | 46893 |
| Andhra Pradesh | 5055041 | 5355759 |
| Arunachal Pradesh | 171943 | 178976 |
| Assam | 3839044 | 3945109 |
| Bihar | 5327806 | 5581197 |
| Chandigarh | 96098 | 128966 |
| Chhattisgarh | 1830109 | 1892553 |
| Dadra and Nagar Haveli | 21253 | 28338 |
| Daman and Diu | 13471 | 20134 |
| Delhi | 1089019 | 1276903 |
| Goa | 108625 | 121483 |
| Gujarat | 4892500 | 5120385 |
| Haryana | 3883950 | 4087803 |
| Himachal Pradesh | 867069 | 906990 |
| Jammu and Kashmir | 1154251 | 1211858 |
| Jharkhand | 2888149 | 2970108 |
| Karnataka | 5431308 | 5673707 |
| Kerala | 1915933 | 2088187 |
| Lakshadweep | 4815 | 5600 |
| Madhya Pradesh | 4427955 | 4575650 |
| Maharashtra | 10023497 | 10560933 |
| Manipur | 351174 | 362598 |
| Meghalaya | 198008 | 221152 |
| Mizoram | 126133 | 133228 |
| Nagaland | 248095 | 256847 |
| Odisha | 3644217 | 3798743 |
| Puducherry | 254357 | 291060 |
| Punjab | 3480773 | 3613536 |
| Rajasthan | 4805528 | 4944204 |
| Sikkim | 93022 | 101306 |
| Tamil Nadu | 11826414 | 12228272 |
| Telangana | 4030915 | 4310674 |
| Tripura | 353893 | 391410 |
| Uttar Pradesh | 13678953 | 13925983 |
| Uttarakhand | 903363 | 946647 |
| West Bengal | 7320022 | 7706982 |

# Table V. Confidence intervals for Table R ‘Uncontrolled’ estimates

| State | **Lower 95% CI** | **Upper 95% CI** |
| --- | --- | --- |
| Andaman and Nicobar Islands | 50169 | 53393 |
| Andhra Pradesh | 5483306 | 5695062 |
| Arunachal Pradesh | 184326 | 188770 |
| Assam | 4164871 | 4237013 |
| Bihar | 5671854 | 5889361 |
| Chandigarh | 123302 | 138381 |
| Chhattisgarh | 1929383 | 1965661 |
| Dadra and Nagar Haveli | 25922 | 30010 |
| Daman and Diu | 13873 | 20703 |
| Delhi | 1161616 | 1338602 |
| Goa | 118271 | 129476 |
| Gujarat | 5156408 | 5324557 |
| Haryana | 4124875 | 4293558 |
| Himachal Pradesh | 927486 | 951864 |
| Jammu and Kashmir | 1277812 | 1322883 |
| Jharkhand | 2986020 | 3039914 |
| Karnataka | 5808017 | 6033026 |
| Kerala | 2077130 | 2235418 |
| Lakshadweep | 5178 | 5862 |
| Madhya Pradesh | 4714745 | 4831017 |
| Maharashtra | 10641607 | 11109137 |
| Manipur | 372095 | 378806 |
| Meghalaya | 216391 | 238436 |
| Mizoram | 135169 | 139973 |
| Nagaland | 264257 | 268774 |
| Odisha | 3875738 | 4003914 |
| Puducherry | 282621 | 302958 |
| Punjab | 3809061 | 3873652 |
| Rajasthan | 5060393 | 5168497 |
| Sikkim | 106646 | 111612 |
| Tamil Nadu | 12656563 | 12962861 |
| Telangana | 4415598 | 4630398 |
| Tripura | 386543 | 418506 |
| Uttar Pradesh | 14162771 | 14363058 |
| Uttarakhand | 963151 | 996366 |
| West Bengal | 8238627 | 8435628 |

# Table W. Predictors of ‘screened’ and ‘aware’ among participants with hypertension^8^

Screened

|  | Rural | | | | Urban | | | |
| --- | --- | --- | --- | --- | --- | --- | --- | --- |
|  | Relative Risk | 95% CI (low) | 95% CI (high) | P Value | Relative Risk | 95% CI (low) | 95% CI (high) | P Value |
| Age group, y |  |  |  |  |  |  |  |  |
| 15-19 | 1(Reference) |  |  |  | 1(Reference) |  |  |  |
| 20-24 | 1.16 | 1.13 | 1.19 | <0.001 | 1.13 | 1.09 | 1.16 | <0.001 |
| 25-29 | 1.20 | 1.17 | 1.23 | <0.001 | 1.15 | 1.11 | 1.18 | <0.001 |
| 30-34 | 1.19 | 1.16 | 1.22 | <0.001 | 1.15 | 1.12 | 1.19 | <0.001 |
| 35-40 | 1.16 | 1.13 | 1.19 | <0.001 | 1.15 | 1.12 | 1.19 | <0.001 |
| 40-44 | 1.16 | 1.13 | 1.19 | <0.001 | 1.16 | 1.12 | 1.20 | <0.001 |
| 45-49 | 1.16 | 1.13 | 1.19 | <0.001 | 1.17 | 1.14 | 1.21 | <0.001 |
| Education |  |  |  |  |  |  |  |  |
| Primary school unfinished | 1(Reference) |  |  |  | 1(Reference) |  |  |  |
| Primary school finished | 1.05 | 1.03 | 1.06 | <0.001 | 1.03 | 1.01 | 1.04 | 0.001 |
| Secondary school unfinished | 1.07 | 1.06 | 1.08 | <0.001 | 1.04 | 1.03 | 1.05 | <0.001 |
| Secondary school or above | 1.10 | 1.08 | 1.11 | <0.001 | 1.07 | 1.06 | 1.08 | <0.001 |
| Household wealth quintile |  |  |  |  |  |  |  |  |
| Q1 (Poorest) | 1(Reference) |  |  |  | 1(Reference) |  |  |  |
| Q2 | 1.13 | 1.10 | 1.15 | <0.001 | 1.04 | 1.03 | 1.06 | <0.001 |
| Q3 | 1.18 | 1.16 | 1.20 | <0.001 | 1.05 | 1.04 | 1.07 | <0.001 |
| Q4 | 1.22 | 1.20 | 1.24 | <0.001 | 1.06 | 1.04 | 1.07 | <0.001 |
| Q5 (Richest) | 1.24 | 1.22 | 1.27 | <0.001 | 1.08 | 1.06 | 1.10 | <0.001 |
| BMI |  |  |  |  |  |  |  |  |
| <18.5 kg/m2 | 1.02 | 1.01 | 1.03 | 0.003 | 1.00 | 0.98 | 1.02 | 0.997 |
| 18.5-22.9 kg/m2 | 1(Reference) |  |  |  | 1(Reference) |  |  |  |
| 23.0-24.9 kg/m2 | 1.01 | 1.00 | 1.02 | 0.037 | 1.01 | 0.99 | 1.02 | 0.332 |
| 25.0-27.4 kg/m2 | 1.02 | 1.01 | 1.03 | 0.002 | 1.00 | 0.99 | 1.01 | 0.699 |
| 27.5-29.9 kg/m2 | 1.03 | 1.02 | 1.04 | <0.001 | 1.00 | 0.99 | 1.02 | 0.452 |
| 30.0 kg/m2 | 1.05 | 1.04 | 1.06 | <0.001 | 1.02 | 1.01 | 1.04 | <0.001 |
| Tobacco, smoked | 0.99 | 0.97 | 1.01 | 0.315 | 0.98 | 0.96 | 1.01 | 0.153 |
| Tobacco, smokeless | 0.98 | 0.97 | 0.99 | 0.007 | 0.98 | 0.96 | 0.99 | 0.002 |
| Currently married | 1.12 | 1.11 | 1.14 | <0.001 | 1.09 | 1.07 | 1.10 | <0.001 |
| Female | 1.24 | 1.22 | 1.26 | <0.001 | 1.18 | 1.16 | 1.20 | <0.001 |

Abbreviations: CI = Confidence Interval; BMI = Body Mass Index; Q = Quintile

^8^ These covariate-adjusted poisson regressions contained all predictor variables listed in the table (age group, wealth quintile, education, BMI, tobacco consumption, marital status, and sex) and a binary indicator for each district (district-level fixed effects) as explanatory variables. Sampling weights were not used. Standard errors were adjusted for clustering at the level of the primary sampling unit.

Aware

|  | Rural | | | | Urban | | | |
| --- | --- | --- | --- | --- | --- | --- | --- | --- |
|  | Relative Risk | 95% CI (low) | 95% CI (high) | P Value | Relative Risk | 95% CI (low) | 95% CI (high) | P Value |
| Age group, y |  |  |  |  |  |  |  |  |
| 15-19 | 1(Reference) |  |  |  | 1(Reference) |  |  |  |
| 20-24 | 1.06 | 1.03 | 1.10 | <0.001 | 1.04 | 0.99 | 1.09 | 0.095 |
| 25-29 | 1.04 | 1.00 | 1.08 | 0.039 | 0.98 | 0.93 | 1.02 | 0.353 |
| 30-34 | 0.98 | 0.94 | 1.01 | 0.213 | 0.94 | 0.89 | 0.98 | 0.006 |
| 35-40 | 0.92 | 0.89 | 0.95 | <0.001 | 0.89 | 0.85 | 0.93 | <0.001 |
| 40-44 | 0.91 | 0.88 | 0.95 | <0.001 | 0.91 | 0.87 | 0.95 | <0.001 |
| 45-49 | 0.94 | 0.90 | 0.97 | 0.001 | 0.97 | 0.92 | 1.02 | 0.183 |
| Education |  |  |  |  |  |  |  |  |
| Primary school unfinished | 1(Reference) |  |  |  | 1(Reference) |  |  |  |
| Primary school finished | 1.09 | 1.06 | 1.11 | <0.001 | 1.03 | 1.00 | 1.07 | 0.070 |
| Secondary school unfinished | 1.12 | 1.10 | 1.14 | <0.001 | 1.07 | 1.04 | 1.10 | <0.001 |
| Secondary school or above | 1.15 | 1.12 | 1.18 | <0.001 | 1.11 | 1.08 | 1.14 | <0.001 |
| Household wealth quintile |  |  |  |  |  |  |  |  |
| Q1 (Poorest) | 1(Reference) |  |  |  | 1(Reference) |  |  |  |
| Q2 | 1.19 | 1.15 | 1.23 | <0.001 | 1.04 | 1.01 | 1.07 | 0.015 |
| Q3 | 1.27 | 1.22 | 1.31 | <0.001 | 1.07 | 1.03 | 1.10 | <0.001 |
| Q4 | 1.33 | 1.28 | 1.38 | <0.001 | 1.07 | 1.04 | 1.11 | <0.001 |
| Q5 (Richest) | 1.35 | 1.30 | 1.40 | <0.001 | 1.11 | 1.07 | 1.15 | <0.001 |
| BMI |  |  |  |  |  |  |  |  |
| <18.5 kg/m2 | 1.08 | 1.06 | 1.10 | <0.001 | 1.07 | 1.04 | 1.11 | <0.001 |
| 18.5-22.9 kg/m2 | 1(Reference) |  |  |  | 1(Reference) |  |  |  |
| 23.0-24.9 kg/m2 | 0.94 | 0.92 | 0.96 | <0.001 | 0.95 | 0.92 | 0.97 | <0.001 |
| 25.0-27.4 kg/m2 | 0.93 | 0.91 | 0.95 | <0.001 | 0.94 | 0.92 | 0.97 | <0.001 |
| 27.5-29.9 kg/m2 | 0.95 | 0.93 | 0.98 | <0.001 | 0.97 | 0.95 | 1.00 | 0.063 |
| 30.0 kg/m2 | 1.01 | 0.98 | 1.03 | 0.605 | 1.03 | 1.00 | 1.05 | 0.060 |
| Tobacco, smoked | 1.04 | 1.00 | 1.08 | 0.046 | 1.03 | 0.97 | 1.08 | 0.330 |
| Tobacco, smokeless | 0.95 | 0.93 | 0.98 | 0.001 | 0.97 | 0.93 | 1.00 | 0.050 |
| Currently married | 1.15 | 1.13 | 1.18 | <0.001 | 1.14 | 1.11 | 1.17 | <0.001 |
| Female | 1.61 | 1.56 | 1.67 | <0.001 | 1.56 | 1.50 | 1.63 | <0.001 |

Abbreviations: CI = Confidence Interval; BMI = Body Mass Index; Q = Quintile

^8^ These covariate-adjusted poisson regressions contained all predictor variables listed in the table (age group, wealth quintile, education, BMI, tobacco consumption, marital status, and sex) and a binary indicator for each district (district-level fixed effects) as explanatory variables. Sampling weights were not used. Standard errors were adjusted for clustering at the level of the primary sampling unit.

# Table X. Predictors of reaching each cascade step using covariate-adjusted poisson regressions with an interaction term between marital status and sex^9^

Screened

|  | Rural |  |  |  | Urban |  |  |  |
| --- | --- | --- | --- | --- | --- | --- | --- | --- |
|  | Relative Risk | 95% CI (low) | 95% CI (high) | P Value | Relative Risk | 95% CI (low) | 95% CI (high) | P Value |
| Age group, y |  |  |  |  |  |  |  |  |
| 15-19 | 1(Reference) |  |  |  | 1(Reference) |  |  |  |
| 20-24 | 1.16 | 1.13 | 1.19 | <0.001 | 1.13 | 1.09 | 1.17 | <0.001 |
| 25-29 | 1.20 | 1.17 | 1.23 | <0.001 | 1.15 | 1.11 | 1.18 | <0.001 |
| 30-34 | 1.19 | 1.16 | 1.22 | <0.001 | 1.15 | 1.12 | 1.19 | <0.001 |
| 35-40 | 1.16 | 1.13 | 1.19 | <0.001 | 1.15 | 1.12 | 1.19 | <0.001 |
| 40-44 | 1.16 | 1.13 | 1.19 | <0.001 | 1.16 | 1.12 | 1.20 | <0.001 |
| 45-49 | 1.16 | 1.13 | 1.19 | <0.001 | 1.17 | 1.13 | 1.21 | <0.001 |
| Education |  |  |  |  |  |  |  |  |
| Primary school unfinished | 1(Reference) |  |  |  | 1(Reference) |  |  |  |
| Primary school finished | 1.05 | 1.03 | 1.06 | <0.001 | 1.03 | 1.01 | 1.04 | 0.001 |
| Secondary school unfinished | 1.07 | 1.06 | 1.08 | <0.001 | 1.04 | 1.03 | 1.05 | <0.001 |
| Secondary school or above | 1.10 | 1.08 | 1.11 | <0.001 | 1.07 | 1.06 | 1.08 | <0.001 |
| Household wealth quintile |  |  |  |  |  |  |  |  |
| Q1 (Poorest) | 1(Reference) |  |  |  | 1(Reference) |  |  |  |
| Q2 | 1.13 | 1.10 | 1.15 | <0.001 | 1.04 | 1.03 | 1.06 | <0.001 |
| Q3 | 1.18 | 1.16 | 1.20 | <0.001 | 1.05 | 1.04 | 1.07 | <0.001 |
| Q4 | 1.22 | 1.20 | 1.24 | <0.001 | 1.06 | 1.04 | 1.08 | <0.001 |
| Q5 (Richest) | 1.24 | 1.22 | 1.27 | <0.001 | 1.08 | 1.06 | 1.10 | <0.001 |
| BMI |  |  |  |  |  |  |  |  |
| <18.5 kg/m2 | 1.02 | 1.01 | 1.03 | 0.004 | 1.00 | 0.98 | 1.02 | 0.961 |
| 18.5-22.9 kg/m2 | 1(Reference) |  |  |  | 1(Reference) |  |  |  |
| 23.0-24.9 kg/m2 | 1.01 | 1.00 | 1.02 | 0.036 | 1.01 | 0.99 | 1.02 | 0.321 |
| 25.0-27.4 kg/m2 | 1.02 | 1.01 | 1.03 | 0.001 | 1.00 | 0.99 | 1.01 | 0.674 |
| 27.5-29.9 kg/m2 | 1.03 | 1.02 | 1.04 | <0.001 | 1.00 | 0.99 | 1.02 | 0.426 |
| 30.0 kg/m2 | 1.05 | 1.04 | 1.06 | <0.001 | 1.03 | 1.01 | 1.04 | <0.001 |
| Tobacco, smoked | 0.99 | 0.97 | 1.01 | 0.268 | 0.98 | 0.96 | 1.00 | 0.127 |
| Tobacco, smokeless | 0.98 | 0.97 | 0.99 | 0.005 | 0.98 | 0.96 | 0.99 | 0.001 |
| Currently married | 1.16 | 1.12 | 1.20 | <0.001 | 1.13 | 1.09 | 1.18 | <0.001 |
| Female | 1.28 | 1.23 | 1.32 | <0.001 | 1.23 | 1.18 | 1.27 | <0.001 |
| Interaction Currently married-Female | 0.96 | 0.93 | 1.00 | 0.055 | 0.95 | 0.92 | 0.99 | 0.019 |

Abbreviations: CI = Confidence Interval; BMI = Body Mass Index; Q = Quintile

^9^ These covariate-adjusted poisson regressions contained all predictor variables listed in the table (age group, wealth quintile, education, BMI, tobacco consumption, marital status, and sex), a binary indicator for each district (district-level fixed effects), and an interaction term between currently married and sex as explanatory variables. Sampling weights were not used. Standard errors were adjusted for clustering at the level of the primary sampling unit.

Aware

|  | Rural |  |  |  | Urban |  |  |  |
| --- | --- | --- | --- | --- | --- | --- | --- | --- |
|  | Relative Risk | 95% CI (low) | 95% CI (high) | P Value | Relative Risk | 95% CI (low) | 95% CI (high) | P Value |
| Age group, y |  |  |  |  |  |  |  |  |
| 15-19 | 1(Reference) |  |  |  | 1(Reference) |  |  |  |
| 20-24 | 1.06 | 1.03 | 1.10 | <0.001 | 1.04 | 0.99 | 1.09 | 0.089 |
| 25-29 | 1.04 | 1.00 | 1.08 | 0.039 | 0.98 | 0.93 | 1.03 | 0.366 |
| 30-34 | 0.98 | 0.94 | 1.01 | 0.213 | 0.94 | 0.89 | 0.98 | 0.006 |
| 35-40 | 0.92 | 0.89 | 0.95 | <0.001 | 0.89 | 0.85 | 0.93 | <0.001 |
| 40-44 | 0.91 | 0.88 | 0.95 | <0.001 | 0.91 | 0.86 | 0.95 | <0.001 |
| 45-49 | 0.94 | 0.90 | 0.97 | <0.001 | 0.97 | 0.92 | 1.01 | 0.170 |
| Education |  |  |  |  |  |  |  |  |
| Primary school unfinished | 1(Reference) |  |  |  | 1(Reference) |  |  |  |
| Primary school finished | 1.09 | 1.06 | 1.11 | <0.001 | 1.03 | 1.00 | 1.07 | 0.072 |
| Secondary school unfinished | 1.12 | 1.10 | 1.14 | <0.001 | 1.07 | 1.04 | 1.09 | <0.001 |
| Secondary school or above | 1.15 | 1.12 | 1.18 | <0.001 | 1.11 | 1.08 | 1.14 | <0.001 |
| Household wealth quintile |  |  |  |  |  |  |  |  |
| Q1 (Poorest) | 1(Reference) |  |  |  | 1(Reference) |  |  |  |
| Q2 | 1.19 | 1.15 | 1.23 | <0.001 | 1.04 | 1.01 | 1.07 | 0.015 |
| Q3 | 1.27 | 1.22 | 1.31 | <0.001 | 1.07 | 1.03 | 1.10 | <0.001 |
| Q4 | 1.33 | 1.28 | 1.38 | <0.001 | 1.07 | 1.04 | 1.11 | <0.001 |
| Q5 (Richest) | 1.35 | 1.30 | 1.40 | <0.001 | 1.11 | 1.07 | 1.15 | <0.001 |
| BMI |  |  |  |  |  |  |  |  |
| <18.5 kg/m2 | 1.08 | 1.06 | 1.10 | <0.001 | 1.07 | 1.04 | 1.11 | <0.001 |
| 18.5-22.9 kg/m2 | 1(Reference) |  |  |  | 1(Reference) |  |  |  |
| 23.0-24.9 kg/m2 | 0.94 | 0.92 | 0.96 | <0.001 | 0.95 | 0.92 | 0.97 | <0.001 |
| 25.0-27.4 kg/m2 | 0.93 | 0.91 | 0.95 | <0.001 | 0.94 | 0.92 | 0.97 | <0.001 |
| 27.5-29.9 kg/m2 | 0.95 | 0.93 | 0.98 | <0.001 | 0.97 | 0.95 | 1.00 | 0.065 |
| 30.0 kg/m2 | 1.01 | 0.98 | 1.03 | 0.605 | 1.03 | 1.00 | 1.06 | 0.056 |
| Tobacco, smoked | 1.04 | 1.00 | 1.08 | 0.046 | 1.02 | 0.97 | 1.08 | 0.356 |
| Tobacco, smokeless | 0.95 | 0.93 | 0.98 | 0.001 | 0.97 | 0.93 | 1.00 | 0.047 |
| Currently married | 1.15 | 1.08 | 1.22 | <0.001 | 1.18 | 1.09 | 1.27 | <0.001 |
| Female | 1.61 | 1.51 | 1.72 | <0.001 | 1.61 | 1.49 | 1.73 | <0.001 |
| Interaction Currently married-Female | 1.00 | 0.94 | 1.07 | 0.986 | 0.96 | 0.89 | 1.04 | 0.320 |

Abbreviations: CI = Confidence Interval; BMI = Body Mass Index; Q = Quintile

^9^ These covariate-adjusted poisson regressions contained all predictor variables listed in the table (age group, wealth quintile, education, BMI, tobacco consumption, marital status, and sex), a binary indicator for each district (district-level fixed effects), and an interaction term between currently married and sex as explanatory variables. Sampling weights were not used. Standard errors were adjusted for clustering at the level of the primary sampling unit.

Treated

|  | Rural |  |  |  | Urban |  |  |  |
| --- | --- | --- | --- | --- | --- | --- | --- | --- |
|  | Relative Risk | 95% CI (low) | 95% CI (high) | P Value | Relative Risk | 95% CI (low) | 95% CI (high) | P Value |
| Age group, y |  |  |  |  |  |  |  |  |
| 15-19 | 1(Reference) |  |  |  | 1(Reference) |  |  |  |
| 20-24 | 0.90 | 0.81 | 0.99 | 0.035 | 0.98 | 0.82 | 1.16 | 0.790 |
| 25-29 | 0.94 | 0.85 | 1.04 | 0.199 | 0.88 | 0.74 | 1.04 | 0.129 |
| 30-34 | 0.96 | 0.87 | 1.07 | 0.472 | 1.00 | 0.85 | 1.18 | 0.999 |
| 35-40 | 1.06 | 0.96 | 1.17 | 0.273 | 1.15 | 0.98 | 1.35 | 0.081 |
| 40-44 | 1.19 | 1.08 | 1.31 | <0.001 | 1.52 | 1.30 | 1.78 | <0.001 |
| 45-49 | 1.38 | 1.25 | 1.51 | <0.001 | 1.92 | 1.64 | 2.25 | <0.001 |
| Education |  |  |  |  |  |  |  |  |
| Primary school unfinished | 1(Reference) |  |  |  | 1(Reference) |  |  |  |
| Primary school finished | 1.01 | 0.95 | 1.08 | 0.664 | 1.05 | 0.96 | 1.15 | 0.252 |
| Secondary school unfinished | 1.03 | 0.99 | 1.08 | 0.165 | 0.98 | 0.92 | 1.04 | 0.476 |
| Secondary school or above | 1.00 | 0.94 | 1.06 | 0.948 | 0.96 | 0.90 | 1.03 | 0.266 |
| Household wealth quintile |  |  |  |  |  |  |  |  |
| Q1 (Poorest) | 1(Reference) |  |  |  | 1(Reference) |  |  |  |
| Q2 | 1.26 | 1.17 | 1.36 | <0.001 | 1.11 | 1.03 | 1.20 | 0.008 |
| Q3 | 1.34 | 1.24 | 1.44 | <0.001 | 1.17 | 1.08 | 1.27 | <0.001 |
| Q4 | 1.44 | 1.34 | 1.55 | <0.001 | 1.27 | 1.17 | 1.38 | <0.001 |
| Q5 (Richest) | 1.54 | 1.42 | 1.66 | <0.001 | 1.34 | 1.22 | 1.47 | <0.001 |
| BMI |  |  |  |  |  |  |  |  |
| <18.5 kg/m2 | 1.10 | 1.04 | 1.16 | <0.001 | 1.09 | 0.97 | 1.21 | 0.135 |
| 18.5-22.9 kg/m2 | 1(Reference) |  |  |  | 1(Reference) |  |  |  |
| 23.0-24.9 kg/m2 | 1.02 | 0.97 | 1.07 | 0.446 | 1.01 | 0.94 | 1.09 | 0.764 |
| 25.0-27.4 kg/m2 | 1.07 | 1.01 | 1.12 | 0.011 | 1.11 | 1.04 | 1.19 | 0.002 |
| 27.5-29.9 kg/m2 | 1.17 | 1.10 | 1.24 | <0.001 | 1.28 | 1.19 | 1.37 | <0.001 |
| 30.0 kg/m2 | 1.46 | 1.38 | 1.54 | <0.001 | 1.61 | 1.51 | 1.72 | <0.001 |
| Tobacco, smoked | 0.93 | 0.86 | 1.01 | 0.097 | 1.02 | 0.91 | 1.14 | 0.775 |
| Tobacco, smokeless | 0.98 | 0.92 | 1.04 | 0.443 | 0.99 | 0.91 | 1.06 | 0.728 |
| Currently married | 1.19 | 1.01 | 1.39 | 0.035 | 1.27 | 1.04 | 1.55 | 0.021 |
| Female | 1.89 | 1.62 | 2.21 | <0.001 | 1.89 | 1.55 | 2.29 | <0.001 |
| Interaction Currently married-Female | 0.93 | 0.79 | 1.09 | 0.370 | 0.85 | 0.69 | 1.04 | 0.117 |

Abbreviations: CI = Confidence Interval; BMI = Body Mass Index; Q = Quintile

^9^ These covariate-adjusted poisson regressions contained all predictor variables listed in the table (age group, wealth quintile, education, BMI, tobacco consumption, marital status, and sex), a binary indicator for each district (district-level fixed effects), and an interaction term between currently married and sex as explanatory variables. Sampling weights were not used. Standard errors were adjusted for clustering at the level of the primary sampling unit.

Controlled

|  | Rural |  |  |  | Urban |  |  |  |
| --- | --- | --- | --- | --- | --- | --- | --- | --- |
|  | Relative Risk | 95% CI (low) | 95% CI (high) | P Value | Relative Risk | 95% CI (low) | 95% CI (high) | P Value |
| Age group, y |  |  |  |  |  |  |  |  |
| 15-19 | 1(Reference) |  |  |  | 1(Reference) |  |  |  |
| 20-24 | 0.88 | 0.79 | 0.97 | 0.015 | 0.98 | 0.81 | 1.18 | 0.813 |
| 25-29 | 0.89 | 0.80 | 0.99 | 0.033 | 0.82 | 0.67 | 0.99 | 0.035 |
| 30-34 | 0.84 | 0.75 | 0.94 | 0.002 | 0.81 | 0.67 | 0.98 | 0.027 |
| 35-40 | 0.78 | 0.70 | 0.88 | <0.001 | 0.77 | 0.64 | 0.94 | 0.008 |
| 40-44 | 0.77 | 0.69 | 0.86 | <0.001 | 0.94 | 0.78 | 1.13 | 0.504 |
| 45-49 | 0.79 | 0.71 | 0.89 | <0.001 | 1.14 | 0.95 | 1.37 | 0.148 |
| Education |  |  |  |  |  |  |  |  |
| Primary school unfinished | 1(Reference) |  |  |  | 1(Reference) |  |  |  |
| Primary school finished | 1.02 | 0.93 | 1.11 | 0.713 | 1.07 | 0.95 | 1.21 | 0.268 |
| Secondary school unfinished | 1.06 | 1.00 | 1.12 | 0.058 | 0.98 | 0.90 | 1.07 | 0.632 |
| Secondary school or above | 1.06 | 0.98 | 1.15 | 0.148 | 0.97 | 0.88 | 1.07 | 0.569 |
| Household wealth quintile |  |  |  |  |  |  |  |  |
| Q1 (Poorest) | 1(Reference) |  |  |  | 1(Reference) |  |  |  |
| Q2 | 1.21 | 1.11 | 1.32 | <0.001 | 1.15 | 1.03 | 1.28 | 0.011 |
| Q3 | 1.30 | 1.18 | 1.42 | <0.001 | 1.15 | 1.03 | 1.29 | 0.012 |
| Q4 | 1.38 | 1.25 | 1.51 | <0.001 | 1.37 | 1.23 | 1.54 | <0.001 |
| Q5 (Richest) | 1.44 | 1.30 | 1.59 | <0.001 | 1.41 | 1.24 | 1.60 | <0.001 |
| BMI |  |  |  |  |  |  |  |  |
| <18.5 kg/m2 | 1.19 | 1.12 | 1.26 | <0.001 | 1.15 | 1.01 | 1.30 | 0.031 |
| 18.5-22.9 kg/m2 | 1(Reference) |  |  |  | 1(Reference) |  |  |  |
| 23.0-24.9 kg/m2 | 0.86 | 0.81 | 0.92 | <0.001 | 0.91 | 0.83 | 1.00 | 0.049 |
| 25.0-27.4 kg/m2 | 0.82 | 0.77 | 0.88 | <0.001 | 0.90 | 0.83 | 0.99 | 0.030 |
| 27.5-29.9 kg/m2 | 0.86 | 0.79 | 0.94 | 0.001 | 0.98 | 0.89 | 1.08 | 0.661 |
| 30.0 kg/m2 | 1.04 | 0.95 | 1.13 | 0.410 | 1.23 | 1.13 | 1.35 | <0.001 |
| Tobacco, smoked | 1.01 | 0.91 | 1.13 | 0.842 | 0.97 | 0.82 | 1.15 | 0.755 |
| Tobacco, smokeless | 0.93 | 0.86 | 1.01 | 0.074 | 1.01 | 0.90 | 1.13 | 0.923 |
| Currently married | 1.04 | 0.86 | 1.26 | 0.697 | 1.20 | 0.93 | 1.55 | 0.159 |
| Female | 1.89 | 1.58 | 2.26 | <0.001 | 2.05 | 1.63 | 2.59 | <0.001 |
| Interaction Currently married-Female | 1.09 | 0.89 | 1.32 | 0.414 | 0.93 | 0.72 | 1.22 | 0.615 |

Abbreviations: CI = Confidence Interval; BMI = Body Mass Index; Q = Quintile

^9^ These covariate-adjusted poisson regressions contained all predictor variables listed in the table (age group, wealth quintile, education, BMI, tobacco consumption, marital status, and sex), a binary indicator for each district (district-level fixed effects), and an interaction term between currently married and sex as explanatory variables. Sampling weights were not used. Standard errors were adjusted for clustering at the level of the primary sampling unit.

# Table Y. Predictors of each cascade step using covariate-adjusted poisson regressions with sampling weights^10^

Screened

|  | Rural | | | | Urban | | | |
| --- | --- | --- | --- | --- | --- | --- | --- | --- |
|  | Relative Risk | 95% CI (low) | 95% CI (high) | P Value | Relative Risk | 95% CI (low) | 95% CI (high) | P Value |
| Age group, y |  |  |  |  |  |  |  |  |
| 15-19 | 1(Reference) |  |  |  | 1(Reference) |  |  |  |
| 20-24 | 1.10 | 1.06 | 1.14 | <0.001 | 1.16 | 1.10 | 1.21 | <0.001 |
| 25-29 | 1.16 | 1.12 | 1.21 | <0.001 | 1.17 | 1.12 | 1.23 | <0.001 |
| 30-34 | 1.15 | 1.10 | 1.19 | <0.001 | 1.14 | 1.08 | 1.19 | <0.001 |
| 35-40 | 1.13 | 1.09 | 1.18 | <0.001 | 1.18 | 1.13 | 1.24 | <0.001 |
| 40-44 | 1.15 | 1.11 | 1.20 | <0.001 | 1.22 | 1.16 | 1.28 | <0.001 |
| 45-49 | 1.17 | 1.13 | 1.22 | <0.001 | 1.23 | 1.17 | 1.29 | <0.001 |
| Education |  |  |  |  |  |  |  |  |
| Primary school unfinished | 1(Reference) |  |  |  | 1(Reference) |  |  |  |
| Primary school finished | 1.04 | 1.01 | 1.06 | 0.009 | 1.01 | 0.98 | 1.05 | 0.453 |
| Secondary school unfinished | 1.08 | 1.06 | 1.10 | <0.001 | 1.04 | 1.02 | 1.07 | <0.001 |
| Secondary school or above | 1.12 | 1.09 | 1.14 | <0.001 | 1.10 | 1.08 | 1.13 | <0.001 |
| Household wealth quintile |  |  |  |  |  |  |  |  |
| Q1 (Poorest) | 1(Reference) |  |  |  | 1(Reference) |  |  |  |
| Q2 | 1.14 | 1.11 | 1.17 | <0.001 | 1.05 | 1.02 | 1.07 | 0.001 |
| Q3 | 1.19 | 1.16 | 1.22 | <0.001 | 1.02 | 0.99 | 1.05 | 0.121 |
| Q4 | 1.22 | 1.19 | 1.26 | <0.001 | 1.07 | 1.04 | 1.09 | <0.001 |
| Q5 (Richest) | 1.25 | 1.22 | 1.29 | <0.001 | 1.11 | 1.08 | 1.15 | <0.001 |
| BMI |  |  |  |  |  |  |  |  |
| <18.5 kg/m2 | 1.00 | 0.98 | 1.02 | 0.748 | 1.02 | 0.99 | 1.06 | 0.207 |
| 18.5-22.9 kg/m2 | 1(Reference) |  |  |  | 1(Reference) |  |  |  |
| 23.0-24.9 kg/m2 | 1.01 | 0.99 | 1.03 | 0.439 | 1.03 | 1.01 | 1.06 | 0.003 |
| 25.0-27.4 kg/m2 | 1.02 | 1.00 | 1.04 | 0.073 | 0.99 | 0.97 | 1.01 | 0.492 |
| 27.5-29.9 kg/m2 | 1.01 | 0.98 | 1.03 | 0.543 | 1.03 | 1.00 | 1.05 | 0.028 |
| 30.0 kg/m2 | 1.03 | 1.00 | 1.06 | 0.025 | 1.08 | 1.05 | 1.10 | <0.001 |
| Tobacco, smoked | 0.98 | 0.96 | 1.00 | 0.051 | 1.01 | 0.98 | 1.03 | 0.563 |
| Tobacco, smokeless | 0.99 | 0.97 | 1.01 | 0.280 | 0.92 | 0.90 | 0.94 | <0.001 |
| Currently married | 1.09 | 1.07 | 1.12 | <0.001 | 1.10 | 1.07 | 1.12 | <0.001 |
| Female | 1.26 | 1.24 | 1.28 | <0.001 | 1.18 | 1.16 | 1.20 | <0.001 |

Abbreviations: CI = Confidence Interval; BMI = Body Mass Index; Q = Quintile

^10^ These covariate-adjusted poisson regressions contained all predictor variables listed in the table (age group, wealth quintile, education, BMI, tobacco consumption, marital status, and sex) and a binary indicator for each district (district-level fixed effects) as explanatory variables. Sampling weights to adjust for the complex survey design and for the differing number of women and men were used. Standard errors were adjusted for clustering at the level of the primary sampling unit.

Aware

|  | Rural | | | | Urban | | | |
| --- | --- | --- | --- | --- | --- | --- | --- | --- |
|  | Relative Risk | 95% CI (low) | 95% CI (high) | P Value | Relative Risk | 95% CI (low) | 95% CI (high) | P Value |
| Age group, y |  |  |  |  |  |  |  |  |
| 15-19 | 1(Reference) |  |  |  | 1(Reference) |  |  |  |
| 20-24 | 0.98 | 0.94 | 1.03 | 0.491 | 1.02 | 0.97 | 1.08 | 0.449 |
| 25-29 | 0.96 | 0.92 | 1.00 | 0.074 | 0.92 | 0.87 | 0.97 | 0.003 |
| 30-34 | 0.90 | 0.86 | 0.95 | <0.001 | 0.89 | 0.84 | 0.94 | <0.001 |
| 35-40 | 0.85 | 0.81 | 0.89 | <0.001 | 0.89 | 0.84 | 0.94 | <0.001 |
| 40-44 | 0.88 | 0.84 | 0.93 | <0.001 | 0.92 | 0.87 | 0.98 | 0.005 |
| 45-49 | 0.92 | 0.88 | 0.96 | <0.001 | 0.99 | 0.93 | 1.04 | 0.646 |
| Education |  |  |  |  |  |  |  |  |
| Primary school unfinished | 1(Reference) |  |  |  | 1(Reference) |  |  |  |
| Primary school finished | 1.06 | 1.02 | 1.10 | 0.001 | 0.99 | 0.94 | 1.03 | 0.568 |
| Secondary school unfinished | 1.13 | 1.10 | 1.15 | <0.001 | 1.09 | 1.05 | 1.12 | <0.001 |
| Secondary school or above | 1.14 | 1.10 | 1.17 | <0.001 | 1.14 | 1.10 | 1.18 | <0.001 |
| Household wealth quintile |  |  |  |  |  |  |  |  |
| Q1 (Poorest) | 1(Reference) |  |  |  | 1(Reference) |  |  |  |
| Q2 | 1.20 | 1.16 | 1.24 | <0.001 | 1.03 | 1.00 | 1.07 | 0.053 |
| Q3 | 1.28 | 1.23 | 1.32 | <0.001 | 1.04 | 1.01 | 1.08 | 0.016 |
| Q4 | 1.30 | 1.25 | 1.35 | <0.001 | 1.08 | 1.04 | 1.12 | <0.001 |
| Q5 (Richest) | 1.33 | 1.28 | 1.38 | <0.001 | 1.13 | 1.09 | 1.17 | <0.001 |
| BMI |  |  |  |  |  |  |  |  |
| <18.5 kg/m2 | 1.09 | 1.06 | 1.12 | <0.001 | 1.09 | 1.05 | 1.14 | <0.001 |
| 18.5-22.9 kg/m2 | 1(Reference) |  |  |  | 1(Reference) |  |  |  |
| 23.0-24.9 kg/m2 | 0.91 | 0.89 | 0.93 | <0.001 | 0.93 | 0.90 | 0.95 | <0.001 |
| 25.0-27.4 kg/m2 | 0.92 | 0.89 | 0.94 | <0.001 | 0.92 | 0.90 | 0.95 | <0.001 |
| 27.5-29.9 kg/m2 | 0.89 | 0.86 | 0.92 | <0.001 | 1.05 | 1.02 | 1.08 | 0.003 |
| 30.0 kg/m2 | 0.95 | 0.92 | 0.99 | 0.010 | 1.04 | 1.01 | 1.07 | 0.014 |
| Tobacco, smoked | 1.06 | 1.03 | 1.09 | <0.001 | 1.10 | 1.06 | 1.13 | <0.001 |
| Tobacco, smokeless | 0.94 | 0.92 | 0.97 | <0.001 | 0.81 | 0.78 | 0.84 | <0.001 |
| Currently married | 1.11 | 1.08 | 1.13 | <0.001 | 1.12 | 1.08 | 1.15 | <0.001 |
| Female | 1.58 | 1.55 | 1.61 | <0.001 | 1.51 | 1.48 | 1.55 | <0.001 |

Abbreviations: CI = Confidence Interval; BMI = Body Mass Index; Q = Quintile

^10^ These covariate-adjusted poisson regressions contained all predictor variables listed in the table (age group, wealth quintile, education, BMI, tobacco consumption, marital status, and sex) and a binary indicator for each district (district-level fixed effects) as explanatory variables. Sampling weights to adjust for the complex survey design and for the differing number of women and men were used. Standard errors were adjusted for clustering at the level of the primary sampling unit.

Treated

|  | Rural | | | | Urban | | | |
| --- | --- | --- | --- | --- | --- | --- | --- | --- |
|  | Relative Risk | 95% CI (low) | 95% CI (high) | P Value | Relative Risk | 95% CI (low) | 95% CI (high) | P Value |
| Age group, y |  |  |  |  |  |  |  |  |
| 15-19 | 1(Reference) |  |  |  | 1(Reference) |  |  |  |
| 20-24 | 0.67 | 0.61 | 0.73 | <0.001 | 1.13 | 0.98 | 1.30 | 0.087 |
| 25-29 | 0.73 | 0.67 | 0.80 | <0.001 | 0.85 | 0.74 | 0.97 | 0.021 |
| 30-34 | 0.77 | 0.70 | 0.84 | <0.001 | 0.98 | 0.86 | 1.13 | 0.802 |
| 35-40 | 0.79 | 0.73 | 0.87 | <0.001 | 1.17 | 1.02 | 1.34 | 0.026 |
| 40-44 | 1.03 | 0.94 | 1.12 | 0.518 | 1.77 | 1.55 | 2.02 | <0.001 |
| 45-49 | 1.19 | 1.09 | 1.29 | <0.001 | 2.16 | 1.89 | 2.46 | <0.001 |
| Education |  |  |  |  |  |  |  |  |
| Primary school unfinished | 1(Reference) |  |  |  | 1(Reference) |  |  |  |
| Primary school finished | 1.03 | 0.97 | 1.10 | 0.330 | 0.97 | 0.89 | 1.05 | 0.395 |
| Secondary school unfinished | 1.03 | 0.99 | 1.07 | 0.215 | 1.02 | 0.97 | 1.07 | 0.461 |
| Secondary school or above | 1.10 | 1.04 | 1.16 | 0.001 | 1.06 | 1.00 | 1.12 | 0.071 |
| Household wealth quintile |  |  |  |  |  |  |  |  |
| Q1 (Poorest) | 1(Reference) |  |  |  | 1(Reference) |  |  |  |
| Q2 | 1.11 | 1.04 | 1.18 | 0.002 | 1.00 | 0.94 | 1.06 | 0.954 |
| Q3 | 1.25 | 1.17 | 1.33 | <0.001 | 1.17 | 1.10 | 1.25 | <0.001 |
| Q4 | 1.28 | 1.19 | 1.36 | <0.001 | 1.26 | 1.17 | 1.34 | <0.001 |
| Q5 (Richest) | 1.43 | 1.33 | 1.53 | <0.001 | 1.36 | 1.26 | 1.46 | <0.001 |
| BMI |  |  |  |  |  |  |  |  |
| <18.5 kg/m2 | 1.11 | 1.06 | 1.17 | <0.001 | 1.03 | 0.93 | 1.13 | 0.590 |
| 18.5-22.9 kg/m2 | 1(Reference) |  |  |  | 1(Reference) |  |  |  |
| 23.0-24.9 kg/m2 | 0.98 | 0.93 | 1.03 | 0.392 | 0.94 | 0.88 | 0.99 | 0.033 |
| 25.0-27.4 kg/m2 | 1.12 | 1.07 | 1.17 | <0.001 | 1.12 | 1.06 | 1.19 | <0.001 |
| 27.5-29.9 kg/m2 | 1.13 | 1.07 | 1.20 | <0.001 | 1.58 | 1.49 | 1.67 | <0.001 |
| 30.0 kg/m2 | 1.50 | 1.42 | 1.59 | <0.001 | 1.79 | 1.69 | 1.89 | <0.001 |
| Tobacco, smoked | 0.93 | 0.88 | 0.98 | 0.009 | 1.05 | 0.99 | 1.11 | 0.124 |
| Tobacco, smokeless | 0.90 | 0.86 | 0.94 | <0.001 | 0.83 | 0.78 | 0.88 | <0.001 |
| Currently married | 1.07 | 1.02 | 1.13 | 0.009 | 1.25 | 1.18 | 1.33 | <0.001 |
| Female | 1.75 | 1.68 | 1.82 | <0.001 | 1.54 | 1.48 | 1.60 | <0.001 |

Abbreviations: CI = Confidence Interval; BMI = Body Mass Index; Q = Quintile

^10^ These covariate-adjusted poisson regressions contained all predictor variables listed in the table (age group, wealth quintile, education, BMI, tobacco consumption, marital status, and sex) and a binary indicator for each district (district-level fixed effects) as explanatory variables. Sampling weights to adjust for the complex survey design and for the differing number of women and men were used. Standard errors were adjusted for clustering at the level of the primary sampling unit.

Controlled

|  | **Rural** |  |  |  | **Urban** |  |  |  |
| --- | --- | --- | --- | --- | --- | --- | --- | --- |
|  | **Relative Risk** | **95% CI (low)** | **95% CI (high)** | **P Value** | **Relative Risk** | **95% CI (low)** | **95% CI (high)** | **P Value** |
| Age group, y |  |  |  |  |  |  |  |  |
| 15-19 | 1(Reference) |  |  |  | 1(Reference) |  |  |  |
| 20-24 | 0.67 | 0.61 | 0.74 | 0.013 | 1.16 | 1.00 | 1.35 | 0.044 |
| 25-29 | 0.70 | 0.64 | 0.78 | 0.028 | 0.88 | 0.76 | 1.02 | 0.098 |
| 30-34 | 0.69 | 0.62 | 0.76 | 0.002 | 0.82 | 0.71 | 0.96 | 0.012 |
| 35-40 | 0.61 | 0.55 | 0.67 | <0.001 | 0.80 | 0.69 | 0.93 | 0.004 |
| 40-44 | 0.63 | 0.57 | 0.70 | <0.001 | 1.21 | 1.05 | 1.40 | 0.011 |
| 45-49 | 0.76 | 0.69 | 0.83 | <0.001 | 1.32 | 1.14 | 1.53 | <0.001 |
| Education |  |  |  |  |  |  |  |  |
| Primary school unfinished | 1(Reference) |  |  |  | 1(Reference) |  |  |  |
| Primary school finished | 0.99 | 0.91 | 1.07 | 0.785 | 1.03 | 0.93 | 1.14 | 0.572 |
| Secondary school unfinished | 1.04 | 0.99 | 1.10 | 0.105 | 0.99 | 0.92 | 1.06 | 0.691 |
| Secondary school or above | 1.13 | 1.05 | 1.21 | 0.001 | 1.00 | 0.93 | 1.08 | 0.951 |
| Household wealth quintile |  |  |  |  |  |  |  |  |
| Q1 (Poorest) | 1(Reference) |  |  |  | 1(Reference) |  |  |  |
| Q2 | 1.09 | 1.00 | 1.17 | 0.038 | 1.07 | 0.99 | 1.17 | 0.102 |
| Q3 | 1.17 | 1.08 | 1.26 | <0.001 | 1.00 | 0.91 | 1.09 | 0.980 |
| Q4 | 1.16 | 1.07 | 1.26 | <0.001 | 1.42 | 1.30 | 1.55 | <0.001 |
| Q5 (Richest) | 1.29 | 1.19 | 1.41 | <0.001 | 1.47 | 1.34 | 1.62 | <0.001 |
| BMI |  |  |  |  |  |  |  |  |
| <18.5 kg/m2 | 1.20 | 1.13 | 1.27 | <0.001 | 1.13 | 1.02 | 1.26 | 0.019 |
| 18.5-22.9 kg/m2 | 1(Reference) |  |  |  | 1(Reference) |  |  |  |
| 23.0-24.9 kg/m2 | 0.87 | 0.82 | 0.92 | <0.001 | 0.82 | 0.76 | 0.89 | <0.001 |
| 25.0-27.4 kg/m2 | 0.81 | 0.76 | 0.86 | <0.001 | 0.87 | 0.81 | 0.94 | <0.001 |
| 27.5-29.9 kg/m2 | 0.79 | 0.72 | 0.85 | <0.001 | 1.25 | 1.16 | 1.34 | <0.001 |
| 30.0 kg/m2 | 1.02 | 0.94 | 1.10 | 0.639 | 1.29 | 1.20 | 1.39 | <0.001 |
| Tobacco, smoked | 0.86 | 0.79 | 0.92 | <0.001 | 0.97 | 0.89 | 1.06 | 0.536 |
| Tobacco, smokeless | 0.83 | 0.78 | 0.89 | <0.001 | 0.83 | 0.77 | 0.91 | <0.001 |
| Currently married | 1.05 | 0.99 | 1.12 | 0.115 | 1.21 | 1.12 | 1.30 | <0.001 |
| Female | 1.91 | 1.82 | 2.00 | <0.001 | 1.87 | 1.77 | 1.97 | <0.001 |

Abbreviations: CI = Confidence Interval; BMI = Body Mass Index; Q = Quintile

^10^ These covariate-adjusted poisson regressions contained all predictor variables listed in the table (age group, wealth quintile, education, BMI, tobacco consumption, marital status, and sex) and a binary indicator for each district (district-level fixed effects) as explanatory variables. Sampling weights to adjust for the complex survey design and for the differing number of women and men were used. Standard errors were adjusted for clustering at the level of the primary sampling unit.

# Table Z. Predictors of reaching each cascade step using covariate-adjusted poisson regressions among men only^11^

Screened

|  | Screened |  |  |  |  |  |  |  |
| --- | --- | --- | --- | --- | --- | --- | --- | --- |
|  | Rural |  |  |  | Urban |  |  |  |
|  | Relative Risk | 95% CI (low) | 95% CI (high) | P Value | Relative Risk | 95% CI (low) | 95% CI (high) | P Value |
| Age group, y |  |  |  |  |  |  |  |  |
| 15-19 | 1(Reference) |  |  |  | 1(Reference) |  |  |  |
| 20-24 | 1.13 | 1.03 | 1.23 | 0.007 | 1.13 | 1.02 | 1.24 | 0.021 |
| 25-29 | 1.28 | 1.17 | 1.39 | <0.001 | 1.15 | 1.04 | 1.27 | 0.007 |
| 30-34 | 1.32 | 1.21 | 1.44 | <0.001 | 1.20 | 1.08 | 1.33 | 0.001 |
| 35-40 | 1.31 | 1.20 | 1.44 | <0.001 | 1.25 | 1.13 | 1.38 | <0.001 |
| 40-44 | 1.38 | 1.26 | 1.51 | <0.001 | 1.30 | 1.17 | 1.44 | <0.001 |
| 45-49 | 1.40 | 1.28 | 1.53 | <0.001 | 1.34 | 1.21 | 1.48 | <0.001 |
| Education |  |  |  |  |  |  |  |  |
| Primary school unfinished | 1(Reference) |  |  |  | 1(Reference) |  |  |  |
| Primary school finished | 1.04 | 0.98 | 1.10 | 0.213 | 1.04 | 0.96 | 1.12 | 0.340 |
| Secondary school unfinished | 1.09 | 1.05 | 1.14 | <0.001 | 1.06 | 1.00 | 1.12 | 0.032 |
| Secondary school or above | 1.14 | 1.09 | 1.19 | <0.001 | 1.17 | 1.11 | 1.24 | <0.001 |
| Household wealth quintile |  |  |  |  |  |  |  |  |
| Q1 (Poorest) | 1(Reference) |  |  |  | 1(Reference) |  |  |  |
| Q2 | 1.12 | 1.05 | 1.20 | 0.001 | 1.10 | 1.04 | 1.16 | 0.001 |
| Q3 | 1.19 | 1.11 | 1.28 | <0.001 | 1.09 | 1.03 | 1.16 | 0.003 |
| Q4 | 1.24 | 1.15 | 1.33 | <0.001 | 1.14 | 1.07 | 1.21 | <0.001 |
| Q5 (Richest) | 1.28 | 1.19 | 1.37 | <0.001 | 1.19 | 1.13 | 1.27 | <0.001 |
| BMI |  |  |  |  |  |  |  |  |
| <18.5 kg/m2 | 0.99 | 0.94 | 1.04 | 0.743 | 0.99 | 0.91 | 1.07 | 0.71 |
| 18.5-22.9 kg/m2 | 1(Reference) |  |  |  | 1(Reference) |  |  |  |
| 23.0-24.9 kg/m2 | 1.02 | 0.98 | 1.05 | 0.359 | 1.03 | 0.99 | 1.07 | 0.177 |
| 25.0-27.4 kg/m2 | 1.02 | 0.98 | 1.05 | 0.342 | 1.00 | 0.96 | 1.04 | 0.914 |
| 27.5-29.9 kg/m2 | 1.04 | 1.00 | 1.09 | 0.072 | 1.03 | 0.98 | 1.08 | 0.184 |
| 30.0 kg/m2 | 1.07 | 1.02 | 1.13 | 0.011 | 1.08 | 1.03 | 1.13 | 0.003 |
| Tobacco, smoked | 0.98 | 0.95 | 1.01 | 0.122 | 0.98 | 0.95 | 1.01 | 0.266 |
| Tobacco, smokeless | 0.98 | 0.95 | 1.01 | 0.281 | 0.99 | 0.95 | 1.02 | 0.459 |
| Currently married | 1.07 | 1.03 | 1.12 | <0.001 | 1.08 | 1.02 | 1.13 | 0.003 |

Abbreviations: CI = Confidence Interval; BMI = Body Mass Index; Q = Quintil

^11^ These covariate-adjusted poisson regressions contained all predictor variables listed in the table (age group, wealth quintile, education, BMI, tobacco consumption, and marital status) and a binary indicator for each district (district-level fixed effects) as explanatory variables. Sampling weights were not used. Standard errors were adjusted for clustering at the level of the primary sampling unit.

Aware

|  | Aware |  |  |  |  |  |  |  |
| --- | --- | --- | --- | --- | --- | --- | --- | --- |
|  | Rural |  |  |  | Urban |  |  |  |
|  | Relative Risk | 95% CI (low) | 95% CI (high) | P Value | Relative Risk | 95% CI (low) | 95% CI (high) | P Value |
| Age group, y |  |  |  |  |  |  |  |  |
| 15-19 | 1(Reference) |  |  |  | 1(Reference) |  |  |  |
| 20-24 | 0.94 | 0.83 | 1.06 | 0.333 | 0.92 | 0.78 | 1.08 | 0.310 |
| 25-29 | 1.01 | 0.89 | 1.14 | 0.926 | 1.01 | 0.86 | 1.18 | 0.929 |
| 30-34 | 1.00 | 0.87 | 1.14 | 0.968 | 1.04 | 0.87 | 1.23 | 0.694 |
| 35-40 | 1.01 | 0.88 | 1.16 | 0.843 | 1.01 | 0.84 | 1.20 | 0.948 |
| 40-44 | 1.07 | 0.93 | 1.23 | 0.347 | 1.15 | 0.96 | 1.37 | 0.121 |
| 45-49 | 1.11 | 0.97 | 1.27 | 0.143 | 1.27 | 1.07 | 1.51 | 0.006 |
| Education |  |  |  |  |  |  |  |  |
| Primary school unfinished | 1(Reference) |  |  |  | 1(Reference) |  |  |  |
| Primary school finished | 1.06 | 0.94 | 1.19 | 0.338 | 1.00 | 0.85 | 1.17 | 0.972 |
| Secondary school unfinished | 1.10 | 1.02 | 1.18 | 0.012 | 1.08 | 0.97 | 1.21 | 0.161 |
| Secondary school or above | 1.16 | 1.06 | 1.27 | 0.001 | 1.23 | 1.09 | 1.39 | 0.001 |
| Household wealth quintile |  |  |  |  |  |  |  |  |
| Q1 (Poorest) | 1(Reference) |  |  |  | 1(Reference) |  |  |  |
| Q2 | 1.13 | 1.00 | 1.27 | 0.044 | 1.11 | 0.99 | 1.24 | 0.063 |
| Q3 | 1.18 | 1.05 | 1.33 | 0.006 | 1.16 | 1.03 | 1.31 | 0.011 |
| Q4 | 1.23 | 1.09 | 1.39 | 0.001 | 1.15 | 1.02 | 1.31 | 0.025 |
| Q5 (Richest) | 1.25 | 1.10 | 1.42 | 0.001 | 1.20 | 1.05 | 1.37 | 0.006 |
| BMI |  |  |  |  |  |  |  |  |
| <18.5 kg/m2 | 1.17 | 1.08 | 1.26 | <0.001 | 1.11 | 0.96 | 1.28 | 0.142 |
| 18.5-22.9 kg/m2 | 1(Reference) |  |  |  | 1(Reference) |  |  |  |
| 23.0-24.9 kg/m2 | 0.95 | 0.88 | 1.01 | 0.109 | 0.91 | 0.83 | 0.99 | 0.030 |
| 25.0-27.4 kg/m2 | 0.92 | 0.85 | 0.99 | 0.031 | 0.93 | 0.85 | 1.01 | 0.090 |
| 27.5-29.9 kg/m2 | 0.90 | 0.81 | 1.01 | 0.063 | 1.04 | 0.93 | 1.15 | 0.507 |
| 30.0 kg/m2 | 1.11 | 0.98 | 1.25 | 0.116 | 1.11 | 0.98 | 1.25 | 0.096 |
| Tobacco, smoked | 1.04 | 0.98 | 1.10 | 0.172 | 1.03 | 0.95 | 1.10 | 0.514 |
| Tobacco, smokeless | 0.96 | 0.89 | 1.02 | 0.191 | 0.88 | 0.81 | 0.96 | 0.004 |
| Currently married | 1.06 | 0.97 | 1.15 | 0.195 | 1.02 | 0.92 | 1.13 | 0.672 |

Abbreviations: CI = Confidence Interval; BMI = Body Mass Index; Q = Quintile

^11^ These covariate-adjusted poisson regressions contained all predictor variables listed in the table (age group, wealth quintile, education, BMI, tobacco consumption, and marital status) and a binary indicator for each district (district-level fixed effects) as explanatory variables. Sampling weights were not used. Standard errors were adjusted for clustering at the level of the primary sampling unit.

Treated

|  | Rural | | | | Urban | | | |
| --- | --- | --- | --- | --- | --- | --- | --- | --- |
|  | Relative Risk | 95% CI (low) | 95% CI (high) | P Value | Relative Risk | 95% CI (low) | 95% CI (high) | P Value |
| Age group, y |  |  |  |  |  |  |  |  |
| 15-19 | 1(Reference) |  |  |  | 1(Reference) |  |  |  |
| 20-24 | 0.61 | 0.43 | 0.87 | 0.006 | 1.07 | 0.64 | 1.79 | 0.784 |
| 25-29 | 0.75 | 0.54 | 1.05 | 0.099 | 0.93 | 0.55 | 1.57 | 0.773 |
| 30-34 | 0.75 | 0.52 | 1.07 | 0.113 | 0.96 | 0.56 | 1.65 | 0.889 |
| 35-40 | 0.85 | 0.60 | 1.22 | 0.381 | 1.05 | 0.61 | 1.82 | 0.865 |
| 40-44 | 1.09 | 0.77 | 1.54 | 0.630 | 1.94 | 1.13 | 3.30 | 0.015 |
| 45-49 | 1.24 | 0.88 | 1.76 | 0.220 | 2.25 | 1.32 | 3.84 | 0.003 |
| Education |  |  |  |  |  |  |  |  |
| Primary school unfinished | 1(Reference) |  |  |  | 1(Reference) |  |  |  |
| Primary school finished | 0.96 | 0.72 | 1.27 | 0.771 | 1.01 | 0.69 | 1.47 | 0.971 |
| Secondary school unfinished | 1.01 | 0.85 | 1.20 | 0.902 | 0.97 | 0.75 | 1.26 | 0.830 |
| Secondary school or above | 1.23 | 1.00 | 1.50 | 0.045 | 1.16 | 0.88 | 1.52 | 0.291 |
| Household wealth quintile |  |  |  |  |  |  |  |  |
| Q1 (Poorest) | 1(Reference) |  |  |  | 1(Reference) |  |  |  |
| Q2 | 1.03 | 0.78 | 1.37 | 0.828 | 1.46 | 1.09 | 1.94 | 0.010 |
| Q3 | 1.23 | 0.93 | 1.62 | 0.139 | 1.41 | 1.05 | 1.89 | 0.022 |
| Q4 | 1.23 | 0.92 | 1.64 | 0.156 | 1.36 | 1.00 | 1.85 | 0.053 |
| Q5 (Richest) | 1.41 | 1.05 | 1.88 | 0.022 | 1.65 | 1.19 | 2.27 | 0.002 |
| BMI |  |  |  |  |  |  |  |  |
| <18.5 kg/m2 | 1.29 | 1.04 | 1.60 | 0.023 | 1.14 | 0.76 | 1.70 | 0.532 |
| 18.5-22.9 kg/m2 | 1(Reference) |  |  |  | 1 (Reference) |  |  |  |
| 23.0-24.9 kg/m2 | 1.06 | 0.90 | 1.25 | 0.497 | 1.07 | 0.85 | 1.36 | 0.549 |
| 25.0-27.4 kg/m2 | 1.07 | 0.90 | 1.28 | 0.437 | 1.17 | 0.93 | 1.47 | 0.190 |
| 27.5-29.9 kg/m2 | 1.05 | 0.83 | 1.32 | 0.677 | 1.67 | 1.32 | 2.12 | <0.001 |
| 30.0 kg/m2 | 1.59 | 1.25 | 2.03 | <0.001 | 1.96 | 1.54 | 2.49 | <0.001 |
| Tobacco, smoked | 0.99 | 0.86 | 1.14 | 0.889 | 1.04 | 0.87 | 1.23 | 0.678 |
| Tobacco, smokeless | 0.93 | 0.80 | 1.09 | 0.377 | 0.80 | 0.66 | 0.96 | 0.018 |
| Currently married | 1.18 | 0.94 | 1.48 | 0.149 | 1.20 | 0.90 | 1.61 | 0.216 |

Abbreviations: CI = Confidence Interval; BMI = Body Mass Index; Q = Quintile

^11^ These covariate-adjusted poisson regressions contained all predictor variables listed in the table (age group, wealth quintile, education, BMI, tobacco consumption, and marital status) and a binary indicator for each district (district-level fixed effects) as explanatory variables. Sampling weights were not used. Standard errors were adjusted for clustering at the level of the primary sampling unit.

Controlled

|  | Rural | | | | Urban | | | |
| --- | --- | --- | --- | --- | --- | --- | --- | --- |
|  | Relative Risk | 95% CI (low) | 95% CI (high) | P Value | Relative Risk | 95% CI (low) | 95% CI (high) | P Value |
| Age group, y |  |  |  |  |  |  |  |  |
| 15-19 | 1(Reference) |  |  |  | 1(Reference) |  |  |  |
| 20-24 | 0.61 | 0.43 | 0.88 | 0.008 | 1.18 | 0.67 | 2.07 | 0.572 |
| 25-29 | 0.68 | 0.47 | 1.00 | 0.049 | 1.09 | 0.60 | 1.96 | 0.778 |
| 30-34 | 0.60 | 0.39 | 0.91 | 0.017 | 0.73 | 0.39 | 1.39 | 0.345 |
| 35-40 | 0.65 | 0.43 | 0.98 | 0.038 | 0.58 | 0.30 | 1.13 | 0.107 |
| 40-44 | 0.66 | 0.44 | 1.01 | 0.054 | 1.32 | 0.69 | 2.50 | 0.399 |
| 45-49 | 0.80 | 0.53 | 1.21 | 0.292 | 1.38 | 0.73 | 2.62 | 0.327 |
| Education |  |  |  |  |  |  |  |  |
| Primary school unfinished | 1(Reference) |  |  |  | 1(Reference) |  |  |  |
| Primary school finished | 1.00 | 0.66 | 1.53 | 0.985 | 0.96 | 0.54 | 1.71 | 0.881 |
| Secondary school unfinished | 1.12 | 0.87 | 1.44 | 0.380 | 0.94 | 0.62 | 1.40 | 0.746 |
| Secondary school or above | 1.26 | 0.95 | 1.68 | 0.109 | 1.14 | 0.76 | 1.73 | 0.525 |
| Household wealth quintile |  |  |  |  |  |  |  |  |
| Q1 (Poorest) | 1(Reference) |  |  |  | 1(Reference) |  |  |  |
| Q2 | 1.03 | 0.72 | 1.48 | 0.878 | 1.58 | 1.01 | 2.47 | 0.045 |
| Q3 | 1.19 | 0.84 | 1.70 | 0.329 | 1.38 | 0.87 | 2.17 | 0.167 |
| Q4 | 1.16 | 0.80 | 1.66 | 0.437 | 1.47 | 0.92 | 2.36 | 0.109 |
| Q5 (Richest) | 1.43 | 0.99 | 2.07 | 0.058 | 1.67 | 1.02 | 2.72 | 0.041 |
| BMI |  |  |  |  |  |  |  |  |
| <18.5 kg/m2 | 1.46 | 1.14 | 1.87 | 0.003 | 1.58 | 1.01 | 2.46 | 0.044 |
| 18.5-22.9 kg/m2 | 1(Reference) |  |  |  | 1(Reference) |  |  |  |
| 23.0-24.9 kg/m2 | 0.93 | 0.74 | 1.17 | 0.534 | 1.05 | 0.76 | 1.46 | 0.765 |
| 25.0-27.4 kg/m2 | 0.78 | 0.60 | 1.01 | 0.056 | 0.95 | 0.68 | 1.32 | 0.770 |
| 27.5-29.9 kg/m2 | 0.57 | 0.39 | 0.83 | 0.003 | 1.65 | 1.16 | 2.34 | 0.005 |
| 30.0 kg/m2 | 1.17 | 0.81 | 1.69 | 0.406 | 1.91 | 1.31 | 2.78 | 0.001 |
| Tobacco, smoked | 1.03 | 0.85 | 1.25 | 0.762 | 1.02 | 0.80 | 1.31 | 0.854 |
| Tobacco, smokeless | 0.87 | 0.71 | 1.07 | 0.176 | 0.70 | 0.53 | 0.93 | 0.014 |
| Currently married | 1.08 | 0.81 | 1.44 | 0.594 | 1.26 | 0.86 | 1.84 | 0.235 |

Abbreviations: CI = Confidence Interval; BMI = Body Mass Index; Q = Quintile

^11^ These covariate-adjusted poisson regressions contained all predictor variables listed in the table (age group, wealth quintile, education, BMI, tobacco consumption, and marital status) and a binary indicator for each district (district-level fixed effects) as explanatory variables. Sampling weights were not used. Standard errors were adjusted for clustering at the level of the primary sampling unit.

# Table AA. Predictors of reaching each cascade step using covariate-adjusted poisson regressions among women only^12^

Screened

|  | Rural | | | | Urban | | | |
| --- | --- | --- | --- | --- | --- | --- | --- | --- |
|  | Relative Risk | 95% CI (low) | 95% CI (high) | P Value | Relative Risk | 95% CI (low) | 95% CI (high) | P Value |
| Age group, y |  |  |  |  |  |  |  |  |
| 15-19 | 1(Reference) |  |  |  | 1(Reference) |  |  |  |
| 20-24 | 1.16 | 1.13 | 1.20 | <0.001 | 1.13 | 1.09 | 1.17 | <0.001 |
| 25-29 | 1.20 | 1.17 | 1.23 | <0.001 | 1.15 | 1.11 | 1.19 | <0.001 |
| 30-34 | 1.18 | 1.15 | 1.21 | <0.001 | 1.15 | 1.11 | 1.19 | <0.001 |
| 35-40 | 1.15 | 1.12 | 1.18 | <0.001 | 1.15 | 1.11 | 1.19 | <0.001 |
| 40-44 | 1.15 | 1.12 | 1.18 | <0.001 | 1.14 | 1.11 | 1.18 | <0.001 |
| 45-49 | 1.14 | 1.11 | 1.17 | <0.001 | 1.16 | 1.12 | 1.19 | <0.001 |
| Education |  |  |  |  |  |  |  |  |
| Primary school unfinished | 1(Reference) |  |  |  | 1(Reference) |  |  |  |
| Primary school finished | 1.05 | 1.04 | 1.06 | <0.001 | 1.03 | 1.01 | 1.05 | <0.001 |
| Secondary school unfinished | 1.07 | 1.06 | 1.08 | <0.001 | 1.04 | 1.03 | 1.05 | <0.001 |
| Secondary school or above | 1.09 | 1.07 | 1.10 | <0.001 | 1.06 | 1.05 | 1.07 | <0.001 |
| Household wealth quintile |  |  |  |  |  |  |  |  |
| Q1 (Poorest) | 1(Reference) |  |  |  | 1(Reference) |  |  |  |
| Q2 | 1.13 | 1.11 | 1.15 | <0.001 | 1.03 | 1.02 | 1.05 | <0.001 |
| Q3 | 1.18 | 1.16 | 1.20 | <0.001 | 1.04 | 1.03 | 1.06 | <0.001 |
| Q4 | 1.22 | 1.19 | 1.24 | <0.001 | 1.05 | 1.03 | 1.06 | <0.001 |
| Q5 (Richest) | 1.24 | 1.21 | 1.26 | <0.001 | 1.06 | 1.05 | 1.08 | <0.001 |
| BMI |  |  |  |  |  |  |  |  |
| <18.5 kg/m2 | 1.02 | 1.01 | 1.03 | 0.005 | 1.00 | 0.98 | 1.02 | 0.819 |
| 18.5-22.9 kg/m2 | 1(Reference) |  |  |  | 1(Reference) |  |  |  |
| 23.0-24.9 kg/m2 | 1.01 | 1.00 | 1.02 | 0.053 | 1.00 | 0.99 | 1.01 | 0.660 |
| 25.0-27.4 kg/m2 | 1.02 | 1.00 | 1.03 | 0.004 | 1.00 | 0.99 | 1.01 | 0.975 |
| 27.5-29.9 kg/m2 | 1.03 | 1.02 | 1.04 | <0.001 | 1.00 | 0.98 | 1.01 | 0.523 |
| 30.0 kg/m2 | 1.05 | 1.04 | 1.06 | <0.001 | 1.02 | 1.01 | 1.03 | 0.003 |
| Tobacco, smoked | 0.99 | 0.97 | 1.02 | 0.553 | 0.99 | 0.96 | 1.02 | 0.476 |
| Tobacco, smokeless | 0.99 | 0.98 | 1.01 | 0.385 | 1.00 | 0.98 | 1.01 | 0.625 |
| Currently married | 1.12 | 1.10 | 1.13 | <0.001 | 1.08 | 1.07 | 1.09 | <0.001 |

Abbreviations: CI = Confidence Interval; BMI = Body Mass Index; Q = Quintile

^12^ These covariate-adjusted poisson regressions contained all predictor variables listed in the table (age group, wealth quintile, education, BMI, tobacco consumption, and marital status) and a binary indicator for each district (district-level fixed effects) as explanatory variables. Sampling weights were not used. Standard errors were adjusted for clustering at the level of the primary sampling unit.

Aware

|  | Rural | | | | Urban | | | |
| --- | --- | --- | --- | --- | --- | --- | --- | --- |
|  | Relative Risk | 95% CI (low) | 95% CI (high) | P Value | Relative Risk | 95% CI (low) | 95% CI (high) | P Value |
| Age group, y |  |  |  |  |  |  |  |  |
| 15-19 | 1(Reference) |  |  |  | 1(Reference) |  |  |  |
| 20-24 | 1.09 | 1.05 | 1.12 | <0.001 | 1.06 | 1.01 | 1.11 | 0.020 |
| 25-29 | 1.05 | 1.02 | 1.09 | 0.005 | 0.99 | 0.94 | 1.03 | 0.590 |
| 30-34 | 0.99 | 0.95 | 1.03 | 0.565 | 0.94 | 0.89 | 0.98 | 0.009 |
| 35-40 | 0.92 | 0.89 | 0.96 | <0.001 | 0.89 | 0.85 | 0.93 | <0.001 |
| 40-44 | 0.91 | 0.88 | 0.95 | <0.001 | 0.89 | 0.85 | 0.94 | <0.001 |
| 45-49 | 0.93 | 0.90 | 0.97 | 0.001 | 0.95 | 0.90 | 1.00 | 0.038 |
| Education |  |  |  |  |  |  |  |  |
| Primary school unfinished | 1(Reference) |  |  |  | 1(Reference) |  |  |  |
| Primary school finished | 1.08 | 1.06 | 1.26 | <0.001 | 1.04 | 1.00 | 1.08 | 0.038 |
| Secondary school unfinished | 1.12 | 1.10 | 1.38 | <0.001 | 1.07 | 1.05 | 1.10 | <0.001 |
| Secondary school or above | 1.15 | 1.12 | 1.53 | <0.001 | 1.10 | 1.07 | 1.13 | <0.001 |
| Household wealth quintile |  |  |  |  |  |  |  |  |
| Q1 (Poorest) | 1(Reference) |  |  |  | 1(Reference) |  |  |  |
| Q2 | 1.19 | 1.15 | 1.23 | <0.001 | 1.03 | 1.00 | 1.06 | 0.049 |
| Q3 | 1.27 | 1.23 | 1.31 | <0.001 | 1.06 | 1.02 | 1.09 | 0.001 |
| Q4 | 1.33 | 1.29 | 1.38 | <0.001 | 1.07 | 1.03 | 1.11 | <0.001 |
| Q5 (Richest) | 1.36 | 1.31 | 1.41 | <0.001 | 1.10 | 1.06 | 1.14 | <0.001 |
| BMI |  |  |  |  |  |  |  |  |
| <18.5 kg/m2 | 1.07 | 1.05 | 1.09 | <0.001 | 1.07 | 1.03 | 1.10 | <0.001 |
| 18.5-22.9 kg/m2 | 1(Reference) |  |  |  | 1(Reference) |  |  |  |
| 23.0-24.9 kg/m2 | 0.94 | 0.92 | 0.96 | <0.001 | 0.95 | 0.93 | 0.98 | <0.001 |
| 25.0-27.4 kg/m2 | 0.93 | 0.91 | 0.95 | <0.001 | 0.94 | 0.92 | 0.97 | <0.001 |
| 27.5-29.9 kg/m2 | 0.96 | 0.93 | 0.98 | 0.002 | 0.96 | 0.93 | 0.99 | 0.007 |
| 30.0 kg/m2 | 1.00 | 0.97 | 1.03 | 0.910 | 1.02 | 0.99 | 1.05 | 0.225 |
| Tobacco, smoked | 1.02 | 0.97 | 1.07 | 0.511 | 1.03 | 0.96 | 1.10 | 0.444 |
| Tobacco, smokeless | 0.96 | 0.93 | 0.99 | 0.007 | 1.02 | 0.99 | 1.06 | 0.205 |
| Currently married | 1.15 | 1.12 | 1.17 | <0.001 | 1.14 | 1.11 | 1.17 | <0.001 |

Abbreviations: CI = Confidence Interval; BMI = Body Mass Index; Q = Quintile

^12^ These covariate-adjusted poisson regressions contained all predictor variables listed in the table (age group, wealth quintile, education, BMI, tobacco consumption, and marital status) and a binary indicator for each district (district-level fixed effects) as explanatory variables. Sampling weights were not used. Standard errors were adjusted for clustering at the level of the primary sampling unit.

Treated

|  | Rural | | | | Urban | | | |
| --- | --- | --- | --- | --- | --- | --- | --- | --- |
|  | Relative Risk | 95% CI (low) | 95% CI (high) | P Value | Relative Risk | 95% CI (low) | 95% CI (high) | P Value |
| Age group, y |  |  |  |  |  |  |  |  |
| 15-19 | 1(Reference) |  |  |  | 1(Reference) |  |  |  |
| 20-24 | 0.93 | 0.84 | 1.04 | 0.196 | 0.98 | 0.81 | 1.17 | 0.793 |
| 25-29 | 0.96 | 0.87 | 1.07 | 0.470 | 0.88 | 0.73 | 1.05 | 0.153 |
| 30-34 | 0.99 | 0.89 | 1.10 | 0.901 | 1.01 | 0.85 | 1.20 | 0.895 |
| 35-40 | 1.08 | 0.98 | 1.20 | 0.127 | 1.17 | 0.99 | 1.39 | 0.069 |
| 40-44 | 1.21 | 1.09 | 1.34 | <0.001 | 1.50 | 1.27 | 1.77 | <0.001 |
| 45-49 | 1.40 | 1.26 | 1.55 | <0.001 | 1.90 | 1.61 | 2.24 | <0.001 |
| Education |  |  |  |  |  |  |  |  |
| Primary school unfinished | 1(Reference) |  |  |  | 1(Reference) |  |  |  |
| Primary school finished | 1.02 | 0.95 | 1.09 | 0.582 | 1.06 | 0.97 | 1.16 | 0.205 |
| Secondary school unfinished | 1.04 | 0.99 | 1.08 | 0.113 | 0.99 | 0.93 | 1.05 | 0.718 |
| Secondary school or above | 0.97 | 0.91 | 1.04 | 0.350 | 0.94 | 0.87 | 1.01 | 0.089 |
| Household wealth quintile |  |  |  |  |  |  |  |  |
| Q1 (Poorest) | 1(Reference) |  |  |  | 1(Reference) |  |  |  |
| Q2 | 1.28 | 1.19 | 1.38 | <0.001 | 1.08 | 1.00 | 1.18 | 0.054 |
| Q3 | 1.35 | 1.25 | 1.46 | <0.001 | 1.15 | 1.06 | 1.25 | 0.001 |
| Q4 | 1.46 | 1.35 | 1.58 | <0.001 | 1.26 | 1.15 | 1.37 | <0.001 |
| Q5 (Richest) | 1.55 | 1.42 | 1.68 | <0.001 | 1.32 | 1.20 | 1.45 | <0.001 |
| BMI |  |  |  |  |  |  |  |  |
| <18.5 kg/m2 | 1.09 | 1.03 | 1.15 | 0.002 | 1.07 | 0.96 | 1.20 | 0.239 |
| 18.5-22.9 kg/m2 | 1(Reference) |  |  |  | 1(Reference) |  |  |  |
| 23.0-24.9 kg/m2 | 1.01 | 0.96 | 1.06 | 0.701 | 1.00 | 0.93 | 1.08 | 0.950 |
| 25.0-27.4 kg/m2 | 1.06 | 1.01 | 1.12 | 0.030 | 1.10 | 1.02 | 1.18 | 0.010 |
| 27.5-29.9 kg/m2 | 1.17 | 1.10 | 1.25 | <0.001 | 1.23 | 1.14 | 1.33 | <0.001 |
| 30.0 kg/m2 | 1.44 | 1.36 | 1.53 | <0.001 | 1.57 | 1.47 | 1.68 | <0.001 |
| Tobacco, smoked | 0.93 | 0.83 | 1.04 | 0.226 | 1.06 | 0.90 | 1.24 | 0.469 |
| Tobacco, smokeless | 1.00 | 0.94 | 1.07 | 0.971 | 1.05 | 0.96 | 1.14 | 0.275 |
| Currently married | 1.09 | 1.04 | 1.16 | 0.001 | 1.07 | 1.00 | 1.15 | 0.038 |

Abbreviations: CI = Confidence Interval; BMI = Body Mass Index; Q = Quintile

^12^ These covariate-adjusted poisson regressions contained all predictor variables listed in the table (age group, wealth quintile, education, BMI, tobacco consumption, and marital status) and a binary indicator for each district (district-level fixed effects) as explanatory variables. Sampling weights were not used. Standard errors were adjusted for clustering at the level of the primary sampling unit.

Controlled

|  | Rural | | | | Urban | | | |
| --- | --- | --- | --- | --- | --- | --- | --- | --- |
|  | Relative Risk | 95% CI (low) | 95% CI (high) | P Value | Relative Risk | 95% CI (low) | 95% CI (high) | P Value |
| Age group, y |  |  |  |  |  |  |  |  |
| 15-19 | 1(Reference) |  |  |  | 1(Reference) |  |  |  |
| 20-24 | 0.91 | 0.82 | 1.02 | 0.118 | 0.97 | 0.80 | 1.19 | 0.784 |
| 25-29 | 0.92 | 0.82 | 1.03 | 0.151 | 0.79 | 0.65 | 0.97 | 0.026 |
| 30-34 | 0.88 | 0.78 | 0.99 | 0.029 | 0.81 | 0.67 | 0.99 | 0.044 |
| 35-40 | 0.81 | 0.72 | 0.91 | <0.001 | 0.79 | 0.64 | 0.96 | 0.018 |
| 40-44 | 0.78 | 0.70 | 0.88 | <0.001 | 0.92 | 0.76 | 1.11 | 0.380 |
| 45-49 | 0.80 | 0.71 | 0.90 | <0.001 | 1.12 | 0.92 | 1.36 | 0.248 |
| Education |  |  |  |  |  |  |  |  |
| Primary school unfinished | 1(Reference) |  |  |  | 1(Reference) |  |  |  |
| Primary school finished | 1.02 | 0.94 | 1.12 | 0.581 | 1.08 | 0.95 | 1.23 | 0.231 |
| Secondary school unfinished | 1.06 | 1.00 | 1.12 | 0.056 | 0.99 | 0.91 | 1.08 | 0.893 |
| Secondary school or above | 1.05 | 0.96 | 1.14 | 0.282 | 0.95 | 0.86 | 1.05 | 0.331 |
| Household wealth quintile |  |  |  |  |  |  |  |  |
| Q1 (Poorest) | 1(Reference) |  |  |  | 1(Reference) |  |  |  |
| Q2 | 1.23 | 1.12 | 1.34 | <0.001 | 1.12 | 1.00 | 1.25 | 0.048 |
| Q3 | 1.31 | 1.19 | 1.43 | <0.001 | 1.14 | 1.02 | 1.28 | 0.024 |
| Q4 | 1.39 | 1.26 | 1.53 | <0.001 | 1.37 | 1.21 | 1.54 | <0.001 |
| Q5 (Richest) | 1.44 | 1.30 | 1.59 | <0.001 | 1.40 | 1.23 | 1.59 | <0.001 |
| BMI |  |  |  |  |  |  |  |  |
| <18.5 kg/m2 | 1.17 | 1.10 | 1.25 | <0.001 | 1.11 | 0.97 | 1.27 | 0.120 |
| 18.5-22.9 kg/m2 | 1(Reference) |  |  |  | 1(Reference) |  |  |  |
| 23.0-24.9 kg/m2 | 0.85 | 0.80 | 0.91 | <0.001 | 0.89 | 0.81 | 0.98 | 0.024 |
| 25.0-27.4 kg/m2 | 0.82 | 0.77 | 0.89 | <0.001 | 0.90 | 0.82 | 0.99 | 0.027 |
| 27.5-29.9 kg/m2 | 0.88 | 0.81 | 0.96 | 0.004 | 0.93 | 0.84 | 1.03 | 0.183 |
| 30.0 kg/m2 | 1.02 | 0.94 | 1.11 | 0.622 | 1.19 | 1.09 | 1.31 | <0.001 |
| Tobacco, smoked | 1.05 | 0.91 | 1.21 | 0.534 | 0.99 | 0.78 | 1.25 | 0.911 |
| Tobacco, smokeless | 0.95 | 0.87 | 1.03 | 0.235 | 1.08 | 0.95 | 1.23 | 0.214 |
| Currently married | 1.11 | 1.04 | 1.20 | 0.003 | 1.12 | 1.02 | 1.23 | 0.019 |

Abbreviations: CI = Confidence Interval; BMI = Body Mass Index; Q = Quintile

^12^ These covariate-adjusted poisson regressions contained all predictor variables listed in the table (age group, wealth quintile, education, BMI, tobacco consumption, and marital status) and a binary indicator for each district (district-level fixed effects) as explanatory variables. Sampling weights were not used. Standard errors were adjusted for clustering at the level of the primary sampling unit.

# Table AB. Predictors of reaching each cascade step using covariate-unadjusted poisson regressions^13^

Screened

|  | Rural | | | | Urban | | | |
| --- | --- | --- | --- | --- | --- | --- | --- | --- |
|  | Relative Risk | 95% CI (low) | 95% CI (high) | P Value | Relative Risk | 95% CI (low) | 95% CI (high) | P Value |
| Age group, y |  |  |  |  |  |  |  |  |
| 15-19 | 1 (Reference) |  |  |  | 1 (Reference) |  |  |  |
| 20-24 | 1.23 | 1.20 | 1.26 | <0.001 | 1.17 | 1.14 | 1.21 | <0.001 |
| 25-29 | 1.30 | 1.27 | 1.33 | <0.001 | 1.22 | 1.18 | 1.26 | <0.001 |
| 30-34 | 1.28 | 1.25 | 1.31 | <0.001 | 1.24 | 1.20 | 1.28 | <0.001 |
| 35-40 | 1.24 | 1.21 | 1.27 | <0.001 | 1.24 | 1.20 | 1.28 | <0.001 |
| 40-44 | 1.24 | 1.21 | 1.27 | <0.001 | 1.24 | 1.20 | 1.28 | <0.001 |
| 45-49 | 1.23 | 1.20 | 1.26 | <0.001 | 1.25 | 1.21 | 1.29 | <0.001 |
| Education |  |  |  |  |  |  |  |  |
| Primary school unfinished | 1 (Reference) |  |  |  | 1 (Reference) |  |  |  |
| Primary school finished | 1.06 | 1.05 | 1.08 | <0.001 | 1.03 | 1.01 | 1.05 | <0.001 |
| Secondary school unfinished | 1.07 | 1.06 | 1.08 | <0.001 | 1.03 | 1.02 | 1.04 | <0.001 |
| Secondary school or above | 1.08 | 1.07 | 1.09 | <0.001 | 1.06 | 1.05 | 1.07 | <0.001 |
| Household wealth quintile |  |  |  |  |  |  |  |  |
| Q1 (Poorest) | 1 (Reference) |  |  |  | 1 (Reference) |  |  |  |
| Q2 | 1.14 | 1.12 | 1.16 | <0.001 | 1.06 | 1.04 | 1.07 | <0.001 |
| Q3 | 1.21 | 1.19 | 1.23 | <0.001 | 1.08 | 1.06 | 1.10 | <0.001 |
| Q4 | 1.27 | 1.24 | 1.29 | <0.001 | 1.10 | 1.09 | 1.12 | <0.001 |
| Q5 (Richest) | 1.32 | 1.29 | 1.34 | <0.001 | 1.13 | 1.12 | 1.15 | <0.001 |
| BMI |  |  |  |  |  |  |  |  |
| <18.5 kg/m2 | 1.00 | 0.98 | 1.01 | 0.460 | 0.97 | 0.95 | 0.99 | 0.001 |
| 18.5-22.9 kg/m2 | 1 (Reference) |  |  |  | 1 (Reference) |  |  |  |
| 23.0-24.9 kg/m2 | 1.03 | 1.02 | 1.04 | <0.001 | 1.02 | 1.01 | 1.04 | <0.001 |
| 25.0-27.4 kg/m2 | 1.06 | 1.04 | 1.07 | <0.001 | 1.03 | 1.02 | 1.04 | <0.001 |
| 27.5-29.9 kg/m2 | 1.08 | 1.07 | 1.10 | <0.001 | 1.04 | 1.03 | 1.06 | <0.001 |
| 30.0 kg/m2 | 1.11 | 1.10 | 1.13 | <0.001 | 1.08 | 1.07 | 1.09 | <0.001 |
| Tobacco, smoked | 0.87 | 0.85 | 0.88 | <0.001 | 0.88 | 0.86 | 0.90 | <0.001 |
| Tobacco, smokeless | 0.91 | 0.90 | 0.92 | <0.001 | 0.92 | 0.90 | 0.93 | <0.001 |
| Currently married | 1.18 | 1.16 | 1.19 | <0.001 | 1.13 | 1.12 | 1.15 | <0.001 |
| Female | 1.23 | 1.22 | 1.25 | <0.001 | 1.19 | 1.18 | 1.21 | <0.001 |

Abbreviations: CI = Confidence Interval; Q = Quintile

^13^ These covariate-unadjusted regressions contained one of the listed indicators in the table (age group, wealth quintile, education, BMI, Tobacco smoked, Tobacco smokeless, marital status, sex) and a binary indicator for each district (district-level fixed effects) as explanatory variables. Standard errors were adjusted for clustering at the level of the primary sampling unit. Sampling weights were not used.

Aware

|  | Rural | | | | Urban | | | |
| --- | --- | --- | --- | --- | --- | --- | --- | --- |
|  | Relative Risk | 95% CI (low) | 95% CI (high) | P Value | Relative Risk | 95% CI (low) | 95% CI (high) | P Value |
| Age group, y |  |  |  |  |  |  |  |  |
| 15-19 | 1 (Reference) |  |  |  | 1 (Reference) |  |  |  |
| 20-24 | 1.13 | 1.10 | 1.17 | <0.001 | 1.10 | 1.05 | 1.15 | <0.001 |
| 25-29 | 1.11 | 1.07 | 1.14 | <0.001 | 1.05 | 1.01 | 1.10 | 0.014 |
| 30-34 | 1.02 | 0.99 | 1.06 | 0.185 | 1.01 | 0.97 | 1.05 | 0.624 |
| 35-40 | 0.95 | 0.92 | 0.98 | 0.002 | 0.96 | 0.92 | 1.00 | 0.057 |
| 40-44 | 0.93 | 0.90 | 0.97 | <0.001 | 0.98 | 0.94 | 1.02 | 0.259 |
| 45-49 | 0.95 | 0.92 | 0.98 | 0.001 | 1.04 | 0.99 | 1.08 | 0.112 |
| Education |  |  |  |  |  |  |  |  |
| Primary school unfinished | 1 (Reference) |  |  |  | 1 (Reference) |  |  |  |
| Primary school finished | 1.11 | 1.08 | 1.14 | <0.001 | 1.03 | 0.99 | 1.07 | 0.114 |
| Secondary school unfinished | 1.14 | 1.13 | 1.16 | <0.001 | 1.06 | 1.04 | 1.09 | <0.001 |
| Secondary school or above | 1.16 | 1.14 | 1.19 | <0.001 | 1.10 | 1.07 | 1.13 | <0.001 |
| Household wealth quintile |  |  |  |  |  |  |  |  |
| Q1 (Poorest) | 1 (Reference) |  |  |  | 1 (Reference) |  |  |  |
| Q2 | 1.20 | 1.16 | 1.24 | <0.001 | 1.05 | 1.02 | 1.09 | 0.001 |
| Q3 | 1.29 | 1.25 | 1.33 | <0.001 | 1.09 | 1.06 | 1.13 | <0.001 |
| Q4 | 1.37 | 1.32 | 1.41 | <0.001 | 1.12 | 1.08 | 1.15 | <0.001 |
| Q5 (Richest) | 1.41 | 1.36 | 1.46 | <0.001 | 1.16 | 1.12 | 1.20 | <0.001 |
| BMI |  |  |  |  |  |  |  |  |
| <18.5 kg/m2 | 1.08 | 1.05 | 1.10 | <0.001 | 1.08 | 1.05 | 1.11 | <0.001 |
| 18.5-22.9 kg/m2 | 1 (Reference) |  |  |  | 1 (Reference) |  |  |  |
| 23.0-24.9 kg/m2 | 0.94 | 0.92 | 0.96 | <0.001 | 0.94 | 0.92 | 0.97 | <0.001 |
| 25.0-27.4 kg/m2 | 0.95 | 0.93 | 0.97 | <0.001 | 0.95 | 0.93 | 0.97 | <0.001 |
| 27.5-29.9 kg/m2 | 0.98 | 0.96 | 1.01 | 0.216 | 0.99 | 0.97 | 1.02 | 0.640 |
| 30.0 kg/m2 | 1.06 | 1.03 | 1.09 | <0.001 | 1.07 | 1.05 | 1.10 | <0.001 |
| Tobacco, smoked | 0.76 | 0.74 | 0.79 | <0.001 | 0.75 | 0.72 | 0.79 | <0.001 |
| Tobacco, smokeless | 0.81 | 0.79 | 0.84 | <0.001 | 0.83 | 0.80 | 0.86 | <0.001 |
| Currently married | 1.10 | 1.08 | 1.13 | <0.001 | 1.10 | 1.07 | 1.12 | <0.001 |
| Female | 1.59 | 1.54 | 1.64 | <0.001 | 1.57 | 1.51 | 1.63 | <0.001 |

Abbreviations: CI = Confidence Interval; Q = Quintile

^13^ These covariate-unadjusted regressions contained one of the listed indicators in the table (age group, wealth quintile, education, BMI, Tobacco smoked, Tobacco smokeless, marital status, sex) and a binary indicator for each district (district-level fixed effects) as explanatory variables. Standard errors were adjusted for clustering at the level of the primary sampling unit. Sampling weights were not used.

Treated

|  | Rural | | | | Urban | | | |
| --- | --- | --- | --- | --- | --- | --- | --- | --- |
|  | Relative Risk | 95% CI (low) | 95% CI (high) | P Value | Relative Risk | 95% CI (low) | 95% CI(high) | P Value |
| Age group, y |  |  |  |  |  |  |  |  |
| 15-19 | 1 (Reference) |  |  |  | 1 (Reference) |  |  |  |
| 20-24 | 0.95 | 0.86 | 1.05 | 0.313 | 1.03 | 0.87 | 1.23 | 0.698 |
| 25-29 | 1.02 | 0.93 | 1.12 | 0.635 | 0.98 | 0.84 | 1.16 | 0.851 |
| 30-34 | 1.06 | 0.97 | 1.16 | 0.176 | 1.20 | 1.03 | 1.40 | 0.023 |
| 35-40 | 1.17 | 1.07 | 1.28 | <0.001 | 1.42 | 1.22 | 1.64 | <0.001 |
| 40-44 | 1.33 | 1.22 | 1.45 | <0.001 | 1.92 | 1.66 | 2.23 | <0.001 |
| 45-49 | 1.54 | 1.42 | 1.68 | <0.001 | 2.46 | 2.12 | 2.84 | <0.001 |
| Education |  |  |  |  |  |  |  |  |
| Primary school unfinished | 1 (Reference) |  |  |  | 1 (Reference) |  |  |  |
| Primary school finished | 1.00 | 0.94 | 1.06 | 0.988 | 1.05 | 0.96 | 1.14 | 0.279 |
| Secondary school unfinished | 0.95 | 0.92 | 0.99 | 0.014 | 0.91 | 0.86 | 0.96 | 0.001 |
| Secondary school or above | 0.86 | 0.81 | 0.91 | <0.001 | 0.86 | 0.81 | 0.92 | <0.001 |
| Household wealth quintile |  |  |  |  |  |  |  |  |
| Q1 (Poorest) | 1 (Reference) |  |  |  | 1 (Reference) |  |  |  |
| Q2 | 1.27 | 1.18 | 1.37 | <0.001 | 1.15 | 1.07 | 1.25 | <0.001 |
| Q3 | 1.36 | 1.27 | 1.46 | <0.001 | 1.26 | 1.17 | 1.36 | <0.001 |
| Q4 | 1.49 | 1.38 | 1.60 | <0.001 | 1.42 | 1.31 | 1.53 | <0.001 |
| Q5 (Richest) | 1.64 | 1.52 | 1.76 | <0.001 | 1.55 | 1.43 | 1.69 | <0.001 |
| BMI |  |  |  |  |  |  |  |  |
| <18.5 kg/m2 | 1.06 | 1.01 | 1.12 | 0.026 | 0.99 | 0.89 | 1.10 | 0.814 |
| 18.5-22.9 kg/m2 | 1 (Reference) |  |  |  | 1 (Reference) |  |  |  |
| 23.0-24.9 kg/m2 | 1.07 | 1.02 | 1.12 | 0.008 | 1.10 | 1.03 | 1.18 | 0.007 |
| 25.0-27.4 kg/m2 | 1.16 | 1.10 | 1.22 | <0.001 | 1.27 | 1.18 | 1.35 | <0.001 |
| 27.5-29.9 kg/m2 | 1.32 | 1.25 | 1.41 | <0.001 | 1.53 | 1.42 | 1.64 | <0.001 |
| 30.0 kg/m2 | 1.70 | 1.61 | 1.79 | <0.001 | 2.02 | 1.90 | 2.15 | <0.001 |
| Tobacco, smoked | 0.69 | 0.64 | 0.75 | <0.001 | 0.73 | 0.65 | 0.81 | <0.001 |
| Tobacco, smokeless | 0.86 | 0.82 | 0.91 | <0.001 | 0.86 | 0.80 | 0.92 | <0.001 |
| Currently married | 1.23 | 1.17 | 1.29 | <0.001 | 1.33 | 1.26 | 1.42 | <0.001 |
| Female | 1.86 | 1.74 | 1.98 | <0.001 | 1.80 | 1.67 | 1.94 | <0.001 |

Abbreviations: CI = Confidence Interval; Q = Quintile

^13^ These covariate-unadjusted regressions contained one of the listed indicators in the table (age group, wealth quintile, education, BMI, Tobacco smoked, Tobacco smokeless, marital status, sex) and a binary indicator for each district (district-level fixed effects) as explanatory variables. Standard errors were adjusted for clustering at the level of the primary sampling unit. Sampling weights were not used.

Controlled

|  | Rural | | | | Urban | | | |
| --- | --- | --- | --- | --- | --- | --- | --- | --- |
|  | Relative Risk | 95% CI (low) | 95% CI (high) | P Value | Relative Risk | 95% CI (low) | 95%CI (high) | P Value |
| Age group, y |  |  |  |  |  |  |  |  |
| 15-19 | 1 (Reference) |  |  |  | 1 (Reference) |  |  |  |
| 20-24 | 0.91 | 0.82 | 1.00 | 0.056 | 1.03 | 0.85 | 1.23 | 0.788 |
| 25-29 | 0.90 | 0.82 | 1.00 | 0.044 | 0.88 | 0.74 | 1.05 | 0.157 |
| 30-34 | 0.84 | 0.76 | 0.92 | <0.001 | 0.9 | 0.76 | 1.07 | 0.217 |
| 35-40 | 0.77 | 0.70 | 0.85 | <0.001 | 0.87 | 0.74 | 1.03 | 0.113 |
| 40-44 | 0.75 | 0.68 | 0.82 | <0.001 | 1.08 | 0.92 | 1.27 | 0.342 |
| 45-49 | 0.77 | 0.70 | 0.85 | <0.001 | 1.33 | 1.13 | 1.56 | <0.001 |
| Education |  |  |  |  |  |  |  |  |
| Primary school unfinished | 1 (Reference) |  |  |  | 1 (Reference) |  |  |  |
| Primary school finished | 1.03 | 0.95 | 1.13 | 0.427 | 1.07 | 0.95 | 1.21 | 0.26 |
| Secondary school unfinished | 1.09 | 1.03 | 1.14 | 0.001 | 0.97 | 0.9 | 1.05 | 0.521 |
| Secondary school or above | 1.07 | 1.00 | 1.14 | 0.053 | 0.97 | 0.89 | 1.05 | 0.46 |
| Household wealth quintile |  |  |  |  |  |  |  |  |
| Q1 (Poorest) | 1 (Reference) |  |  |  | 1 (Reference) |  |  |  |
| Q2 | 1.20 | 1.09 | 1.31 | <0.001 | 1.15 | 1.03 | 1.27 | 0.011 |
| Q3 | 1.26 | 1.15 | 1.38 | <0.001 | 1.16 | 1.04 | 1.29 | 0.006 |
| Q4 | 1.32 | 1.20 | 1.44 | <0.001 | 1.4 | 1.26 | 1.56 | <0.001 |
| Q5 (Richest) | 1.37 | 1.25 | 1.50 | <0.001 | 1.44 | 1.29 | 1.62 | <0.001 |
| BMI |  |  |  |  |  |  |  |  |
| <18.5 kg/m2 | 1.20 | 1.13 | 1.27 | <0.001 | 1.13 | 1 | 1.28 | 0.057 |
| 18.5-22.9 kg/m2 | 1 (Reference) |  |  |  | 1 (Reference) |  |  |  |
| 23.0-24.9 kg/m2 | 0.85 | 0.80 | 0.91 | <0.001 | 0.93 | 0.85 | 1.03 | 0.156 |
| 25.0-27.4 kg/m2 | 0.83 | 0.77 | 0.89 | <0.001 | 0.96 | 0.87 | 1.05 | 0.34 |
| 27.5-29.9 kg/m2 | 0.89 | 0.82 | 0.97 | 0.006 | 1.07 | 0.97 | 1.19 | 0.149 |
| 30.0 kg/m2 | 1.10 | 1.01 | 1.19 | 0.028 | 1.42 | 1.3 | 1.55 | <0.001 |
| Tobacco, smoked | 0.67 | 0.60 | 0.74 | <0.001 | 0.63 | 0.54 | 0.74 | <0.001 |
| Tobacco, smokeless | 0.76 | 0.71 | 0.82 | <0.001 | 0.8 | 0.72 | 0.89 | <0.001 |
| Currently married | 1.03 | 0.97 | 1.09 | 0.399 | 1.14 | 1.06 | 1.24 | 0.001 |
| Female | 2.05 | 1.89 | 2.24 | <0.001 | 2.07 | 1.85 | 2.31 | <0.001 |

Abbreviations: CI = Confidence Interval; Q = Quintile

^13^ These covariate-unadjusted regressions contained one of the listed indicators in the table (age group, wealth quintile, education, BMI, Tobacco smoked, Tobacco smokeless, marital status, sex) and a binary indicator for each district (district-level fixed effects) as explanatory variables. Standard errors were adjusted for clustering at the level of the primary sampling unit. Sampling weights were not used.

# Table AC. Predictors of reaching each cascade step using covariate-unadjusted poisson regressions with sampling weights^14^

Screened

|  | Rural | | | | Urban | | | |
| --- | --- | --- | --- | --- | --- | --- | --- | --- |
|  | Relative Risk | 95% CI (low) | 95% CI (high) | P Value | Relative Risk | 95% CI (low) | 95% CI (high) | P Value |
| Age group, y |  |  |  |  |  |  |  |  |
| 15-19 | 1 (Reference) |  |  |  | 1 (Reference) |  |  |  |
| 20-24 | 1.16 | 1.12 | 1.20 | <0.001 | 1.21 | 1.16 | 1.27 | <0.001 |
| 25-29 | 1.25 | 1.21 | 1.29 | <0.001 | 1.25 | 1.20 | 1.31 | <0.001 |
| 30-34 | 1.22 | 1.18 | 1.27 | <0.001 | 1.22 | 1.17 | 1.28 | <0.001 |
| 35-40 | 1.21 | 1.17 | 1.25 | <0.001 | 1.28 | 1.23 | 1.34 | <0.001 |
| 40-44 | 1.21 | 1.17 | 1.26 | <0.001 | 1.32 | 1.27 | 1.38 | <0.001 |
| 45-49 | 1.23 | 1.19 | 1.27 | <0.001 | 1.33 | 1.28 | 1.39 | <0.001 |
| Education |  |  |  |  |  |  |  |  |
| Primary school unfinished | 1 (Reference) |  |  |  | 1 (Reference) |  |  |  |
| Primary school finished | 1.02 | 1.00 | 1.05 | 0.103 | 1.01 | 0.98 | 1.05 | 0.471 |
| Secondary school unfinished | 1.04 | 1.02 | 1.05 | <0.001 | 1.01 | 0.99 | 1.03 | 0.199 |
| Secondary school or above | 1.05 | 1.03 | 1.07 | <0.001 | 1.08 | 1.06 | 1.10 | <0.001 |
| Household wealth quintile |  |  |  |  |  |  |  |  |
| Q1 (Poorest) | 1 (Reference) |  |  |  | 1 (Reference) |  |  |  |
| Q2 | 1.15 | 1.12 | 1.19 | <0.001 | 1.08 | 1.05 | 1.10 | <0.001 |
| Q3 | 1.22 | 1.19 | 1.26 | <0.001 | 1.07 | 1.04 | 1.10 | <0.001 |
| Q4 | 1.27 | 1.24 | 1.30 | <0.001 | 1.14 | 1.11 | 1.17 | <0.001 |
| Q5 (Richest) | 1.33 | 1.30 | 1.37 | <0.001 | 1.21 | 1.18 | 1.24 | <0.001 |
| BMI |  |  |  |  |  |  |  |  |
| <18.5 kg/m2 | 0.99 | 0.97 | 1.01 | 0.441 | 0.99 | 0.95 | 1.02 | 0.443 |
| 18.5-22.9 kg/m2 | 1 (Reference) |  |  |  | 1 (Reference) |  |  |  |
| 23.0-24.9 kg/m2 | 1.03 | 1.01 | 1.05 | 0.001 | 1.06 | 1.04 | 1.08 | <0.001 |
| 25.0-27.4 kg/m2 | 1.06 | 1.04 | 1.08 | <0.001 | 1.03 | 1.01 | 1.05 | 0.008 |
| 27.5-29.9 kg/m2 | 1.07 | 1.05 | 1.10 | <0.001 | 1.08 | 1.06 | 1.11 | <0.001 |
| 30.0 kg/m2 | 1.12 | 1.09 | 1.15 | <0.001 | 1.17 | 1.14 | 1.19 | <0.001 |
| Tobacco, smoked | 0.86 | 0.85 | 0.88 | <0.001 | 0.92 | 0.90 | 0.94 | <0.001 |
| Tobacco, smokeless | 0.89 | 0.87 | 0.90 | <0.001 | 0.84 | 0.82 | 0.86 | <0.001 |
| Currently married | 1.16 | 1.14 | 1.18 | <0.001 | 1.16 | 1.14 | 1.19 | <0.001 |
| Female | 1.25 | 1.23 | 1.26 | <0.001 | 1.20 | 1.18 | 1.21 | <0.001 |

Abbreviations: CI = Confidence Interval; Q = Quintile

^14^ These covariate-unadjusted regressions contained one of the listed indicators in the table (age group, wealth quintile, education, BMI, Tobacco smoked, Tobacco smokeless, marital status, sex) and a binary indicator for each district (district-level fixed effects) as explanatory variables. Standard errors were adjusted for clustering at the level of the primary sampling unit. Sampling weights were used to adjust for the survey design and the differing number of women and men in the survey.

Aware

|  | Rural | | | | Urban | | | |
| --- | --- | --- | --- | --- | --- | --- | --- | --- |
|  | Relative Risk | 95% CI (low) | 95% CI (high) | P Value | Relative Risk | 95% CI (low) | 95% CI (high) | P Value |
| Age group, y |  |  |  |  |  |  |  |  |
| 15-19 | 1 (Reference) |  |  |  | 1 (Reference) |  |  |  |
| 20-24 | 1.04 | 1.00 | 1.08 | 0.082 | 1.07 | 1.02 | 1.13 | 0.011 |
| 25-29 | 1.01 | 0.97 | 1.05 | 0.664 | 0.98 | 0.93 | 1.03 | 0.491 |
| 30-34 | 0.92 | 0.89 | 0.96 | <0.001 | 0.95 | 0.90 | 1.00 | 0.038 |
| 35-40 | 0.86 | 0.83 | 0.90 | <0.001 | 0.94 | 0.90 | 0.99 | 0.018 |
| 40-44 | 0.88 | 0.85 | 0.92 | <0.001 | 0.98 | 0.93 | 1.02 | 0.324 |
| 45-49 | 0.91 | 0.88 | 0.95 | <0.001 | 1.05 | 1.00 | 1.10 | 0.042 |
| Education |  |  |  |  |  |  |  |  |
| Primary school unfinished | 1 (Reference) |  |  |  | 1 (Reference) |  |  |  |
| Primary school finished | 1.02 | 0.99 | 1.06 | 0.172 | 0.96 | 0.91 | 1.00 | 0.050 |
| Secondary school unfinished | 1.06 | 1.04 | 1.08 | <0.001 | 1.03 | 1.00 | 1.05 | 0.074 |
| Secondary school or above | 1.03 | 1.01 | 1.06 | 0.015 | 1.08 | 1.05 | 1.11 | <0.001 |
| Household wealth quintile |  |  |  |  |  |  |  |  |
| Q1 (Poorest) | 1 (Reference) |  |  |  | 1 (Reference) |  |  |  |
| Q2 | 1.21 | 1.16 | 1.25 | <0.001 | 1.07 | 1.03 | 1.10 | <0.001 |
| Q3 | 1.28 | 1.24 | 1.33 | <0.001 | 1.09 | 1.06 | 1.13 | <0.001 |
| Q4 | 1.29 | 1.25 | 1.34 | <0.001 | 1.17 | 1.13 | 1.21 | <0.001 |
| Q5 (Richest) | 1.33 | 1.28 | 1.38 | <0.001 | 1.23 | 1.19 | 1.27 | <0.001 |
| BMI |  |  |  |  |  |  |  |  |
| <18.5 kg/m2 | 1.13 | 1.10 | 1.16 | <0.001 | 1.12 | 1.07 | 1.16 | <0.001 |
| 18.5-22.9 kg/m2 | 1 (Reference) |  |  |  | 1 (Reference) |  |  |  |
| 23.0-24.9 kg/m2 | 0.90 | 0.88 | 0.93 | <0.001 | 0.92 | 0.89 | 0.95 | <0.001 |
| 25.0-27.4 kg/m2 | 0.92 | 0.90 | 0.95 | <0.001 | 0.94 | 0.91 | 0.96 | <0.001 |
| 27.5-29.9 kg/m2 | 0.92 | 0.89 | 0.95 | <0.001 | 1.08 | 1.05 | 1.11 | <0.001 |
| 30.0 kg/m2 | 1.04 | 1.00 | 1.07 | 0.049 | 1.14 | 1.11 | 1.18 | <0.001 |
| Tobacco, smoked | 0.79 | 0.78 | 0.81 | <0.001 | 0.83 | 0.80 | 0.85 | <0.001 |
| Tobacco, smokeless | 0.76 | 0.74 | 0.77 | <0.001 | 0.66 | 0.64 | 0.68 | <0.001 |
| Currently married | 1.06 | 1.04 | 1.08 | <0.001 | 1.11 | 1.08 | 1.13 | <0.001 |
| Female | 1.55 | 1.53 | 1.58 | <0.001 | 1.53 | 1.50 | 1.56 | <0.001 |

Abbreviations: CI = Confidence Interval; Q = Quintile

^14^ These covariate-unadjusted regressions contained one of the listed indicators in the table (age group, wealth quintile, education, BMI, Tobacco smoked, Tobacco smokeless, marital status, sex) and a binary indicator for each district (district-level fixed effects) as explanatory variables. Standard errors were adjusted for clustering at the level of the primary sampling unit. Sampling weights were used to adjust for the survey design and the differing number of women and men in the survey.

Treated

|  | Rural | | | | Urban | | | |
| --- | --- | --- | --- | --- | --- | --- | --- | --- |
|  | Relative Risk | 95% CI (low) | 95% CI (high) | P Value | Relative Risk | 95% CI (low) | 95% CI (high) | P Value |
| Age group, y |  |  |  |  |  |  |  |  |
| 15-19 | 1 (Reference) |  |  |  | 1 (Reference) |  |  |  |
| 20-24 | 0.71 | 0.65 | 0.78 | <0.001 | 1.28 | 1.12 | 1.47 | <0.001 |
| 25-29 | 0.79 | 0.73 | 0.86 | <0.001 | 1.07 | 0.94 | 1.22 | 0.324 |
| 30-34 | 0.83 | 0.77 | 0.90 | <0.001 | 1.33 | 1.17 | 1.52 | <0.001 |
| 35-40 | 0.86 | 0.80 | 0.93 | <0.001 | 1.63 | 1.44 | 1.85 | <0.001 |
| 40-44 | 1.10 | 1.03 | 1.19 | 0.008 | 2.55 | 2.26 | 2.87 | <0.001 |
| 45-49 | 1.28 | 1.19 | 1.38 | <0.001 | 3.16 | 2.80 | 3.56 | <0.001 |
| Education |  |  |  |  |  |  |  |  |
| Primary school unfinished | 1 (Reference) |  |  |  | 1 (Reference) |  |  |  |
| Primary school finished | 0.94 | 0.89 | 1.00 | 0.067 | 0.99 | 0.92 | 1.07 | 0.831 |
| Secondary school unfinished | 0.87 | 0.84 | 0.91 | <0.001 | 0.92 | 0.88 | 0.97 | 0.001 |
| Secondary school or above | 0.85 | 0.81 | 0.89 | <0.001 | 0.91 | 0.87 | 0.96 | <0.001 |
| Household wealth quintile |  |  |  |  |  |  |  |  |
| Q1 (Poorest) | 1 (Reference) |  |  |  | 1 (Reference) |  |  |  |
| Q2 | 1.11 | 1.04 | 1.19 | 0.001 | 1.07 | 1.00 | 1.14 | 0.047 |
| Q3 | 1.26 | 1.18 | 1.34 | <0.001 | 1.34 | 1.26 | 1.42 | <0.001 |
| Q4 | 1.30 | 1.22 | 1.38 | <0.001 | 1.55 | 1.46 | 1.65 | <0.001 |
| Q5 (Richest) | 1.52 | 1.43 | 1.62 | <0.001 | 1.75 | 1.65 | 1.87 | <0.001 |
| BMI |  |  |  |  |  |  |  |  |
| <18.5 kg/m2 | 1.13 | 1.07 | 1.18 | <0.001 | 0.92 | 0.83 | 1.01 | 0.067 |
| 18.5-22.9 kg/m2 | 1 (Reference) |  |  |  | 1 (Reference) |  |  |  |
| 23.0-24.9 kg/m2 | 1.01 | 0.96 | 1.06 | 0.658 | 1.04 | 0.99 | 1.11 | 0.145 |
| 25.0-27.4 kg/m2 | 1.20 | 1.14 | 1.25 | <0.001 | 1.32 | 1.25 | 1.40 | <0.001 |
| 27.5-29.9 kg/m2 | 1.28 | 1.21 | 1.35 | <0.001 | 1.96 | 1.86 | 2.07 | <0.001 |
| 30.0 kg/m2 | 1.81 | 1.71 | 1.92 | <0.001 | 2.42 | 2.29 | 2.55 | <0.001 |
| Tobacco, smoked | 0.67 | 0.64 | 0.70 | <0.001 | 0.76 | 0.72 | 0.80 | <0.001 |
| Tobacco, smokeless | 0.70 | 0.67 | 0.73 | <0.001 | 0.64 | 0.61 | 0.68 | <0.001 |
| Currently married | 1.20 | 1.15 | 1.25 | <0.001 | 1.77 | 1.68 | 1.86 | <0.001 |
| Female | 1.81 | 1.76 | 1.87 | <0.001 | 1.71 | 1.65 | 1.77 | <0.001 |

Abbreviations: CI = Confidence Interval; Q = Quintile

^14^ These covariate-unadjusted regressions contained one of the listed indicators in the table (age group, wealth quintile, education, BMI, Tobacco smoked, Tobacco smokeless, marital status, sex) and a binary indicator for each district (district-level fixed effects) as explanatory variables. Standard errors were adjusted for clustering at the level of the primary sampling unit. Sampling weights were used to adjust for the survey design and the differing number of women and men in the survey.

Controlled

|  | Rural | | | | Urban | | | |
| --- | --- | --- | --- | --- | --- | --- | --- | --- |
|  | Relative Risk | 95% CI (low) | 95% CI (high) | P Value | Relative Risk | 95% CI (low) | 95% CI (high) | P Value |
| Age group, y |  |  |  |  |  |  |  |  |
| 15-19 | 1 (Reference) |  |  |  | 1 (Reference) |  |  |  |
| 20-24 | 0.69 | 0.63 | 0.75 | <0.001 | 1.26 | 1.09 | 1.45 | 0.002 |
| 25-29 | 0.69 | 0.63 | 0.75 | <0.001 | 0.99 | 0.86 | 1.14 | 0.914 |
| 30-34 | 0.64 | 0.59 | 0.70 | <0.001 | 0.96 | 0.84 | 1.11 | 0.608 |
| 35-40 | 0.56 | 0.52 | 0.61 | <0.001 | 0.95 | 0.83 | 1.09 | 0.491 |
| 40-44 | 0.57 | 0.53 | 0.62 | <0.001 | 1.49 | 1.31 | 1.70 | <0.001 |
| 45-49 | 0.69 | 0.64 | 0.74 | <0.001 | 1.68 | 1.48 | 1.91 | <0.001 |
| Education |  |  |  |  |  |  |  |  |
| Primary school unfinished | 1 (Reference) |  |  |  | 1 (Reference) |  |  |  |
| Primary school finished | 0.91 | 0.84 | 0.99 | 0.034 | 1.02 | 0.92 | 1.14 | 0.648 |
| Secondary school unfinished | 0.94 | 0.90 | 0.99 | 0.013 | 0.91 | 0.86 | 0.97 | 0.004 |
| Secondary school or above | 0.94 | 0.89 | 1.00 | 0.050 | 0.94 | 0.88 | 1.00 | 0.051 |
| Household wealth quintile |  |  |  |  |  |  |  |  |
| Q1 (Poorest) | 1 (Reference) |  |  |  | 1 (Reference) |  |  |  |
| Q2 | 1.07 | 0.99 | 1.15 | 0.093 | 1.10 | 1.01 | 1.20 | 0.024 |
| Q3 | 1.12 | 1.04 | 1.21 | 0.003 | 1.04 | 0.95 | 1.13 | 0.381 |
| Q4 | 1.08 | 1.00 | 1.16 | 0.054 | 1.56 | 1.43 | 1.69 | <0.001 |
| Q5 (Richest) | 1.21 | 1.12 | 1.30 | <0.001 | 1.61 | 1.49 | 1.75 | <0.001 |
| BMI |  |  |  |  |  |  |  |  |
| <18.5 kg/m2 | 1.29 | 1.22 | 1.37 | <0.001 | 1.10 | 0.99 | 1.22 | 0.065 |
| 18.5-22.9 kg/m2 | 1 (Reference) |  |  |  | 1 (Reference) |  |  |  |
| 23.0-24.9 kg/m2 | 0.85 | 0.80 | 0.90 | <0.001 | 0.84 | 0.78 | 0.91 | <0.001 |
| 25.0-27.4 kg/m2 | 0.80 | 0.76 | 0.86 | <0.001 | 0.95 | 0.89 | 1.03 | 0.201 |
| 27.5-29.9 kg/m2 | 0.81 | 0.74 | 0.87 | <0.001 | 1.43 | 1.33 | 1.54 | <0.001 |
| 30.0 kg/m2 | 1.13 | 1.05 | 1.23 | 0.002 | 1.67 | 1.56 | 1.79 | <0.001 |
| Tobacco, smoked | 0.56 | 0.52 | 0.60 | <0.001 | 0.63 | 0.58 | 0.68 | <0.001 |
| Tobacco, smokeless | 0.59 | 0.56 | 0.63 | <0.001 | 0.58 | 0.54 | 0.63 | <0.001 |
| Currently married | 0.93 | 0.88 | 0.97 | 0.003 | 1.34 | 1.26 | 1.43 | <0.001 |
| Female | 2.05 | 1.96 | 2.13 | <0.001 | 2.08 | 1.99 | 2.18 | <0.001 |

Abbreviations: CI = Confidence Interval; Q = Quintile

^14^ These covariate-unadjusted regressions contained one of the listed indicators in the table (age group, wealth quintile, education, BMI, Tobacco smoked, Tobacco smokeless, marital status, sex) and a binary indicator for each district (district-level fixed effects) as explanatory variables. Standard errors were adjusted for clustering at the level of the primary sampling unit. Sampling weights were used to adjust for the survey design and the differing number of women and men in the survey.

# Table AD. Predictors of reaching each cascade step using covariate-unadjusted poisson regressions among women only^15^

Screened

|  | Rural | | | | Urban | | | |
| --- | --- | --- | --- | --- | --- | --- | --- | --- |
|  | Relative Risk | 95% CI (low) | 95% CI (high) | P Value | Relative Risk | 95% CI (low) | 95% CI (high) | P Value |
| Age group, y |  |  |  |  |  |  |  |  |
| 15-19 | 1 (Reference) |  |  |  | 1 (Reference) |  |  |  |
| 20-24 | 1.24 | 1.20 | 1.27 | <0.001 | 1.17 | 1.13 | 1.21 | <0.001 |
| 25-29 | 1.29 | 1.26 | 1.32 | <0.001 | 1.21 | 1.17 | 1.25 | <0.001 |
| 30-34 | 1.26 | 1.23 | 1.29 | <0.001 | 1.22 | 1.18 | 1.26 | <0.001 |
| 35-40 | 1.22 | 1.19 | 1.25 | <0.001 | 1.21 | 1.17 | 1.25 | <0.001 |
| 40-44 | 1.21 | 1.18 | 1.24 | <0.001 | 1.21 | 1.17 | 1.25 | <0.001 |
| 45-49 | 1.19 | 1.16 | 1.22 | <0.001 | 1.21 | 1.17 | 1.25 | <0.001 |
| Education |  |  |  |  |  |  |  |  |
| Primary school unfinished | 1 (Reference) |  |  |  | 1 (Reference) |  |  |  |
| Primary school finished | 1.08 | 1.06 | 1.09 | <0.001 | 1.04 | 1.02 | 1.06 | <0.001 |
| Secondary school unfinished | 1.10 | 1.09 | 1.11 | <0.001 | 1.05 | 1.04 | 1.06 | <0.001 |
| Secondary school or above | 1.12 | 1.11 | 1.14 | <0.001 | 1.07 | 1.06 | 1.08 | <0.001 |
| Household wealth quintile |  |  |  |  |  |  |  |  |
| Q1 (Poorest) | 1 (Reference) |  |  |  | 1 (Reference) |  |  |  |
| Q2 | 1.14 | 1.12 | 1.16 | <0.001 | 1.05 | 1.03 | 1.06 | <0.001 |
| Q3 | 1.21 | 1.19 | 1.23 | <0.001 | 1.07 | 1.05 | 1.08 | <0.001 |
| Q4 | 1.26 | 1.24 | 1.29 | <0.001 | 1.08 | 1.07 | 1.10 | <0.001 |
| Q5 (Richest) | 1.31 | 1.29 | 1.34 | <0.001 | 1.11 | 1.09 | 1.12 | <0.001 |
| BMI |  |  |  |  |  |  |  |  |
| <18.5 kg/m2 | 0.99 | 0.98 | 1.00 | 0.104 | 0.97 | 0.95 | 0.99 | <0.001 |
| 18.5-22.9 kg/m2 | 1 (Reference) |  |  |  | 1 (Reference) |  |  |  |
| 23.0-24.9 kg/m2 | 1.03 | 1.02 | 1.04 | <0.001 | 1.01 | 1.00 | 1.03 | 0.010 |
| 25.0-27.4 kg/m2 | 1.05 | 1.04 | 1.06 | <0.001 | 1.02 | 1.01 | 1.03 | <0.001 |
| 27.5-29.9 kg/m2 | 1.07 | 1.06 | 1.09 | <0.001 | 1.02 | 1.01 | 1.04 | <0.001 |
| 30.0 kg/m2 | 1.09 | 1.08 | 1.11 | <0.001 | 1.05 | 1.04 | 1.06 | <0.001 |
| Tobacco, smoked | 0.96 | 0.93 | 0.98 | 0.001 | 0.97 | 0.95 | 1.00 | 0.055 |
| Tobacco, smokeless | 0.96 | 0.95 | 0.98 | <0.001 | 0.98 | 0.97 | 1.00 | 0.033 |
| Currently married | 1.15 | 1.14 | 1.17 | <0.001 | 1.11 | 1.10 | 1.12 | <0.001 |

Abbreviations: CI = Confidence Interval; Q = Quintile

^15^ These covariate-unadjusted regressions contained one of the listed indicators in the table (age group, wealth quintile, education, BMI, Tobacco smoked, Tobacco smokeless, marital status) and a binary indicator for each district (district-level fixed effects) as explanatory variables. Standard errors were adjusted for clustering at the level of the primary sampling unit. Sampling weights were not used.

Aware

|  | Rural | | | | Urban | | | |
| --- | --- | --- | --- | --- | --- | --- | --- | --- |
|  | Relative Risk | 95% CI (low) | 95% CI (high) | P Value | Relative Risk | 95% CI (low) | 95% CI (high) | P Value |
| Age group, y |  |  |  |  |  |  |  |  |
| 15-19 | 1 (Reference) |  |  |  | 1 (Reference) |  |  |  |
| 20-24 | 1.15 | 1.11 | 1.19 | <0.001 | 1.11 | 1.06 | 1.16 | <0.001 |
| 25-29 | 1.12 | 1.08 | 1.15 | <0.001 | 1.06 | 1.01 | 1.10 | 0.010 |
| 30-34 | 1.03 | 0.99 | 1.06 | 0.120 | 1.00 | 0.96 | 1.05 | 0.908 |
| 35-40 | 0.94 | 0.91 | 0.97 | <0.001 | 0.95 | 0.91 | 0.99 | 0.010 |
| 40-44 | 0.92 | 0.89 | 0.95 | <0.001 | 0.94 | 0.90 | 0.98 | 0.007 |
| 45-49 | 0.93 | 0.90 | 0.96 | <0.001 | 1.00 | 0.95 | 1.04 | 0.846 |
| Education |  |  |  |  |  |  |  |  |
| Primary school unfinished | 1 (Reference) |  |  |  | 1 (Reference) |  |  |  |
| Primary school finished | 1.14 | 1.11 | 1.17 | <0.001 | 1.06 | 1.02 | 1.10 | 0.004 |
| Secondary school unfinished | 1.21 | 1.19 | 1.23 | <0.001 | 1.11 | 1.08 | 1.13 | <0.001 |
| Secondary school or above | 1.28 | 1.25 | 1.31 | <0.001 | 1.15 | 1.12 | 1.18 | <0.001 |
| Household wealth quintile |  |  |  |  |  |  |  |  |
| Q1 (Poorest) | 1 (Reference) |  |  |  | 1 (Reference) |  |  |  |
| Q2 | 1.21 | 1.17 | 1.25 | <0.001 | 1.04 | 1.01 | 1.07 | 0.007 |
| Q3 | 1.30 | 1.26 | 1.34 | <0.001 | 1.08 | 1.04 | 1.11 | <0.001 |
| Q4 | 1.38 | 1.33 | 1.43 | <0.001 | 1.10 | 1.06 | 1.13 | <0.001 |
| Q5 (Richest) | 1.43 | 1.38 | 1.48 | <0.001 | 1.14 | 1.10 | 1.18 | <0.001 |
| BMI |  |  |  |  |  |  |  |  |
| <18.5 kg/m2 | 1.05 | 1.03 | 1.07 | <0.001 | 1.06 | 1.03 | 1.10 | <0.001 |
| 18.5-22.9 kg/m2 | 1 (Reference) |  |  |  | 1 (Reference) |  |  |  |
| 23.0-24.9 kg/m2 | 0.95 | 0.93 | 0.97 | <0.001 | 0.95 | 0.93 | 0.98 | <0.001 |
| 25.0-27.4 kg/m2 | 0.95 | 0.93 | 0.97 | <0.001 | 0.94 | 0.92 | 0.97 | <0.001 |
| 27.5-29.9 kg/m2 | 0.98 | 0.95 | 1.00 | 0.102 | 0.96 | 0.94 | 0.99 | 0.009 |
| 30.0 kg/m2 | 1.02 | 1.00 | 1.05 | 0.078 | 1.02 | 0.99 | 1.05 | 0.135 |
| Tobacco, smoked | 0.94 | 0.89 | 0.98 | 0.009 | 0.99 | 0.92 | 1.06 | 0.695 |
| Tobacco, smokeless | 0.89 | 0.87 | 0.92 | <0.001 | 0.98 | 0.94 | 1.02 | 0.267 |
| Currently married | 1.08 | 1.06 | 1.10 | <0.001 | 1.07 | 1.05 | 1.10 | <0.001 |

Abbreviations: CI = Confidence Interval; Q = Quintile

^15^These covariate-unadjusted regressions contained one of the listed indicators in the table (age group, wealth quintile, education, BMI, Tobacco smoked, Tobacco smokeless, marital status) and a binary indicator for each district (district-level fixed effects) as explanatory variables. Standard errors were adjusted for clustering at the level of the primary sampling unit. Sampling weights were not used.

Treated

|  | Rural | | | | Urban | | | |
| --- | --- | --- | --- | --- | --- | --- | --- | --- |
|  | Relative Risk | 95% CI (low) | 95% CI (high) | P Value | Relative Risk | 95% CI (low) | 95% CI (high) | P Value |
| Age group, y |  |  |  |  |  |  |  |  |
| 15-19 | 1 (Reference) |  |  |  | 1 (Reference) |  |  |  |
| 20-24 | 0.98 | 0.89 | 1.08 | 0.699 | 1.02 | 0.85 | 1.22 | 0.864 |
| 25-29 | 1.04 | 0.94 | 1.14 | 0.435 | 0.97 | 0.81 | 1.15 | 0.694 |
| 30-34 | 1.09 | 0.99 | 1.19 | 0.090 | 1.18 | 1.00 | 1.38 | 0.050 |
| 35-40 | 1.19 | 1.08 | 1.30 | <0.001 | 1.39 | 1.19 | 1.63 | <0.001 |
| 40-44 | 1.33 | 1.22 | 1.46 | <0.001 | 1.83 | 1.57 | 2.14 | <0.001 |
| 45-49 | 1.54 | 1.41 | 1.69 | <0.001 | 2.34 | 2.01 | 2.73 | <0.001 |
| Education |  |  |  |  |  |  |  |  |
| Primary school unfinished | 1 (Reference) |  |  |  | 1 (Reference) |  |  |  |
| Primary school finished | 1.03 | 0.97 | 1.10 | 0.319 | 1.08 | 0.99 | 1.18 | 0.097 |
| Secondary school unfinished | 1.02 | 0.98 | 1.06 | 0.443 | 0.95 | 0.90 | 1.01 | 0.109 |
| Secondary school or above | 0.92 | 0.87 | 0.97 | 0.005 | 0.88 | 0.83 | 0.94 | <0.001 |
| Household wealth quintile |  |  |  |  |  |  |  |  |
| Q1 (Poorest) | 1 (Reference) |  |  |  | 1 (Reference) |  |  |  |
| Q2 | 1.30 | 1.20 | 1.40 | <0.001 | 1.12 | 1.03 | 1.21 | 0.008 |
| Q3 | 1.38 | 1.28 | 1.49 | <0.001 | 1.23 | 1.13 | 1.33 | <0.001 |
| Q4 | 1.52 | 1.41 | 1.63 | <0.001 | 1.38 | 1.27 | 1.49 | <0.001 |
| Q5 (Richest) | 1.66 | 1.54 | 1.80 | <0.001 | 1.49 | 1.37 | 1.62 | <0.001 |
| BMI |  |  |  |  |  |  |  |  |
| <18.5 kg/m2 | 1.03 | 0.98 | 1.09 | 0.276 | 0.96 | 0.86 | 1.08 | 0.490 |
| 18.5-22.9 kg/m2 | 1 (Reference) |  |  |  | 1 (Reference) |  |  |  |
| 23.0-24.9 kg/m2 | 1.07 | 1.02 | 1.13 | 0.006 | 1.10 | 1.02 | 1.18 | 0.017 |
| 25.0-27.4 kg/m2 | 1.16 | 1.10 | 1.22 | <0.001 | 1.25 | 1.16 | 1.34 | <0.001 |
| 27.5-29.9 kg/m2 | 1.32 | 1.24 | 1.40 | <0.001 | 1.45 | 1.34 | 1.56 | <0.001 |
| 30.0 kg/m2 | 1.63 | 1.54 | 1.73 | <0.001 | 1.89 | 1.77 | 2.02 | <0.001 |
| Tobacco, smoked | 0.96 | 0.86 | 1.07 | 0.464 | 1.11 | 0.94 | 1.31 | 0.204 |
| Tobacco, smokeless | 1.02 | 0.96 | 1.08 | 0.557 | 1.09 | 1.00 | 1.19 | 0.052 |
| Currently married | 1.17 | 1.11 | 1.23 | <0.001 | 1.25 | 1.17 | 1.33 | <0.001 |

Abbreviations: CI = Confidence Interval; Q = Quintile

^15^ These covariate-unadjusted regressions contained one of the listed indicators in the table (age group, wealth quintile, education, BMI, Tobacco smoked, Tobacco smokeless, marital status) and a binary indicator for each district (district-level fixed effects) as explanatory variables. Standard errors were adjusted for clustering at the level of the primary sampling unit. Sampling weights were not used.

Controlled

|  | Rural | | | | Urban | | | |
| --- | --- | --- | --- | --- | --- | --- | --- | --- |
|  | Relative Risk | 95% CI (low) | 95% CI (high) | P Value | Relative Risk | 95% CI (low) | 95% CI (high) | P Value |
| Age group, y |  |  |  |  |  |  |  |  |
| 15-19 | 1 (Reference) |  |  |  | 1 (Reference) |  |  |  |
| 20-24 | 0.94 | 0.85 | 1.05 | 0.282 | 1.00 | 0.83 | 1.22 | 0.978 |
| 25-29 | 0.93 | 0.84 | 1.03 | 0.193 | 0.85 | 0.70 | 1.02 | 0.079 |
| 30-34 | 0.87 | 0.79 | 0.97 | 0.008 | 0.88 | 0.74 | 1.05 | 0.166 |
| 35-40 | 0.79 | 0.72 | 0.87 | <0.001 | 0.86 | 0.72 | 1.03 | 0.101 |
| 40-44 | 0.76 | 0.69 | 0.84 | <0.001 | 1.02 | 0.86 | 1.21 | 0.798 |
| 45-49 | 0.78 | 0.70 | 0.86 | <0.001 | 1.26 | 1.06 | 1.49 | 0.008 |
| Education |  |  |  |  |  |  |  |  |
| Primary school unfinished | 1 (Reference) |  |  |  | 1 (Reference) |  |  |  |
| Primary school finished | 1.08 | 0.99 | 1.18 | 0.080 | 1.11 | 0.98 | 1.26 | 0.098 |
| Secondary school unfinished | 1.17 | 1.11 | 1.23 | <0.001 | 1.04 | 0.96 | 1.12 | 0.388 |
| Secondary school or above | 1.20 | 1.12 | 1.29 | <0.001 | 1.02 | 0.93 | 1.11 | 0.711 |
| Household wealth quintile |  |  |  |  |  |  |  |  |
| Q1 (Poorest) | 1 (Reference) |  |  |  | 1 (Reference) |  |  |  |
| Q2 | 1.21 | 1.11 | 1.33 | <0.001 | 1.11 | 0.99 | 1.24 | 0.063 |
| Q3 | 1.28 | 1.17 | 1.40 | <0.001 | 1.14 | 1.02 | 1.27 | 0.020 |
| Q4 | 1.34 | 1.22 | 1.47 | <0.001 | 1.36 | 1.22 | 1.52 | <0.001 |
| Q5 (Richest) | 1.38 | 1.26 | 1.52 | <0.001 | 1.40 | 1.25 | 1.57 | <0.001 |
| BMI |  |  |  |  |  |  |  |  |
| <18.5 kg/m2 | 1.16 | 1.09 | 1.23 | <0.001 | 1.08 | 0.95 | 1.23 | 0.261 |
| 18.5-22.9 kg/m2 | 1 (Reference) |  |  |  | 1 (Reference) |  |  |  |
| 23.0-24.9 kg/m2 | 0.86 | 0.80 | 0.92 | <0.001 | 0.93 | 0.84 | 1.03 | 0.145 |
| 25.0-27.4 kg/m2 | 0.83 | 0.78 | 0.90 | <0.001 | 0.95 | 0.86 | 1.04 | 0.281 |
| 27.5-29.9 kg/m2 | 0.90 | 0.82 | 0.98 | 0.014 | 1.01 | 0.91 | 1.12 | 0.897 |
| 30.0 kg/m2 | 1.04 | 0.96 | 1.14 | 0.330 | 1.31 | 1.20 | 1.44 | <0.001 |
| Tobacco, smoked | 0.98 | 0.85 | 1.12 | 0.738 | 0.98 | 0.77 | 1.24 | 0.848 |
| Tobacco, smokeless | 0.90 | 0.83 | 0.98 | 0.014 | 1.05 | 0.93 | 1.19 | 0.408 |
| Currently married | 1.00 | 0.94 | 1.07 | 0.932 | 1.10 | 1.01 | 1.19 | 0.032 |

Abbreviations: CI = Confidence Interval; Q = Quintile

^15^ These covariate-unadjusted regressions contained one of the listed indicators in the table (age group, wealth quintile, education, BMI, Tobacco smoked, Tobacco smokeless, marital status) and a binary indicator for each district (district-level fixed effects) as explanatory variables. Standard errors were adjusted for clustering at the level of the primary sampling unit. Sampling weights were not used.

# Table AE. Predictors of reaching each cascade step using covariate-unadjusted poisson regressions among men only^16^

Screened

|  | Rural | | | | Urban | | | |
| --- | --- | --- | --- | --- | --- | --- | --- | --- |
|  | Relative Risk | 95% CI (low) | 95% CI (high) | P Value | Relative Risk | 95% CI (low) | 95% CI (high) | P Value |
| Age group, y |  |  |  |  |  |  |  |  |
| 15-19 | 1 (Reference) |  |  |  | 1 (Reference) |  |  |  |
| 20-24 | 1.16 | 1.06 | 1.27 | 0.001 | 1.16 | 1.05 | 1.29 | 0.003 |
| 25-29 | 1.35 | 1.24 | 1.46 | <0.001 | 1.22 | 1.11 | 1.35 | <0.001 |
| 30-34 | 1.40 | 1.29 | 1.52 | <0.001 | 1.28 | 1.16 | 1.41 | <0.001 |
| 35-40 | 1.39 | 1.28 | 1.51 | <0.001 | 1.34 | 1.22 | 1.48 | <0.001 |
| 40-44 | 1.45 | 1.33 | 1.57 | <0.001 | 1.39 | 1.27 | 1.53 | <0.001 |
| 45-49 | 1.46 | 1.35 | 1.58 | <0.001 | 1.44 | 1.31 | 1.58 | <0.001 |
| Education |  |  |  |  |  |  |  |  |
| Primary school unfinished | 1 (Reference) |  |  |  | 1 (Reference) |  |  |  |
| Primary school finished | 1.05 | 0.99 | 1.12 | 0.089 | 1.06 | 0.98 | 1.14 | 0.170 |
| Secondary school unfinished | 1.10 | 1.06 | 1.15 | <0.001 | 1.06 | 1.01 | 1.12 | 0.020 |
| Secondary school or above | 1.17 | 1.12 | 1.21 | <0.001 | 1.22 | 1.15 | 1.28 | <0.001 |
| Household wealth quintile |  |  |  |  |  |  |  |  |
| Q1 (Poorest) | 1 (Reference) |  |  |  | 1 (Reference) |  |  |  |
| Q2 | 1.14 | 1.07 | 1.23 | <0.001 | 1.14 | 1.07 | 1.20 | <0.001 |
| Q3 | 1.24 | 1.16 | 1.33 | <0.001 | 1.16 | 1.10 | 1.23 | <0.001 |
| Q4 | 1.32 | 1.23 | 1.41 | <0.001 | 1.24 | 1.17 | 1.32 | <0.001 |
| Q5 (Richest) | 1.40 | 1.31 | 1.50 | <0.001 | 1.33 | 1.26 | 1.41 | <0.001 |
| BMI |  |  |  |  |  |  |  |  |
| <18.5 kg/m2 | 0.94 | 0.90 | 0.99 | 0.024 | 0.93 | 0.86 | 1.01 | 0.074 |
| 18.5-22.9 kg/m2 | 1 (Reference) |  |  |  | 1 (Reference) |  |  |  |
| 23.0-24.9 kg/m2 | 1.06 | 1.03 | 1.10 | <0.001 | 1.08 | 1.04 | 1.13 | <0.001 |
| 25.0-27.4 kg/m2 | 1.09 | 1.05 | 1.13 | <0.001 | 1.08 | 1.04 | 1.12 | <0.001 |
| 27.5-29.9 kg/m2 | 1.14 | 1.09 | 1.19 | <0.001 | 1.12 | 1.07 | 1.18 | <0.001 |
| 30.0 kg/m2 | 1.17 | 1.11 | 1.24 | <0.001 | 1.18 | 1.12 | 1.24 | <0.001 |
| Tobacco, smoked | 0.98 | 0.95 | 1.01 | 0.132 | 0.96 | 0.93 | 1.00 | 0.027 |
| Tobacco, smokeless | 0.98 | 0.94 | 1.01 | 0.140 | 0.96 | 0.92 | 0.99 | 0.024 |
| Currently married | 1.20 | 1.16 | 1.24 | <0.001 | 1.18 | 1.13 | 1.22 | <0.001 |

Abbreviations: CI = Confidence Interval; Q = Quintile

^16^ These covariate-unadjusted regressions contained one of the listed indicators in the table (age group, wealth quintile, education, BMI, Tobacco smoked, Tobacco smokeless, marital status) and a binary indicator for each district (district-level fixed effects) as explanatory variables. Standard errors were adjusted for clustering at the level of the primary sampling unit. Sampling weights were not used.

Aware

|  | Rural | | | | Urban | | | |
| --- | --- | --- | --- | --- | --- | --- | --- | --- |
|  | Relative Risk | 95% CI (low) | 95% CI (high) | P Value | Relative Risk | 95% CI (low) | 95% CI (high) | P Value |
| Age group, y |  |  |  |  |  |  |  |  |
| 15-19 | 1 (Reference) |  |  |  | 1 (Reference) |  |  |  |
| 20-24 | 0.94 | 0.83 | 1.05 | 0.282 | 0.92 | 0.79 | 1.08 | 0.300 |
| 25-29 | 1.00 | 0.90 | 1.12 | 0.944 | 1.00 | 0.86 | 1.15 | 0.972 |
| 30-34 | 0.99 | 0.89 | 1.11 | 0.876 | 1.01 | 0.88 | 1.16 | 0.911 |
| 35-40 | 1.00 | 0.90 | 1.12 | 0.967 | 0.98 | 0.86 | 1.13 | 0.826 |
| 40-44 | 1.05 | 0.94 | 1.18 | 0.374 | 1.12 | 0.97 | 1.28 | 0.124 |
| 45-49 | 1.08 | 0.97 | 1.20 | 0.162 | 1.23 | 1.08 | 1.41 | 0.002 |
| Education |  |  |  |  |  |  |  |  |
| Primary school unfinished | 1 (Reference) |  |  |  | 1 (Reference) |  |  |  |
| Primary school finished | 1.06 | 0.94 | 1.19 | 0.331 | 1.00 | 0.86 | 1.17 | 0.962 |
| Secondary school unfinished | 1.10 | 1.03 | 1.18 | 0.006 | 1.07 | 0.96 | 1.19 | 0.197 |
| Secondary school or above | 1.15 | 1.07 | 1.24 | <0.001 | 1.25 | 1.12 | 1.39 | <0.001 |
| Household wealth quintile |  |  |  |  |  |  |  |  |
| Q1 (Poorest) | 1 (Reference) |  |  |  | 1 (Reference) |  |  |  |
| Q2 | 1.14 | 1.01 | 1.28 | 0.030 | 1.15 | 1.03 | 1.28 | 0.013 |
| Q3 | 1.20 | 1.07 | 1.35 | 0.002 | 1.24 | 1.11 | 1.38 | <0.001 |
| Q4 | 1.25 | 1.11 | 1.41 | <0.001 | 1.27 | 1.13 | 1.43 | <0.001 |
| Q5 (Richest) | 1.29 | 1.15 | 1.45 | <0.001 | 1.38 | 1.22 | 1.55 | <0.001 |
| BMI |  |  |  |  |  |  |  |  |
| <18.5 kg/m2 | 1.14 | 1.06 | 1.24 | 0.001 | 1.07 | 0.93 | 1.23 | 0.339 |
| 18.5-22.9 kg/m2 | 1 (Reference) |  |  |  | 1 (Reference) |  |  |  |
| 23.0-24.9 kg/m2 | 0.97 | 0.91 | 1.04 | 0.436 | 0.96 | 0.88 | 1.05 | 0.386 |
| 25.0-27.4 kg/m2 | 0.96 | 0.89 | 1.04 | 0.334 | 1.00 | 0.92 | 1.09 | 0.934 |
| 27.5-29.9 kg/m2 | 0.96 | 0.87 | 1.07 | 0.454 | 1.12 | 1.01 | 1.24 | 0.025 |
| 30.0 kg/m2 | 1.18 | 1.04 | 1.33 | 0.011 | 1.22 | 1.08 | 1.37 | 0.001 |
| Tobacco, smoked | 1.03 | 0.98 | 1.09 | 0.224 | 0.98 | 0.91 | 1.05 | 0.528 |
| Tobacco, smokeless | 0.94 | 0.88 | 1.00 | 0.070 | 0.84 | 0.78 | 0.92 | <0.001 |
| Currently married | 1.06 | 1.00 | 1.13 | 0.047 | 1.09 | 1.01 | 1.17 | 0.026 |

Abbreviations: CI = Confidence Interval; Q = Quintile

^16^ These covariate-unadjusted regressions contained one of the listed indicators in the table (age group, wealth quintile, education, BMI, Tobacco smoked, Tobacco smokeless, marital status) and a binary indicator for each district (district-level fixed effects) as explanatory variables. Standard errors were adjusted for clustering at the level of the primary sampling unit. Sampling weights were not used.

Treated

|  | Rural | | | | Urban | | | |
| --- | --- | --- | --- | --- | --- | --- | --- | --- |
|  | Relative Risk | 95% CI (low) | 95% CI (high) | P Value | Relative Risk | 95% CI (low) | 95% CI(high) | P Value |
| Age group, y |  |  |  |  |  |  |  |  |
| 15-19 | 1 (Reference) |  |  |  | 1 (Reference) |  |  |  |
| 20-24 | 0.65 | 0.46 | 0.92 | 0.016 | 1.15 | 0.69 | 1.92 | 0.596 |
| 25-29 | 0.84 | 0.62 | 1.14 | 0.268 | 1.11 | 0.67 | 1.83 | 0.693 |
| 30-34 | 0.86 | 0.63 | 1.15 | 0.307 | 1.20 | 0.74 | 1.95 | 0.453 |
| 35-40 | 0.98 | 0.74 | 1.31 | 0.910 | 1.36 | 0.84 | 2.19 | 0.208 |
| 40-44 | 1.23 | 0.93 | 1.62 | 0.152 | 2.47 | 1.56 | 3.90 | <0.001 |
| 45-49 | 1.40 | 1.06 | 1.84 | 0.017 | 2.97 | 1.89 | 4.66 | <0.001 |
| Education |  |  |  |  |  |  |  |  |
| Primary school unfinished | 1 (Reference) |  |  |  | 1 (Reference) |  |  |  |
| Primary school finished | 0.93 | 0.70 | 1.23 | 0.611 | 1.12 | 0.77 | 1.63 | 0.542 |
| Secondary school unfinished | 0.98 | 0.84 | 1.15 | 0.829 | 0.97 | 0.76 | 1.24 | 0.796 |
| Secondary school or above | 1.16 | 0.98 | 1.38 | 0.085 | 1.23 | 0.96 | 1.56 | 0.096 |
| Household wealth quintile |  |  |  |  |  |  |  |  |
| Q1 (Poorest) | 1 (Reference) |  |  |  | 1 (Reference) |  |  |  |
| Q2 | 1.04 | 0.79 | 1.37 | 0.776 | 1.58 | 1.19 | 2.09 | 0.001 |
| Q3 | 1.24 | 0.95 | 1.61 | 0.110 | 1.65 | 1.25 | 2.18 | <0.001 |
| Q4 | 1.26 | 0.97 | 1.64 | 0.080 | 1.73 | 1.30 | 2.30 | <0.001 |
| Q5 (Richest) | 1.53 | 1.19 | 1.99 | 0.001 | 2.26 | 1.70 | 3.01 | <0.001 |
| BMI |  |  |  |  |  |  |  |  |
| <18.5 kg/m2 | 1.23 | 1.00 | 1.53 | 0.055 | 1.05 | 0.71 | 1.55 | 0.812 |
| 18.5-22.9 kg/m2 | 1 (Reference) |  |  |  | 1 (Reference) |  |  |  |
| 23.0-24.9 kg/m2 | 1.13 | 0.96 | 1.33 | 0.132 | 1.27 | 1.01 | 1.60 | 0.043 |
| 25.0-27.4 kg/m2 | 1.20 | 1.01 | 1.43 | 0.042 | 1.45 | 1.16 | 1.81 | 0.001 |
| 27.5-29.9 kg/m2 | 1.22 | 0.97 | 1.53 | 0.091 | 2.03 | 1.61 | 2.57 | <0.001 |
| 30.0 kg/m2 | 1.85 | 1.46 | 2.35 | <0.001 | 2.50 | 1.96 | 3.18 | <0.001 |
| Tobacco, smoked | 0.98 | 0.86 | 1.12 | 0.793 | 0.97 | 0.82 | 1.15 | 0.715 |
| Tobacco, smokeless | 0.90 | 0.78 | 1.05 | 0.170 | 0.74 | 0.61 | 0.88 | 0.001 |
| Currently married | 1.37 | 1.16 | 1.60 | <0.001 | 1.76 | 1.44 | 2.16 | <0.001 |

Abbreviations: CI = Confidence Interval; Q = Quintile

^16^ These covariate-unadjusted regressions contained one of the listed indicators in the table (age group, wealth quintile, education, BMI, Tobacco smoked, Tobacco smokeless, marital status) and a binary indicator for each district (district-level fixed effects) as explanatory variables. Standard errors were adjusted for clustering at the level of the primary sampling unit. Sampling weights were not used.

Controlled

|  | Rural | | | | Urban | | | |
| --- | --- | --- | --- | --- | --- | --- | --- | --- |
|  | Relative Risk | 95% CI (low) | 95% CI (high) | P Value | Relative Risk | 95% CI (low) | 95% CI (high) | P Value |
| Age group, y |  |  |  |  |  |  |  |  |
| 15-19 | 1 (Reference) |  |  |  | 1 (Reference) |  |  |  |
| 20-24 | 0.58 | 0.40 | 0.83 | 0.003 | 1.17 | 0.66 | 2.06 | 0.593 |
| 25-29 | 0.63 | 0.45 | 0.88 | 0.007 | 1.18 | 0.68 | 2.05 | 0.560 |
| 30-34 | 0.54 | 0.38 | 0.75 | <0.001 | 0.84 | 0.48 | 1.49 | 0.561 |
| 35-40 | 0.57 | 0.42 | 0.79 | 0.001 | 0.70 | 0.40 | 1.24 | 0.225 |
| 40-44 | 0.57 | 0.42 | 0.80 | 0.001 | 1.49 | 0.88 | 2.54 | 0.141 |
| 45-49 | 0.70 | 0.51 | 0.95 | 0.022 | 1.65 | 0.97 | 2.81 | 0.065 |
| Education |  |  |  |  |  |  |  |  |
| Primary school unfinished | 1 (Reference) |  |  |  | 1 (Reference) |  |  |  |
| Primary school finished | 0.96 | 0.63 | 1.46 | 0.848 | 1.08 | 0.61 | 1.93 | 0.788 |
| Secondary school unfinished | 1.16 | 0.92 | 1.46 | 0.207 | 0.98 | 0.67 | 1.42 | 0.896 |
| Secondary school or above | 1.30 | 1.02 | 1.66 | 0.034 | 1.28 | 0.90 | 1.83 | 0.167 |
| Household wealth quintile |  |  |  |  |  |  |  |  |
| Q1 (Poorest) | 1 (Reference) |  |  |  | 1 (Reference) |  |  |  |
| Q2 | 1.01 | 0.71 | 1.43 | 0.960 | 1.68 | 1.10 | 2.56 | 0.015 |
| Q3 | 1.17 | 0.84 | 1.63 | 0.366 | 1.51 | 0.98 | 2.33 | 0.061 |
| Q4 | 1.12 | 0.80 | 1.56 | 0.521 | 1.71 | 1.11 | 2.63 | 0.015 |
| Q5 (Richest) | 1.39 | 1.00 | 1.93 | 0.052 | 2.07 | 1.36 | 3.14 | 0.001 |
| BMI |  |  |  |  |  |  |  |  |
| <18.5 kg/m2 | 1.50 | 1.17 | 1.92 | 0.001 | 1.47 | 0.95 | 2.28 | 0.084 |
| 18.5-22.9 kg/m2 | 1 (Reference) |  |  |  | 1 (Reference) |  |  |  |
| 23.0-24.9 kg/m2 | 0.97 | 0.78 | 1.21 | 0.779 | 1.16 | 0.85 | 1.59 | 0.357 |
| 25.0-27.4 kg/m2 | 0.83 | 0.65 | 1.08 | 0.166 | 1.11 | 0.80 | 1.53 | 0.527 |
| 27.5-29.9 kg/m2 | 0.62 | 0.43 | 0.90 | 0.013 | 1.80 | 1.28 | 2.52 | 0.001 |
| 30.0 kg/m2 | 1.29 | 0.90 | 1.85 | 0.164 | 2.25 | 1.56 | 3.26 | <0.001 |
| Tobacco, smoked | 0.98 | 0.81 | 1.17 | 0.804 | 0.96 | 0.75 | 1.22 | 0.719 |
| Tobacco, smokeless | 0.81 | 0.67 | 0.99 | 0.044 | 0.66 | 0.50 | 0.87 | 0.003 |
| Currently married | 0.88 | 0.73 | 1.06 | 0.186 | 1.18 | 0.91 | 1.53 | 0.211 |

Abbreviations: CI = Confidence Interval; Q = Quintile

^16^ These covariate-unadjusted regressions contained one of the listed indicators in the table (age group, wealth quintile, education, BMI, Tobacco smoked, Tobacco smokeless, marital status) and a binary indicator for each district (district-level fixed effects) as explanatory variables. Standard errors were adjusted for clustering at the level of the primary sampling unit. Sampling weights were not used.

# Table AF. Predictors of reaching each cascade step using covariate-adjusted poisson regressions with restricted cubic splines of five knots for the continuous variables age, wealth index and BMI^17^

Screened

|  | Rural |  |  |  | Urban |  |  |  |
| --- | --- | --- | --- | --- | --- | --- | --- | --- |
|  | Relative Risk | 95% CI (low) | 95% CI (high) | P Value | Relative Risk | 95% CI (low) | 95% CI (high) | P Value |
| Age group, y |  |  |  |  |  |  |  |  |
| Age group | 1(Reference) |  |  |  | 1(Reference) |  |  |  |
| Age group' | 1.02 | 1.02 | 1.03 | <0.001 | 1.01 | 1.01 | 1.02 | <0.001 |
| Age group'' | 0.95 | 0.93 | 0.96 | <0.001 | 0.97 | 0.95 | 0.99 | 0.004 |
| Age group''' | 1.28 | 1.16 | 1.42 | <0.001 | 1.13 | 1.01 | 1.26 | 0.028 |
| Age group'''' | 0.73 | 0.60 | 0.88 | 0.001 | 0.86 | 0.69 | 1.08 | 0.186 |
| Education |  |  |  |  |  |  |  |  |
| Primary school unfinished | 1(Reference) |  |  |  | 1(Reference) |  |  |  |
| Primary school finished | 1.04 | 1.01 | 1.07 | 0.011 | 1.02 | 0.98 | 1.07 | 0.330 |
| Secondary school unfinished | 1.07 | 1.05 | 1.09 | <0.001 | 1.03 | 1.00 | 1.06 | 0.047 |
| Secondary school or above | 1.10 | 1.07 | 1.13 | <0.001 | 1.06 | 1.03 | 1.10 | <0.001 |
| Wealth Index |  |  |  |  |  |  |  |  |
| Wealth Index | 1(Reference) |  |  |  | 1(Reference) |  |  |  |
| Wealth Index' | 1.25 | 1.16 | 1.34 | <0.001 | 1.06 | 0.98 | 1.15 | 0.133 |
| Wealth Index'' | 0.42 | 0.28 | 0.63 | <0.001 | 0.88 | 0.73 | 1.05 | 0.158 |
| Wealth Index''' | 9.29 | 2.23 | 38.74 | 0.002 | 2.08 | 0.46 | 9.48 | 0.343 |
| Wealth Index'''' | 0.16 | 0.03 | 0.93 | 0.041 | 0.32 | 0.01 | 9.14 | 0.504 |
| BMI |  |  |  |  |  |  |  |  |
| BMI | 1(Reference) |  |  |  | 1(Reference) |  |  |  |
| BMI' | 0.99 | 0.98 | 1.00 | 0.143 | 1.00 | 0.99 | 1.01 | 0.969 |
| BMI'' | 1.03 | 0.94 | 1.13 | 0.481 | 1.00 | 0.92 | 1.08 | 0.941 |
| BMI''' | 0.95 | 0.68 | 1.34 | 0.791 | 1.02 | 0.71 | 1.47 | 0.908 |
| BMI'''' | 0.98 | 0.65 | 1.46 | 0.906 | 0.97 | 0.60 | 1.57 | 0.914 |
| Tobacco, smoked | 0.99 | 0.96 | 1.03 | 0.682 | 0.99 | 0.94 | 1.04 | 0.575 |
| Tobacco, smokeless | 0.99 | 0.96 | 1.01 | 0.264 | 0.98 | 0.94 | 1.02 | 0.249 |
| Currently married | 1.12 | 1.09 | 1.15 | <0.001 | 1.08 | 1.05 | 1.12 | <0.001 |
| Female | 1.24 | 1.21 | 1.28 | <0.001 | 1.18 | 1.14 | 1.22 | <0.001 |

^17^ For five knots, the outer quantiles used are 0.05 and 0.95. The knots are equally spaced between these on the quantile scale.

Aware

|  | Rural |  |  |  | Urban |  |  |  |
| --- | --- | --- | --- | --- | --- | --- | --- | --- |
|  | Relative Risk | 95% CI (low) | 95% CI (high) | P Value | Relative Risk | 95% CI (low) | 95% CI (high) | P Value |
| Age group, y |  |  |  |  |  |  |  |  |
| Age group | 1(Reference) |  |  |  | 1(Reference) |  |  |  |
| Age group' | 1.01 | 1.00 | 1.01 | 0.018 | 1.00 | 0.99 | 1.01 | 0.471 |
| Age group'' | 0.95 | 0.93 | 0.97 | <0.001 | 0.97 | 0.94 | 1.00 | 0.024 |
| Age group''' | 1.32 | 1.16 | 1.49 | <0.001 | 1.18 | 1.03 | 1.35 | 0.015 |
| Age group'''' | 0.69 | 0.54 | 0.88 | 0.003 | 0.80 | 0.60 | 1.06 | 0.117 |
| Education |  |  |  |  |  |  |  |  |
| Primary school unfinished | 1(Reference) |  |  |  | 1(Reference) |  |  |  |
| Primary school finished | 1.07 | 1.03 | 1.11 | <0.001 | 1.02 | 0.96 | 1.08 | 0.475 |
| Secondary school unfinished | 1.11 | 1.08 | 1.14 | <0.001 | 1.05 | 1.01 | 1.09 | 0.015 |
| Secondary school or above | 1.15 | 1.11 | 1.19 | <0.001 | 1.09 | 1.05 | 1.14 | <0.001 |
| Wealth Index |  |  |  |  |  |  |  |  |
| Wealth Index | 1(Reference) |  |  |  | 1(Reference) |  |  |  |
| Wealth Index' | 1.33 | 1.20 | 1.46 | <0.001 | 1.02 | 0.93 | 1.13 | 0.643 |
| Wealth Index'' | 0.32 | 0.19 | 0.53 | <0.001 | 0.82 | 0.65 | 1.04 | 0.097 |
| Wealth Index''' | 14.98 | 2.40 | 93.58 | 0.004 | 2.66 | 0.39 | 17.96 | 0.316 |
| Wealth Index'''' | 0.16 | 0.02 | 1.48 | 0.106 | 0.30 | 0.00 | 20.36 | 0.576 |
| BMI |  |  |  |  |  |  |  |  |
| BMI | 1(Reference) |  |  |  | 1(Reference) |  |  |  |
| BMI' | 0.99 | 0.98 | 1.00 | 0.115 | 0.98 | 0.97 | 1.00 | 0.024 |
| BMI'' | 0.90 | 0.80 | 1.00 | 0.058 | 0.98 | 0.89 | 1.09 | 0.735 |
| BMI''' | 1.56 | 1.02 | 2.39 | 0.039 | 1.30 | 0.83 | 2.05 | 0.252 |
| BMI'''' | 0.64 | 0.38 | 1.05 | 0.079 | 0.63 | 0.34 | 1.14 | 0.123 |
| Tobacco, smoked | 1.04 | 0.99 | 1.09 | 0.104 | 1.03 | 0.96 | 1.10 | 0.404 |
| Tobacco, smokeless | 0.96 | 0.93 | 1.00 | 0.027 | 0.97 | 0.93 | 1.02 | 0.293 |
| Currently married | 1.15 | 1.11 | 1.18 | <0.001 | 1.13 | 1.09 | 1.18 | <0.001 |
| Female | 1.61 | 1.55 | 1.68 | <0.001 | 1.56 | 1.49 | 1.64 | <0.001 |

^17^ For five knots, the outer quantiles used are 0.05 and 0.95. The knots are equally spaced between these on the quantile scale.

Treated

|  | Rural |  |  |  | Urban |  |  |  |
| --- | --- | --- | --- | --- | --- | --- | --- | --- |
|  | Relative Risk | 95% CI (low) | 95% CI (high) | P Value | Relative Risk | 95% CI (low) | 95% CI (high) | P Value |
| Age group, y |  |  |  |  |  |  |  |  |
| Age group | 1(Reference) |  |  |  | 1(Reference) |  |  |  |
| Age group' | 1.00 | 0.99 | 1.01 | 0.800 | 0.98 | 0.97 | 1.00 | 0.064 |
| Age group'' | 1.03 | 0.99 | 1.07 | 0.141 | 1.07 | 1.01 | 1.14 | 0.021 |
| Age group''' | 0.93 | 0.74 | 1.18 | 0.553 | 0.91 | 0.70 | 1.18 | 0.473 |
| Age group'''' | 1.06 | 0.69 | 1.64 | 0.794 | 0.90 | 0.54 | 1.49 | 0.678 |
| Education |  |  |  |  |  |  |  |  |
| Primary school unfinished | 1(Reference) |  |  |  | 1(Reference) |  |  |  |
| Primary school finished | 0.99 | 0.93 | 1.07 | 0.845 | 1.03 | 0.94 | 1.14 | 0.497 |
| Secondary school unfinished | 1.02 | 0.97 | 1.07 | 0.432 | 0.96 | 0.89 | 1.02 | 0.177 |
| Secondary school or above | 1.01 | 0.94 | 1.08 | 0.778 | 0.94 | 0.87 | 1.02 | 0.119 |
| Wealth Index |  |  |  |  |  |  |  |  |
| Wealth Index | 1(Reference) |  |  |  | 1(Reference) |  |  |  |
| Wealth Index' | 1.19 | 1.00 | 1.42 | 0.053 | 0.97 | 0.80 | 1.18 | 0.735 |
| Wealth Index'' | 0.22 | 0.08 | 0.57 | 0.002 | 0.89 | 0.58 | 1.36 | 0.577 |
| Wealth Index''' | 58.64 | 2.11 | 1626.11 | 0.016 | 2.21 | 0.07 | 70.63 | 0.653 |
| Wealth Index'''' | 0.03 | 0.00 | 1.49 | 0.078 | 0.24 | 0.00 | 438.26 | 0.711 |
| BMI |  |  |  |  |  |  |  |  |
| BMI | 1(Reference) |  |  |  | 1(Reference) |  |  |  |
| BMI' | 0.97 | 0.94 | 0.99 | 0.010 | 0.98 | 0.95 | 1.01 | 0.254 |
| BMI'' | 1.03 | 0.84 | 1.27 | 0.755 | 1.03 | 0.84 | 1.26 | 0.773 |
| BMI''' | 1.33 | 0.61 | 2.88 | 0.472 | 1.69 | 0.71 | 4.00 | 0.234 |
| BMI'''' | 0.51 | 0.21 | 1.26 | 0.145 | 0.26 | 0.09 | 0.77 | 0.016 |
| Tobacco, smoked | 0.94 | 0.86 | 1.03 | 0.169 | 1.02 | 0.90 | 1.16 | 0.700 |
| Tobacco, smokeless | 0.99 | 0.93 | 1.05 | 0.762 | 1.00 | 0.92 | 1.09 | 0.969 |
| Currently married | 1.09 | 1.03 | 1.15 | 0.005 | 1.08 | 1.00 | 1.16 | 0.037 |
| Female | 1.78 | 1.66 | 1.91 | <0.001 | 1.64 | 1.50 | 1.79 | <0.001 |

^17^ For five knots, the outer quantiles used are 0.05 and 0.95. The knots are equally spaced between these on the quantile scale.

Controlled

|  | Rural |  |  |  | Urban |  |  |  |
| --- | --- | --- | --- | --- | --- | --- | --- | --- |
|  | Relative Risk | 95% CI (low) | 95% CI (high) | P Value | Relative Risk | 95% CI (low) | 95% CI (high) | P Value |
| Age group, y |  |  |  |  |  |  |  |  |
| Age group | 1(Reference) |  |  |  | 1(Reference) |  |  |  |
| Age group' | 1.00 | 0.98 | 1.01 | 0.767 | 0.98 | 0.96 | 1.00 | 0.039 |
| Age group'' | 0.98 | 0.94 | 1.03 | 0.383 | 0.98 | 0.91 | 1.05 | 0.489 |
| Age group''' | 1.08 | 0.82 | 1.44 | 0.582 | 1.38 | 0.99 | 1.91 | 0.057 |
| Age group'''' | 1.01 | 0.58 | 1.75 | 0.978 | 0.48 | 0.25 | 0.94 | 0.031 |
| Education |  |  |  |  |  |  |  |  |
| Primary school unfinished | 1(Reference) |  |  |  | 1(Reference) |  |  |  |
| Primary school finished | 0.99 | 0.91 | 1.09 | 0.879 | 1.04 | 0.91 | 1.19 | 0.522 |
| Secondary school unfinished | 1.04 | 0.98 | 1.11 | 0.196 | 0.94 | 0.86 | 1.03 | 0.192 |
| Secondary school or above | 1.06 | 0.98 | 1.16 | 0.157 | 0.94 | 0.85 | 1.04 | 0.243 |
| Wealth Index |  |  |  |  |  |  |  |  |
| Wealth Index | 1(Reference) |  |  |  | 1(Reference) |  |  |  |
| Wealth Index' | 1.11 | 0.91 | 1.37 | 0.307 | 0.89 | 0.70 | 1.13 | 0.328 |
| Wealth Index'' | 0.35 | 0.11 | 1.13 | 0.080 | 0.71 | 0.40 | 1.24 | 0.229 |
| Wealth Index''' | 10.54 | 0.17 | 638.49 | 0.261 | 23.12 | 0.23 | 2279.99 | 0.180 |
| Wealth Index'''' | 0.25 | 0.00 | 37.14 | 0.586 | 0.00 | 0.00 | 21.15 | 0.174 |
| BMI |  |  |  |  |  |  |  |  |
| BMI | 1(Reference) |  |  |  | 1(Reference) |  |  |  |
| BMI' | 0.96 | 0.93 | 0.98 | 0.003 | 0.98 | 0.94 | 1.01 | 0.181 |
| BMI'' | 0.96 | 0.75 | 1.22 | 0.713 | 0.89 | 0.69 | 1.14 | 0.358 |
| BMI''' | 1.23 | 0.48 | 3.10 | 0.668 | 2.76 | 0.93 | 8.18 | 0.067 |
| BMI'''' | 0.95 | 0.31 | 2.91 | 0.935 | 0.19 | 0.05 | 0.76 | 0.019 |
| Tobacco, smoked | 1.01 | 0.90 | 1.14 | 0.803 | 0.98 | 0.83 | 1.17 | 0.861 |
| Tobacco, smokeless | 0.94 | 0.87 | 1.02 | 0.151 | 1.02 | 0.91 | 1.15 | 0.695 |
| Currently married | 1.09 | 1.02 | 1.18 | 0.017 | 1.11 | 1.01 | 1.23 | 0.028 |
| Female | 2.02 | 1.83 | 2.22 | <0.001 | 1.95 | 1.73 | 2.20 | <0.001 |

^17^ For five knots, the outer quantiles used are 0.05 and 0.95. The knots are equally spaced between these on the quantile scale.

# Fig A. Flowchart of patients lost in hypertension care (using sampling weights)


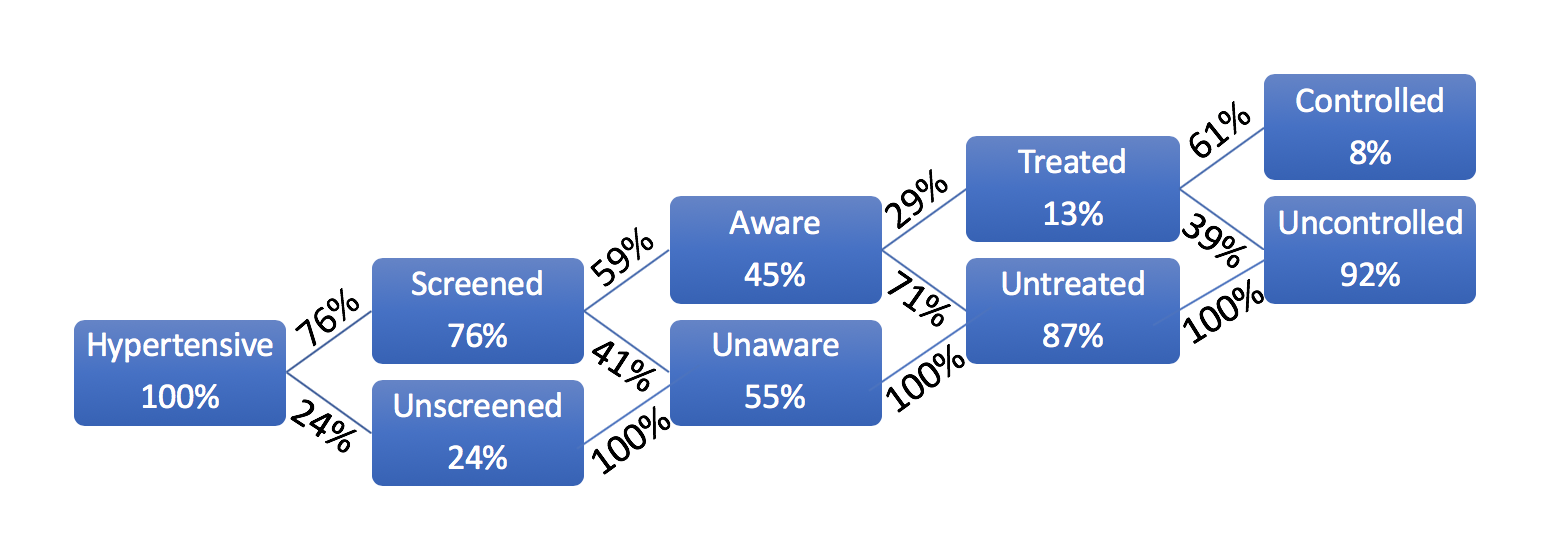


# Fig B. Map of crude hypertension prevalence by state and sex^1,2^


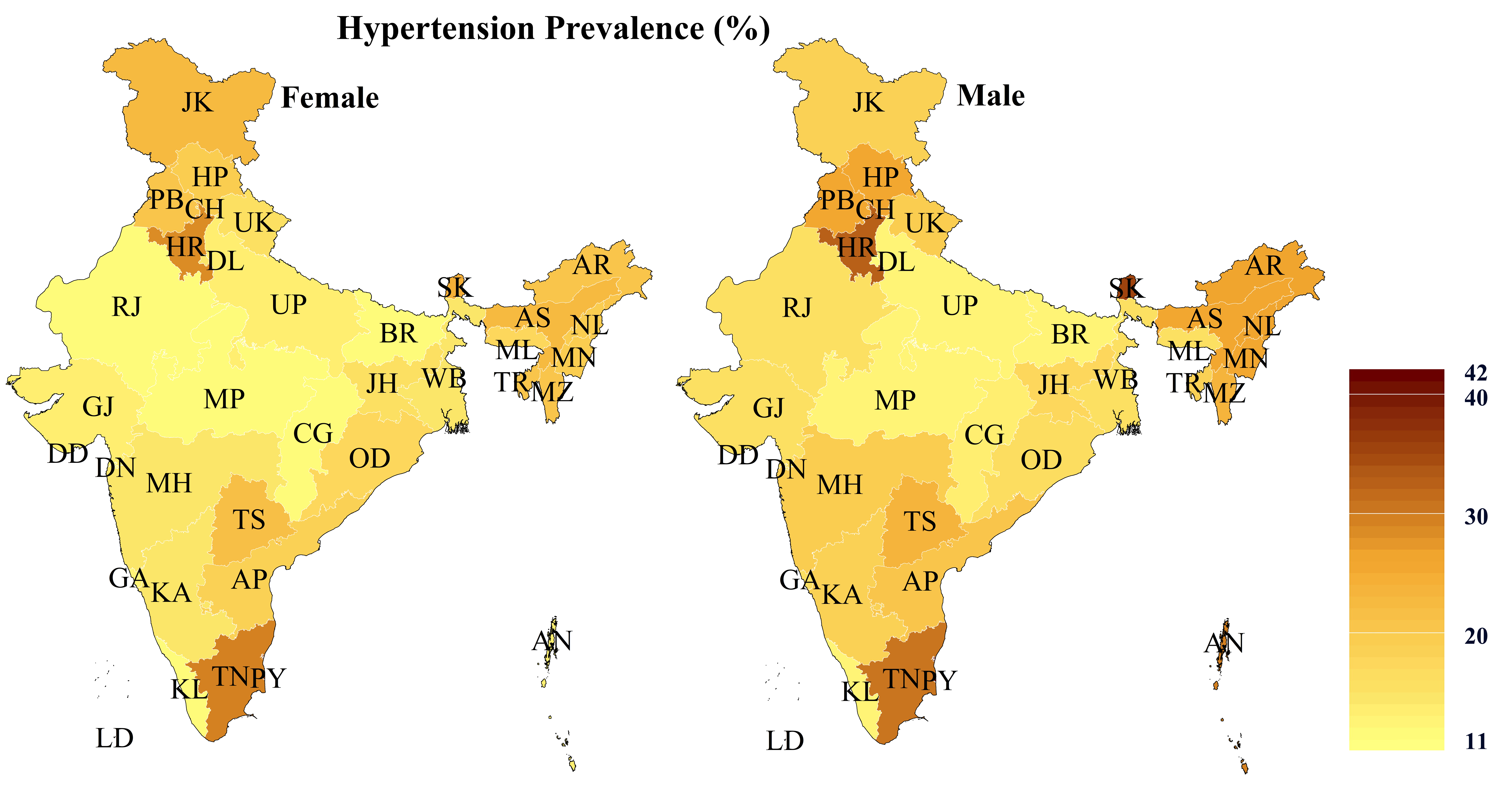


^1^ Some Union Territories are not visible due to their small size.

^2^ AP indicates Andhra Pradesh; AR, Arunachal Pradesh; AS, Assam; BR, Bihar; CT, Chhattisgarh; DL, Delhi; GA, Goa; HR, Haryana; HP, Himachal Pradesh; JH, Jharkhand; KA, Karnataka; KL, Kerala; MP, Madhya Pradesh; MH, Maharashtra; MN, Manipur; ML, Meghalaya; MZ, Mizoram; NL, Nagaland; OD, Odisha (Orissa); PB, Punjab; RJ, Rajasthan; SK, Sikkim; TN, Tamil Nadu; TS, Telangana State; TR, Tripura; UP, Uttar Pradesh; UK, Uttarakhand (Uttaranchal); WB, West Bengal.

# Fig C. Percent that reached each cascade step by rural-urban residence, sex, age group, and household wealth quintile


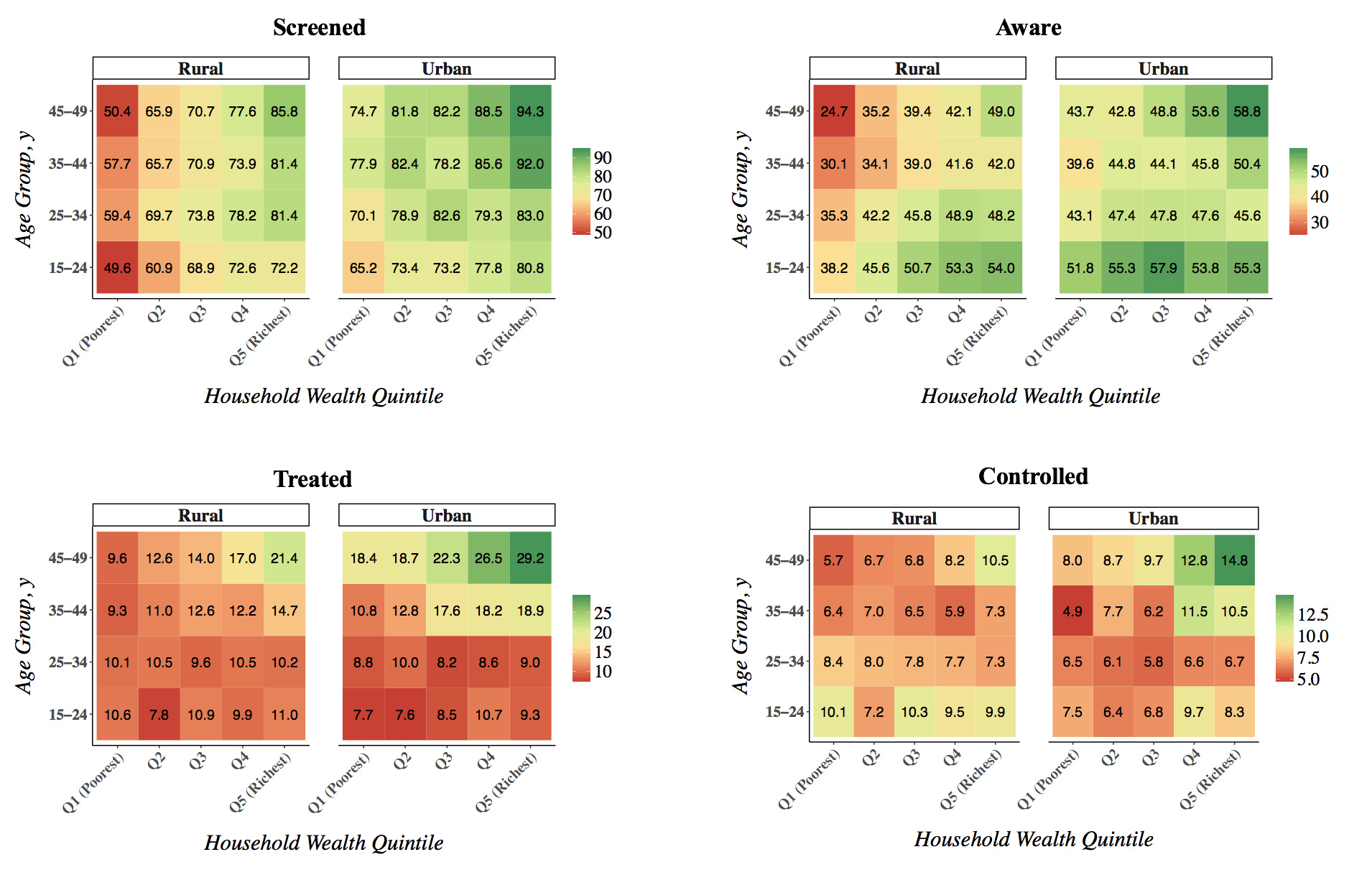


**References**

1. International Institute for Population Sciences Mumbai. National Family Health Survey 2015-2016 (NFHS-4) Clinical Anthropometric Biochemical (CAB) Manual, <http://rchiips.org/NFHS/NFHS4/manual/NFHS-4%20Biomarker%20Field%20Manual.pdf>. 2014.

2. International Institute for Population Sciences Mumbai. National Family Health Survey 2015-2016 (NFHS-4) Interviewer Manual, <http://rchiips.org/NFHS/NFHS4/manual/NFHS-4%20Interviewer%20Manual.pdf>.

. 2014.

3. International Institute for Population Sciences Mumbai. National Family Health Survey 2015-2016 (NFHS-4) Supervisor Manual, <http://rchiips.org/NFHS/NFHS4/manual/NFHS-4%20Supervisor%20Manual.pdf>.

4. International Institute for Population Sciences Mumbai. International Institute for Population Sciences Mumbai. National Family Health Survey 2015-2016 (NFHS-4) Man’s Questionnaire, <http://rchiips.org/NFHS/NFHS4/schedules/NFHS-4Mans.pdf>.

5. International Institute for Population Sciences Mumbai. National Family Health Survey 2015-2016 (NFHS-4) Biomarker Questionnaire, <http://rchiips.org/NFHS/NFHS4/schedules/NFHS-4Biomarker.pdf>. 2014.

6. International Institute for Population Sciences Mumbai. National Family Health Survey 2015-2016 (NFHS-4) Household Questionnaire, <http://rchiips.org/NFHS/NFHS4/schedules/NFHS-4Household.pdf>. 2014.

7. International Institute for Population Sciences Mumbai. International Institute for Population Sciences Mumbai. National Family Health Survey 2015-2016 (NFHS-4) Woman’s Questionnaire, <http://rchiips.org/NFHS/NFHS4/schedules/NFHS-4Womans.pdf>.

8. International Institute for Population Sciences Mumbai. National Family Health Survey (NFHS-4), 2015-16: India Report, available from <http://rchiips.org/nfhs/NFHS-4Reports/India.pdf> 2017.

9. Government of India (Ministry of Health and Family Welfare). State/ UT-wise Rural and Urban Population as Per Census during 2001 and 2011. <https://datagovin/resources/state-ut-wise-rural-and-urban-population-census-during-2001-and-2011>. . 2011.

10. Institute for Health Metrics and Evaluation. GBD Compare online visualization hub. Seattle, WA: IHME, University of Washington. Available from <http://vizhubhealthdataorg/gbd-compare> (Accessed [08-31-2018]). 2015
